# Single-Benzene-Based Clickable Fluorophores for In Vitro and In Vivo Bioimaging

**DOI:** 10.1002/slct.202405738

**Published:** 2025-02-27

**Authors:** Raja Mohanrao, Clyde S. Pinto, Andrejus Suchenko, Guy J. Clarkson, Martin Wills, Stefan Roesner, Michael Shipman, Mohan K. Balasubramanian

**Affiliations:** a Centre for Mechanochemical Cell Biology and Warwick Medical School, Division of Biomedical Science, University of Warwick, CoventryCV4 7AL, UK; b Department of Chemistry, University of Warwick, Gibbet Hill Road, CoventryCV4 7AL, UK; c School of Pharmacy and Biomolecular Sciences, Liverpool John Moores University, Byrom Street, LiverpoolL3 3AF, UK; d The Palatine Centre, Durham University, Stockton Road, Durham DH1 3LE, UK

**Keywords:** Bioconjugates, Bioimaging, Chromophores, Fluorescent probes, Single-benzene fluorophores

## Abstract

A series of miniaturized, clickable single-benzene-based fluorophores derived from tetrafluoroterephthalonitrile is reported. Fluorophores based on a tetrahydroquinoxaline skeleton exhibited improved photophysical properties due to enhanced electron delocalization between donor and acceptor groups compared to those with a dihydro[1,4]thiazine skeleton. These easily synthesized clickable fluorophores were successfully applied in both in vitro and in vivo bioimaging following protein conjugation.

## 1 Introduction

The ideal properties of fluorophores for bioimaging include compact size, facile synthesis, tunability of absorption and emission wavelengths ranging from UV to far IR, large Stokes shift, high quantum yield, and good solubility in aqueous media.^[1]^ Typically, polyaromatic *π*-conjugated fluorophores often suffer from poor solubility due to their tendency to aggregate, and they often require complex, multistep synthesis and purification.^[2,3]^ Moreover, the presence of a large fluorophore can potentially disrupt the properties and biological function of target molecules.^[4]^ Owing to their simple aromatic skeleton, the design and synthesis of single-benzene-based fluorophores (SBBFs) have attracted considerable attention.^[5]^ In contrast to large polyaromatic fluorophores, SBBFs contain electron-donor (D)−acceptor (A) functional groups incorporated into a compact benzene ring.^[6–13]^ Because of their facile synthesis, various types of SBBFs have recently been developed and utilized in bioimaging applications.^[4,14–17]^ In addition, their emission can easily be tuned by varying the substituents on the arene ring.^[18,19]^

**Figure 1:**
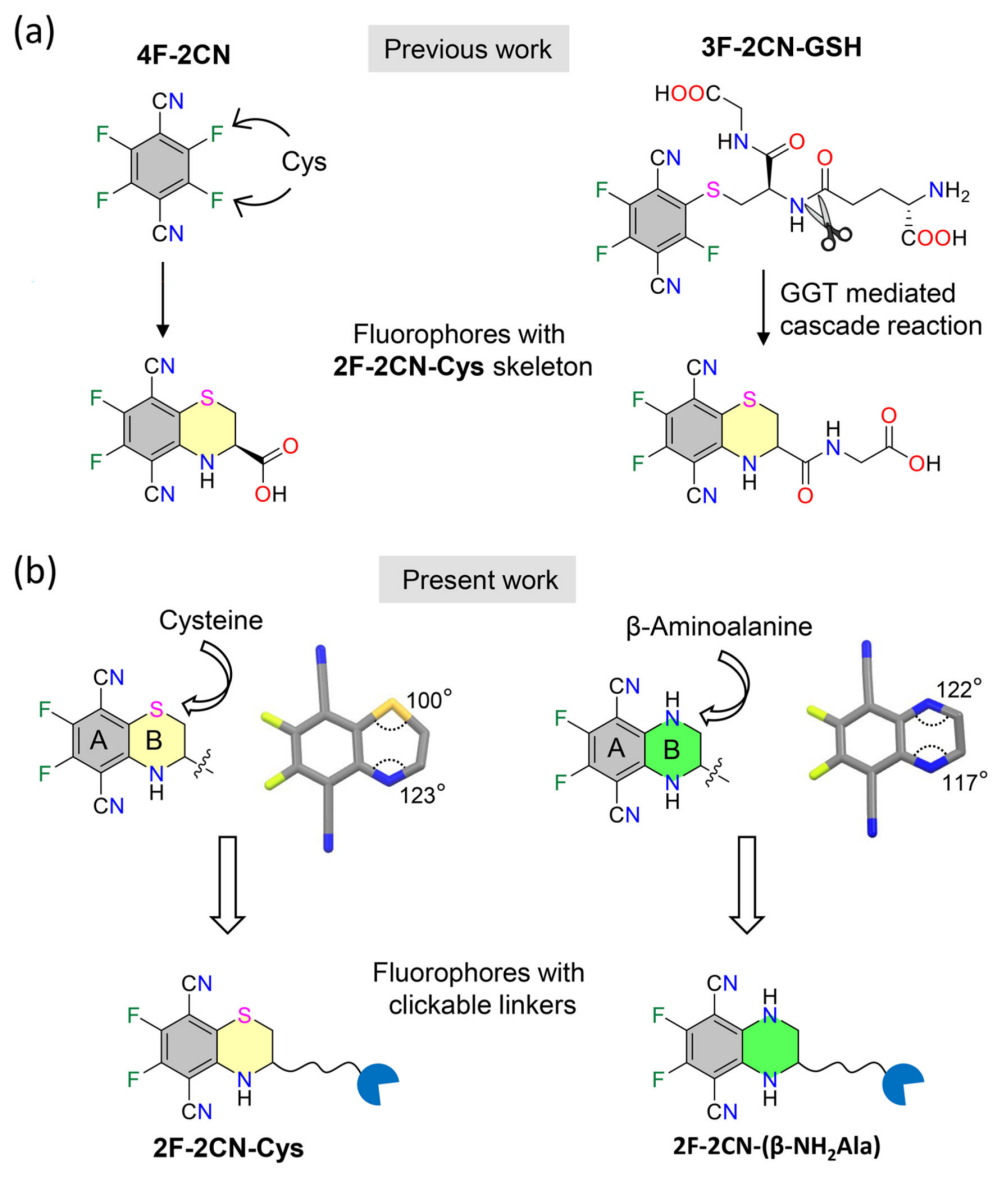
a) Reported fluorophores with **2F-2CN-Cys** skeleton. b) This work: Comparison of the **2F-2CN** fluorophores based on cysteine and *β*-aminoalanine and their derivatives with clickable linkers.

Zhang and coworkers reported the simple SBBF precursor tetrafluoroterephthalonitrile (**4F-2CN**), which was able to efficiently visualize and differentiate the common biological thiols cysteine (Cys), homocysteine (Hcy), and glutathione (GSH) (Figure 1a).^[20]^ The product from the reaction of **4F-2CN** with Cys, **2F-2CN-Cys**, displayed two-photon fluorescence properties. Subsequently, Huo and coworkers designed the *γ*-glutamyl transpeptidase (GGT) activated, water-soluble, two-photon fluorescent probe **3F-2CN-GSH**. This probe could be cleaved in situ when exposed to GGT-overexpressing cancer cells, forming a fluorophore with a **2F-2CN-Cys** skeleton (Figure 1a).^[21]^ Thus, **3F-2CN-GSH** has the potential to distinguish cancer cells from normal cells. Taking inspiration from these reports and the work of Banerjee and coworkers,^[22,23]^ we designed a **4F-2CN**-based fluorophore, where ring B is made from *β*-aminoalanine instead of cysteine (Figure 1b).

We hypothesized that nitrogen in the ring would promote greater planarity and conjugation compared to sulfur, leading to increased charge delocalization between the donor and acceptor groups, ultimately enhancing the fluorescence properties.^[22−25]^ Herein, we report the synthesis of four **2F-2CN-Cys** and **2F-2CN-(*β*-NH**_**2**_**Ala)** analogs appended to maleimide, which are suitable for conjugation to proteins through the thiol-ene click reaction. We assessed their photophysical properties in ethanol and aqueous solution and demonstrated the application of these miniaturized fluorophores in the bioimaging of actin filaments and zebrafish embryos.

## 2 Results and Discussion

**Scheme 1:**
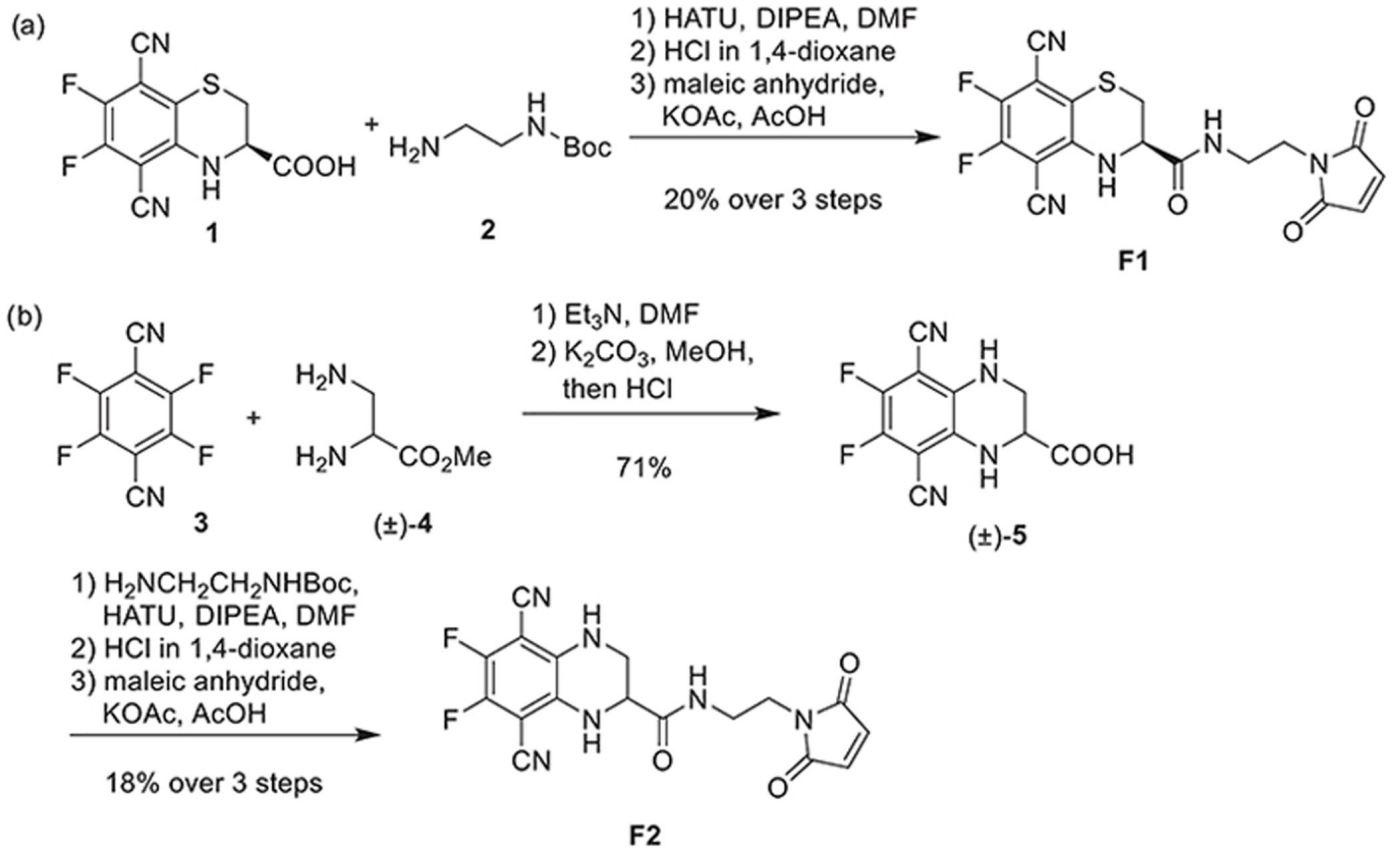
Synthesis of fluorophore maleimides **F1** and **F2**.

We first synthesized **2F-2CN-Cys** (**1**)^[20]^ and incorporated a maleimide functional group in three synthetic steps (Scheme 1a). First, carboxylic acid **1** was coupled with Boc-protected amine **2**. After further deprotection, the maleimide moiety was incorporated to obtain fluorophore **F1**, for which we successfully obtained a crystal structure.^[26]^ The maleimide unit in the SBBF allows for site-specific thiol-ene click coupling with proteins through thiol-containing amino acids. The *β*-alanine analog **F2** was synthesized using a similar strategy (Scheme 1b). We prepared the carboxylic acid derivative (±)-**5** by treating **4F-2CN** (**3**) with 1,2-diaminopropionate (±)-**4**, followed by saponification. Following the same strategy as for **F1**, the maleimide group was attached to (±)-**5** to yield clickable precursor **F2**. As discussed above, we anticipated that replacing sulfur with nitrogen in **F2** would improve planarity and electron delocalization, thereby modifying the photophysical properties of the fluorophore.

**Scheme 2:**
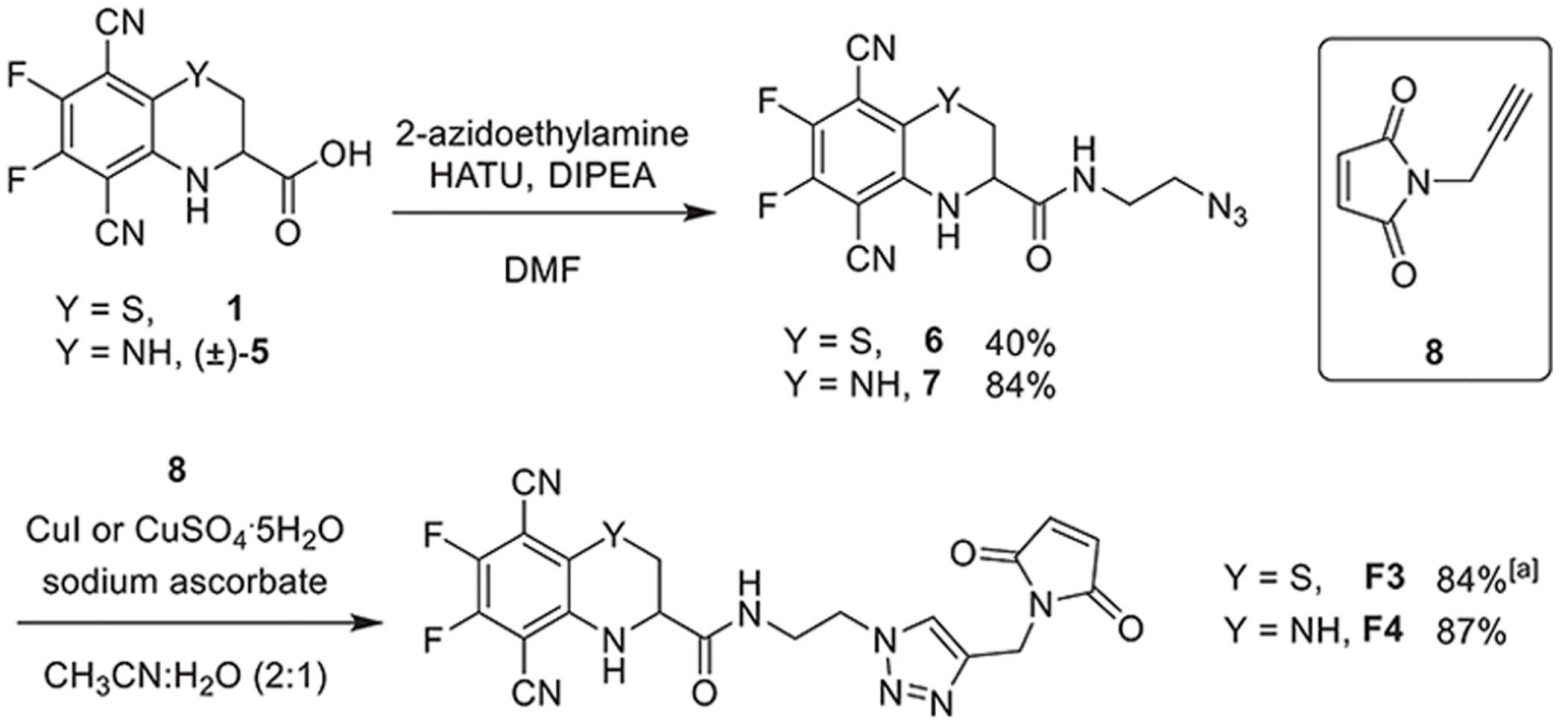
Synthesis of fluorophore-triazole-maleimides **F3** and **F4**. (a) Prepared as *R*-enantiomer.

In addition, we prepared clickable azide-based fluorophores **6** (crystal structure obtained)^[26]^ and **7** by reacting (*R*)-**1** and (±)-**5** with 2-azidoethylamine. These fluorophores were then coupled with maleimide-alkyne **8** using Cu-catalyzed azide-alkyne cycloaddition (CuAAC) chemistry (Scheme 2). The resulting fluorophore maleimides, **F3** and **F4**, containing triazole linkers, were thus also available for further protein conjugation.

**Figure 2:**
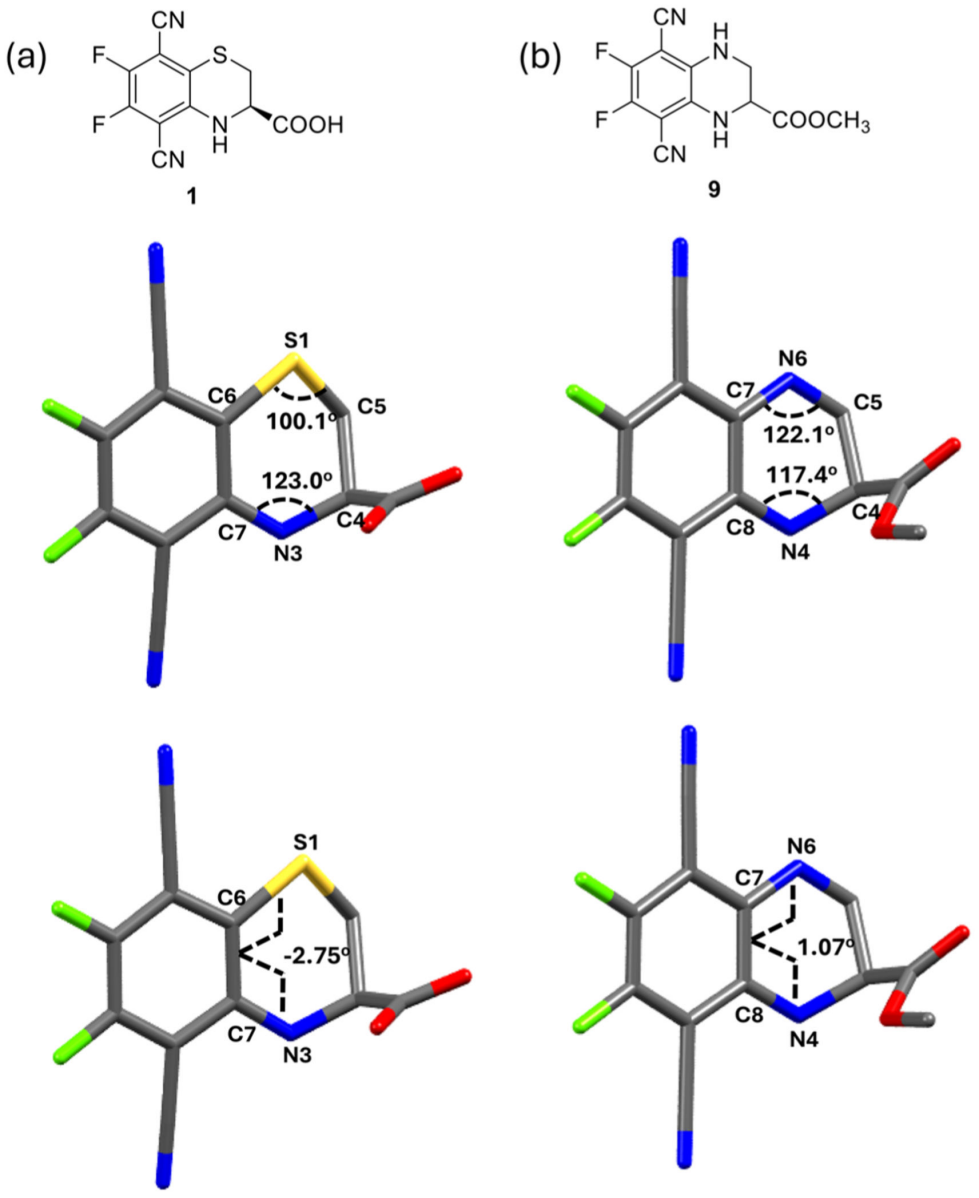
Crystal structures of a) **1** (reported)^[20]^ and b) **9**, including bond angles and dihedral angles. Hydrogen atoms are omitted for clarity.

We were able to obtain a crystal structure of compound **9**,^[26]^ the methyl ester of **5**, and compared it with the reported solidstate structure of **1** (Figure 2).^[20]^ In compound 1, the bond angles C6-S1-C5 and C7-N3-C4 are 100.1° and 123.0°, respectively, while the dihedral angle of S1-C6-C7-N3 is −2.75°. In comparison, compound **9** exhibits bond angles of 122.1° for C7-N6-C5 and 117.4° for C8-N4-C4, with a dihedral angle of N6-C7-C8-N4 being 1.07°. This indicates that the two nitrogen atoms in **9** adopt a more planar conformation compared to the sulfur–nitrogen combination in fluorophore **1**. Recent reports suggest that secondary amines in plane with terephthalonitriles (**TN**) exhibit enhanced emission properties.^[23]^

Next, we studied the photophysical properties of the fluorophore maleimides **F1**−**F4** in ethanol (Table 1). Absorption spectra showed maxima (λ_max,abs_) between 410 and 415 nm with molar extinction coefficients (*ε*) ranging from 5300 to 11,305 L mol^−1^ cm^−1^. The corresponding emission maxima (λ_max,em_) were measured between 480 and 490 nm when excited at λ_max,abs_. Quantum yields Φ_F_ ranged from 6.6% for compound **F1** up to 18.7% for maleimide **F4**. For both pairs of compounds, higher quantum yields were measured for the dinitrogen fluorophores **F2** and **F4** compared to their sulfur-containing counterparts, **F1** and **F3**.^[28]^

**Scheme 3:**
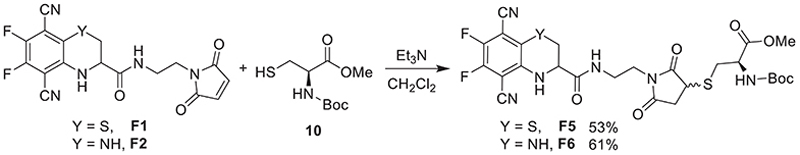
Reaction of fluorophore maleimides **F1** and **F2** with cysteine derivative **10**.

It is well established that a maleimide linker attached to a fluorophore can quench fluorescence through intramolecular charge transfer (ICT) or photoinduced electron transfer (PET) from the fluorophore to the maleimide double bond.^[29,30]^ However, when maleimides react with thiols, the double bond becomes saturated, preventing fluorescence quenching. To examine the impact of protein conjugation via a thiol on fluorophore performance, we coupled maleimide **F1** and **F2** with cysteine derivative **10** to yield **F5** and **F6** (Scheme 3). Similar to **F1, F5** exhibited nearly identical excitation and emission maxima with a molar extinction coefficient value of 6169 L mol^−1^ cm^−1^ (Table 1). However, the quantum yield (Φ_F_) for **F5** increased nearly fourfold (23.0%). Similarly, the nitrogen-containing derivative **F6** displayed excitation and emission maxima comparable to its precursor **F2**. In this case, the quantum yield Φ_F_ for **F6** was even higher, increasing nearly six-fold to 51.3%.

Next, we measured the photophysical properties of fluorophore derivatives **F1**−**F6** in aqueous solution (Table 2) to assess how these dye molecules would perform in vitro and in vivo imaging. As expected, the absorption maxima (λ_max,abs_) and emission maxima (λ_max,em_) were similar to those in ethanolic solution, ranging from 407 to 413 nm for λ_max,abs_ and from 488 to 492 nm for *λ*_max,em_. The molar extinction coefficients (*ε*) in water at λ_max,abs_, however, were significantly lower than those measured in ethanol. For example, while *ε* in ethanol (*ε*_EtOH_) for fluorophore **F1** was measured at 11,305 L mol^−1^ cm^−1^, the value in water (*ε*_water_) for the same compound was approximately halved to 5568 L mol^−1^ cm^−1^. This trend was consistent across all fluorophores. Finally, we determined the quantum yields (Φ_F_) for compounds **F1**−**F6** in aqueous solution. For **F1**−**F4**, the quantum yield values were similar to those in ethanol. However, while thiol coupling for **F5** and **F6** resulted in a notable increase in quantum efficiency in ethanol (see Table 1), this increase in water was only moderate, with Φ_F_ values of 16.6% for **F5** (compared to 23.0% in ethanol) and 16.9% for **F6** (compared to 51.6% in ethanol). Overall, while the fluorophores displayed reasonable photophysical properties in aqueous solution, their properties as fluorescent dyes were enhanced in ethanol.

**Figure 3:**
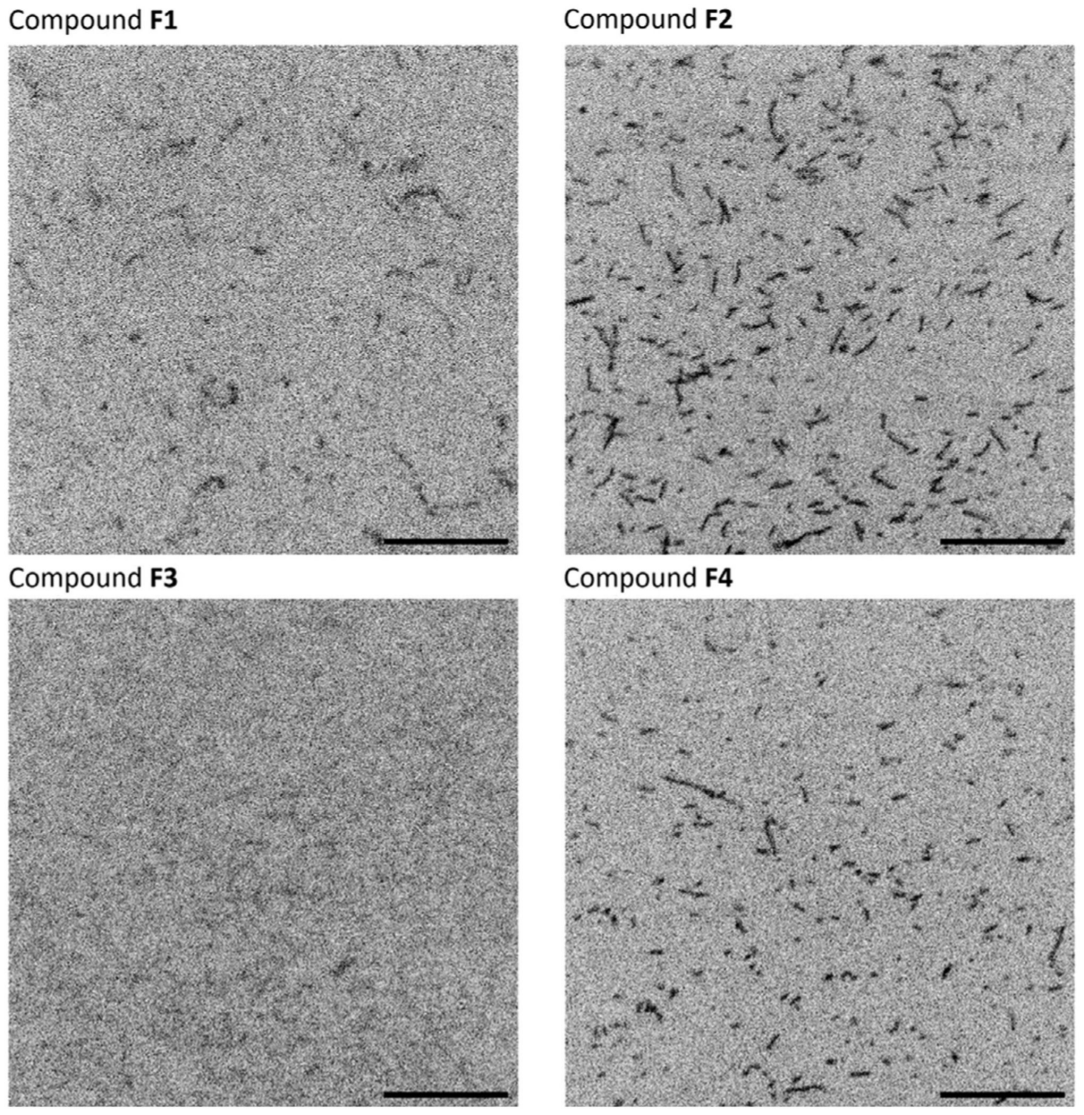
In vitro imaging of actin filaments excited at 405 nm and labelled with compounds **F1, F2, F3**, and **F4**. Purified *β*-actin proteins labelled with fluorescent compounds were polymerized. The filaments were imaged with a spinning disk confocal fluorescence microscope. Scale bars are 10 μm.^[28]^

To assess the potential of these fluorescent small molecules for in vitro and in vivo protein bioimaging, we developed two assays. In the first assay, we labelled the cytoskeletal protein human *β*-actin, which assembles into filamentous polymers.^[31,32]^ Filaments composed of monomeric actins can be readily visualized using conventional epifluorescence and/or total internal reflection microscopy. Purified *β*-actin was polymerized and subsequently labelled with compounds **F1**–**F4** through specific reaction of the native Cys 374 residue in actin with the maleimide moiety of **F1**−**F4**.^[33]^ Actin bound to **F1, F2, F3**, and **F4** was polymerized in vitro and imaged by spinning disk confocal microscopy. The fluorophores were excited with a 405 nm laser, and emissions were recorded using an EMCCD camera through a GFP emission filter, allowing detection wavelengths of 505 nm and above.^[34]^ Under these imaging conditions, polymers labelled with compounds **F2** and **F4** were readily detectable (Figure 3). In contrast, those labelled with compound **F1** showed weaker fluorescence, and the signal from filaments labelled with compound **F3** was below detectable levels. The fluorescence intensities of filaments labelled with compounds **F1, F2**, and **F4** were 1024, 2112, and 1286 RFU, respectively.^[28]^

**Figure 4:**
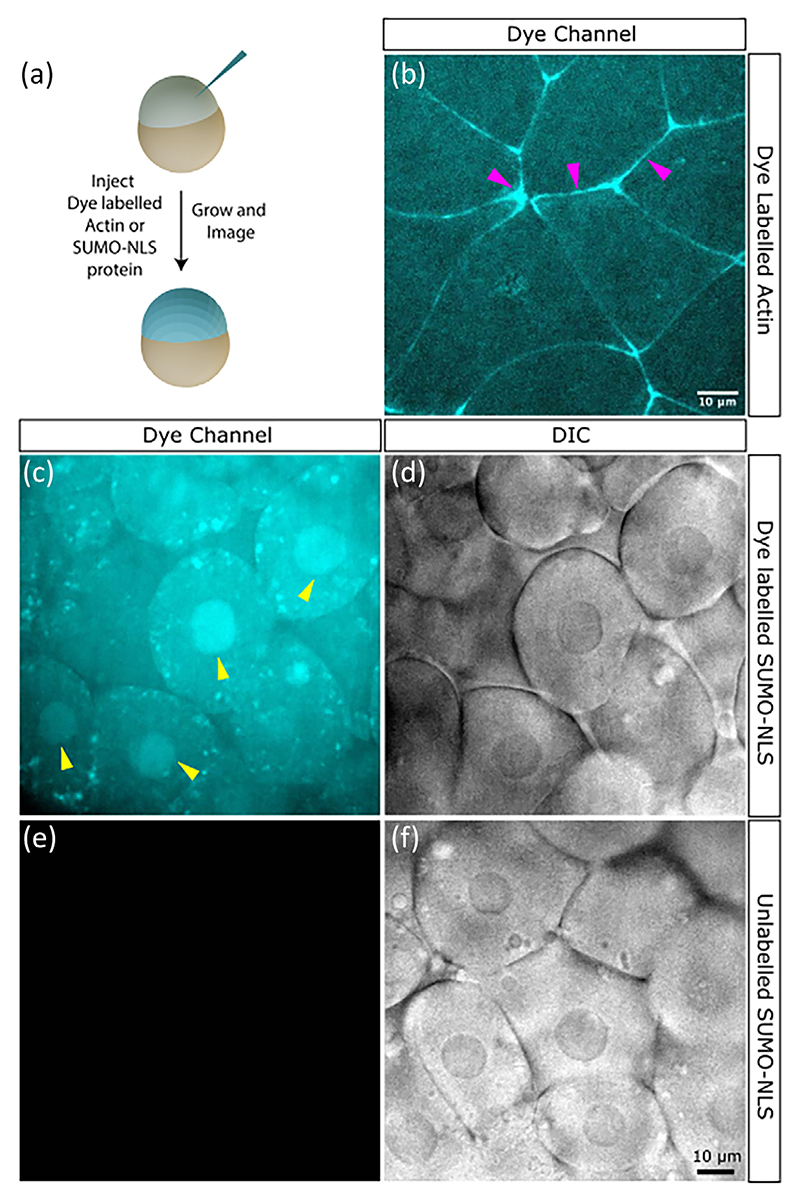
Detection of compound **F2** labelled proteins in zebrafish embryos. a) Schematic of the experimental paradigm used to test the detectability of compound **F2** labelled proteins in vivo. b) Zebrafish embryos were injected with compound **F2** dye labelled actin and imaged using an excitation wavelength of 405 nm and emission wavelength of 505 nm and above. Magenta arrowheads indicate staining observed at cellular junctions. c–f) Zebrafish embryos were injected with compound **F2** dye labelled (c and d) or unlabelled (e and f) SUMO-NLS protein. The dye channel (c and e) at the same settings for labelled and unlabelled protein and corresponding DIC channel (d and f) are shown. The yellow arrowheads in c show the location of nuclear structures as seen in the DIC channel d. The scale bars in b and f are 10 μm.

As our in vitro experiments with labelled actin showed that compound **F2** was the best suited for imaging proteins and protein assemblies, we selected it for further in vivo testing. Zebrafish embryos were chosen for these experiments due to their optical transparency and ease of injection, which allowed for protein introduction and live visualization (Figure 4a).^[34]^ First, we injected embryos with actin protein labelled with **F2** (see Figure S3 in the Supporting Information) and observed fluorescence at cellular margins, likely corresponding to cell junctions (Figure 4b). To confirm that the observed protein localization was not an artefact of labelling, we tested a protein target to a different cellular location, namely the nucleus. We designed a construct containing a small ubiquitin-like modifier (SUMO) tag for purification, fused to two SV40 nuclear localization signals (NLS), with three cysteines engineered into this cysteine-light NLS sequence for maleimide coupling.^[35]^ After purifying and labelling this protein with **F2** (see Figure S3), we injected it into embryos. The dye-labelled SUMO-NLS protein displayed clear nuclear staining observed via differential interference contrast (DIC) imaging (Figure 4c,d). By contrast, embryos injected with unlabeled SUMO-NLS protein displayed minimal autofluorescence, undetectable at the imaging settings used for dye-labelled samples (Figure 4e,f). Taken together, these preliminary studies demonstrate that proteins can be effectively labelled with fluorophore **F2**, retaining both function and in vivo detectability.

## 3 Conclusion

In conclusion, we have designed and synthesized clickable single-benzene-based fluorophores, **2F-2CN-Cys** and **2F-2CN-(*β*-NH**_**2**_**Ala)**. By substituting the ring heteroatom from sulfur (dihydro[1,4]thiazine skeleton) to nitrogen (tetrahydroquinoxaline skeleton), we were able to improve the photophysical properties of the resulting SBBF dyes. The maleimide group enabled conjugation of the fluorophores to actin filaments via thiol-ene click reaction. Using these compounds, we successfully labelled and visualized actin both in vitro and in vivo assays. Actin and SUMO-NLS proteins labelled with the **2F-2CN-(*β*-NH**_**2**_**Ala)**-based fluorophore **F2** localized to their expected positions in zebrafish embryos in vivo. To the best of our knowledge, this is the first example of single benzene-based fluorophores coupled to a protein for bioimaging. Studies on the incorporation of the parent fluorophore amino acids **2F-2CN-Cys** and **2F-2CN-(*β*-NH**_**2**_**Ala)** into proteins through genetic code expansion are ongoing in our laboratory.^[36]^

## Supplementary Material

The authors have cited additional references within the Supporting Information.[37–41]

Supporting Information

## Figures and Tables

**Table 1:** Photophysical properties of fluorophore derivatives **F1**−**F6** in EtOH.

Fluorophore	Abs λ_max,abs_ (nm)	Em λ_max,em_ (nm)	Molar Extinction Coefficient *ε* (L mol^−1^ cm^−1^)	Quantum Yield Φ_F_^a)^
**F1**	411	480	11,305	0.066
**F2**	415	488	7519	0.086
**F3**	410	480	6280	0.079
**F4**	415	490	5300	0.187
**F5**	411	481	6169	0.230
**F6**	414	488	5391	0.513

a) Calculated with respect to coumarin 6 in ethanol (Φ_F_ = 0.78) as standard.^[27]^

**Table 2:** Photophysical properties of fluorophore derivatives F1−F6 in water.

Fluorophore	Abs λ_max,abs_(nm)	Em λ_max,em_(nm)	Molar Extinction Coefficient *ε* (L mol ^−1^ cm ^−1^)	Quantum Yield Φ_F_^a)^
**F1**	410	488	5568	0.060
**F2**	410	492	2494	0.076
**F3**	410	490	2731	0.109
**F4**	412	491	3110	0.150
**F5**	413	490	5112	0.166
**F6**	407	490	1956	0.169

a) Calculated with respect to coumarin 6 in ethanol (Φ_F_ = 0.78) as standard applying the refraction indexes of water and EtOH.^[27]^

## Data Availability

The data that support the findings of this study are available in the supplementary material of this article.
