# Supplementary material for: Single-Benzene-Based Clickable Fluorophores for In Vitro and In Vivo Bioimaging
Source: ChemistrySelect. Author manuscript; Available in PMC 2025 Jun 23. (PMC7617796; doi:10.1002/slct.202405738)
Supplement: Supporting Information [file EMS206536-supplement-Supporting_Information.pdf]

Single-benzene-based clickable fluorophores for *in vitro* and *in vivo* bioimaging

Raja Mohanrao,[a,b] Clyde S. Pinto,[a] Andrejus Suchenko,[a] Guy J. Clarkson,[b] Martin Wills,[b] Stefan Roesner,*[b,c] Michael Shipman,*[b,d] and Mohan K. Balasubramanian*[a]

[a] *Centre for Mechanochemical Cell Biology and Warwick Medical School, Division of Biomedical Science, University of Warwick, Coventry, CV4 7AL, UK.*

[b] *Department of Chemistry, University of Warwick, Gibbet Hill Road, Coventry, CV4 7AL, UK.*

[c] *School of Pharmacy and Biomolecular Sciences, Liverpool John Moores University, Byrom Street, Liverpool L3 3AF, UK.*

[d] *The Palatine Centre, Stockton Road, Durham, DH1 3LE, UK.*

E-mail: s.k.roesner@ljmu.ac.uk, michael.shipman@durham.ac.uk, m.k.balasubramanian@warwick.ac.uk

Supporting Information

Table of Contents

1. Materials and methods S2

2. Detailed procedures and analytical data S3

3. UV/vis and fluorescence spectroscopy S13

4. Actin protein labelling S15

5. Depiction of single crystal X-ray structures S18

6. Copies of ^{1}H, ^{13}C and ^{19}F NMR spectra S22

7. References S43

1. Materials and methods

All chemicals and solvents were purchased from Sigma-Aldrich, Fisher Scientific, Fluorochem Ltd. and used without further purification. Reactions were monitored by thin layer chromatography (TLC) using Merck pre-coated silica plates (Silica Gel 60 F_{254}, 0.25 mm). TLC plates were visualized under ultraviolet light at 254 nm and also by charring using $KMnO_4$ solution. Column chromatography was performed on silica gel (200–400 mesh). Nuclear magnetic resonance (NMR) spectra were recorded on Bruker DPX 400 MHz or AV 500 MHz spectrometers. The residual solvent peaks were used as internal references and expressed in parts per million (ppm). Spin multiplicities were represented as s (singlet), d (doublet), t (triplet), quartet (q), dd (doublet of doublet), m (multiplet) and broad singlet (br. s). Coupling constants (*J*) are given in Hertz. Electrospray Ionisation (ESI) spectra were obtained using a Bruker MicroToF. High-resolution mass spectra were recorded using a Bruker MaXis Impact. UV-vis spectroscopy was carried out on an Agilent Cary 60 UV-vis Spectrophotometer. Fluorescence spectra were recorded on an Agilent Technologies Cary Eclipse Fluorescence Spectrophotometer. IR spectra were recorded using a Perkin Elmer Spectrum 100 FT-IR spectrometer and are given in cm^{-1}. Melting points were determined by using Gallenkamp MPD350 apparatus and were uncorrected. Specific rotations were recorded on an AA-1000 polarimeter using a 20 cm cuvette.

2. Detailed procedures and analytical data

tert-Butyl (*R*)-(2-(5,8-dicyano-6,7-difluoro-3,4-dihydro-2*H*-benzo[*b*][1,4]thiazine-3-carboxamido)ethyl)carbamate (11)

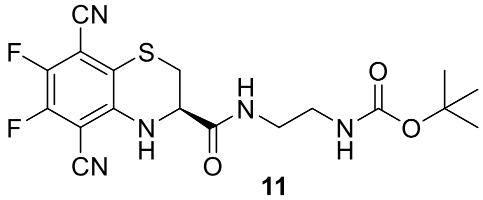

11

To a stirred solution of (*R*)-5,8-dicyano-6,7-difluoro-3,4-dihydro-2*H*-benzo[*b*][1,4]thiazine-3-carboxylic acid (**1**)[18] (0.22 g, 0.78 mmol) and HATU (0.45 g, 1.17 mmol) in anhydrous DMF (10 mL), a solution of *tert*-butyl (2-aminoethyl)carbamate (**2**)[34] (0.13 g, 1.17 mmol) and DIPEA (0.41 mmol, 2.34 mmol) in anhydrous DMF (5.0 mL) was added dropwise at 0 °C and stirring was continued for 12 h at rt. After completion of the reaction, the reaction mixture was extracted with ethyl acetate (50 mL), washed with saturated $NaHCO_3$ solution (2 x 25 mL), water (2 x 25 mL) and brine (25 mL). The organic phase was dried over anhydrous $MgSO_4$ and concentrated under reduced pressure. The crude residue was purified by column chromatography using ethyl acetate and petroleum ether (7:3, v/v) as eluent. The title compound **11** (0.17 g, 0.40 mmol, 51%) was obtained as a yellow solid.

R$_f$ (EtOAc/petroleum ether 7:3) 0.2;

mp 193–195 °C;

^{1}H NMR (500 MHz, DMSO-*d$_6$*) δ_H 8.13 (t, *J* = 5.5 Hz, 1H), 7.58 (d, *J* = 5.0 Hz, 1H), 6.77 (t, *J* = 5.4 Hz, 1H), 4.47 (dd, *J* = 8.4, 3.6 Hz, 1H), 3.44 (dd, *J* = 13.0, 3.5 Hz, 1H), 3.22–3.02 (m, 2H), 2.99 (m, 3H), 1.37 (s, 9H);

^{13}C NMR (126 MHz, DMSO-*d$_6$*) δ_C 169.9, 156.2, 147.9 (dd, *J* = 252, 13.6 Hz), 141.8, 141.3 (dd, *J* = 242, 14.2 Hz), 118.8 (d, *J* = 3.4 Hz), 111.9, 111.4 (d, *J* = 4.3 Hz), 104.6 (d, *J* = 1 4.1 Hz), 89.9 (d, *J* = 15.6 Hz), 78.2, 54.1, 28.6, 25.4;

^{19}F NMR (376 MHz, DMSO-*d$_6$*) δ_F –135.5 (d, *J* = 22.9 Hz), –148.9 (d, *J* = 22.6 Hz);

***ν*$_{max}$** (neat) = 3340 (NH), 3286 (NH), 2986, 2231 (CN), 1691 (C=O), 1669, 1526, 1474, 1250 cm^{-1};

MS (ESI$^-$) *m/z* 422.1 [M–H]$^-$;

HRMS (ESI/Q-TOF [M–H]$^-$ calcd. for $C_{18}H_{18}F_2N_5O_3S$ 422.1098, found 422.1104;

[α]$_D$^{28} –191 (*c* 0.0275, CH_3OH).

(*R*)-*N*-(2-Aminoethyl)-5,8-dicyano-6,7-difluoro-3,4-dihydro-2*H*-benzo[*b*][1,4]thiazine-3-carboxamide hydrochloride (12)

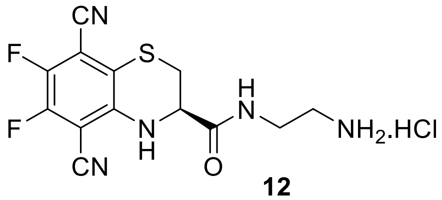

12

tert-Butyl (*R*)-(2-(5,8-dicyano-6,7-difluoro-3,4-dihydro-2*H*-benzo[*b*][1,4]thiazine-3-carboxamido)ethyl)carbamate (**11**) (0.15 g, 0.35 mmol) was dissolved in 4N HCl in 1,4-dioxane (5.0 mL) at 0 °C. Then, this mixture was stirred at rt for 12 h and concentrated under reduced pressure to give (*R*)-*N*-(2-aminoethyl)-5,8-dicyano-6,7-difluoro-3,4-dihydro-2*H*-benzo[*b*][1,4]thiazine-3-carboxamide hydrochloride (**12**) (0.10 g, 0.28 mmol, 80%) as a yellow solid.

R$_f$ (EtOAc/MeOH 4:1) 0.1;

mp 253–255 °C;

^{1}H NMR (400 MHz, DMSO-*d$_6$*) δ$_H$ 8.44 (s, 1H), 8.05 (s, 3H), 7.56 (d, *J* = 4.8 Hz, 1H), 4.54 (d, *J* = 4.0 Hz, 1H), 3.51 (dd, *J* = 13.0, 3.2 Hz, 1 H), 3.42 (td, *J* = 12.5, 5.9 Hz, 1 H), 3.35–3.22 (m, 1H), 3.04 (dd, *J* = 13.1, 3.2 Hz, 1H), 2.85 (d, *J* = 5.7 Hz, 2H);

^{13}C NMR (126 MHz, DMSO-*d$_6$*) δ 170.5, 147.9 (dd, *J* = 252, 13.8 Hz), 141.6 (dd, *J* = 243, 13.6 Hz), 118.8 (d, *J* = 3.7 Hz), 111.6 (d, *J* = 4.1 Hz), 104.7 (d, *J* = 2.5 Hz), 104.6 (d, *J* = 2.5 Hz), 90.0, 89.9, 54.2, 38.8, 37.1, 25.5;

^{19}F NMR (376 MHz, DMSO-*d$_6$*) δ$_F$ –135.3 (d, *J* = 22.8 Hz), –148.6 (d, *J* = 23.0 Hz);

***ν*$_{max}$** (neat) = 3377 (NH), 2916, 2230 (CN), 1664 (C=O), 1613, 1513, 1468, 1228 cm^{-1};

HRMS (ESI/Q-TOF) [M+H]$^+$ calcd. for $C_{13}H_{12}F_2N_5OS$ 324.0725, found 324.0724;

[α]$_D$^{28} –195.0 (*c* 0.02, CH_3OH).

(*R*)-5,8-Dicyano-*N*-(2-(2,5-dioxo-2,5-dihydro-1*H*-pyrrol-1-yl)ethyl)-6,7-difluoro-3,4-dihydro-2*H*-benzo[*b*][1,4]thiazine-3-carboxamide (F1)

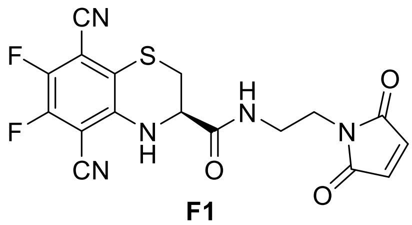

F1

Maleic anhydride (19 mg, 0.19 mmol) and potassium acetate (27 mg, 0.28 mmol) were dissolved in acetic acid (10 mL). To this solution, (*R*)-*N*-(2-aminoethyl)-5,8-dicyano-6,7-difluoro-3,4-dihydro-2*H*-benzo[*b*][1,4]thiazine-3-carboxamide hydrochloride (**12**) (0.10 g, 0.28 mmol) was added and the mixture was heated under reflux for 12 h. After completion of the reaction, the mixture was cooled to room temperature and poured slowly into chilled sodium carbonate solution. The mixture was extracted with ethyl acetate (2 x 25 mL), washed with water (25 mL) and brine (25 mL). The organic phase was dried over anhydrous $MgSO_4$ and then concentrated under reduced pressure. The crude residue was purified by column chromatography using ethyl acetate and petroleum ether (7:3, v/v) as eluent. The title compound (**F1**) (55 mg, 0.14 mmol, 50%) was obtained as a yellow solid.

R$_f$ (EtOAc) 0.4;

mp 196–198 °C;

^{1}H NMR (500 MHz, DMSO-*d$_6$*) δ$_H$ 8.15 (t, *J* = 6.0 Hz, 1H), 7.54 (d, *J* = 5.3 Hz, 1H), 6.96 (s, 2H), 4.42 (dd, *J* = 8.6, 3.5 Hz, 1H), 3.52–3.45 (m, 1H), 3.45–3.38 (m, 1H), 3.36 (m, 2H), 3.23 (dq, *J* = 11.7, 5.7 Hz, 1H), 2.94 (dd, *J* = 12.9, 3.4 Hz, 1H);

^{13}C NMR (126 MHz, DMSO-*d$_6$*) δ$_C$ 171.4, 169.9, 147.8 (dd, *J* = 251, 13.7 Hz), 141.5, 141.1 (dd, *J* = 243, 13.6 Hz), 134.9, 118.8 (d, *J* = 3.3 Hz), 111.8, 111.4 (d, *J* = 4.2 Hz), 104.6 (d, *J* = 14.0 Hz), 89.9 (d, *J* = 15.6 Hz), 53.8, 37.5, 37.4, 25.2;

^{19}F NMR (376 MHz, DMSO-*d$_6$*) δ$_F$ –135.7 (d, *J* = 22.3 Hz), –148.9 (d, *J* = 22.0 Hz);

***ν*$_{max}$** (neat) = 3342 (NH), 2944, 2231 (CN), 1698 (C=O), 1655, 1468, 1227 cm^{-1};

MS (ESI$^-$) *m/z* 402.0 [M–H]$^-$;

HRMS (ESI/Q-TOF) [M+Na]$^+$ calcd. for $C_{17}H_{11}F_2N_5NaO_3S$ 426.0449, found 426.0443;

[α]$_D$^{28} –49.3 (*c* 0.0375, CH_3OH).

Methyl 5,8-dicyano-6,7-difluoro-1,2,3,4-tetrahydroquinoxaline-2-carboxylate (9)

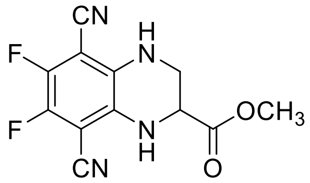

9

To a stirred solution of DL-2,3-diaminopropionic acid monohydrochloride (1.00 g, 7.11 mmol) in methanol (20 mL), thionyl chloride (2.58 mL, 35.6 mmol) was added dropwise over 10 min at 0 °C. After stirring for 30 min, the reaction mixture was heated under reflux for 10 h and then methanol was removed under reduced pressure. The residue was dissolved in DMF (20 mL). To this solution, tetrafluoroterephthalonitrile (**3**) (0.85 g, 4.27 mmol) and Et_3N (4.95 mL, 35.6 mmol) were added and stirring was continued for 12 h at rt. After completion of the reaction, the mixture was extracted with ethyl acetate (75 mL), washed with saturated $NaHCO_3$ solution (1 x 30 mL), water (2 x 30 mL) and brine (1 x 25 mL). The organic phase was dried over anhydrous $MgSO_4$ and then concentrated under reduced pressure. The crude residue was purified by column chromatography using ethyl acetate and petroleum ether (2:3, v/v) as eluent. The title compound methyl-5,8-dicyano-6,7-difluoro-1,2,3,4-tetrahydroquinoxaline-2-carboxylate (**9**) (0.93 g, 3.33 mmol, 78%) was obtained as a yellow solid.

R_f (EtOAc) 0.7;

mp 185–187 °C;

^{1}H NMR (500 MHz, DMSO-d_6) δ_H 7.42 (d, J = 3.4 Hz, 1H), 7.18 (d, J = 3.6 Hz, 1H), 4.34 (d, J = 2.4 Hz, 1H), 3.68 (s, 3H), 3.57 (d, J = 12.4 Hz, 1H), 3.43 (dd, J = 12.5, 3.9 Hz, 1H);

^{13}C NMR (126 MHz, DMSO-d_6) δ_C 171.5, 139.0 (dd, J = 248, 23.7 Hz), 138.8 (dd, J = 238, 11.1 Hz), 135.8, 135.5, 112.4 (t, J = 3.6 Hz), 86.0 (dd, J = 10.6, 1.6 Hz), 85.8 (dd, J = 10.1, 1.6 Hz), 52.9, 50.9, 41.4;

^{19}F NMR (376 MHz, DMSO-d_6) δ_F –151.5 (d, J = 22.0 Hz), –151.8 (d, J = 22.2 Hz);

ν_{max} (neat) = 3330 (NH), 2227 (CN), 1742 (C=O), 1646, 1529, 1485, 1293 cm^{-1};

MS (ESI^-) m/z 277.1 $[M–H]^-$;

HRMS (ESI/Q-TOF) $[M+Na]^+$ calcd. for $C_{12}H_8F_2N_4NaO_2$ 301.0513, found 301.0508.

5,8-Dicyano-6,7-difluoro-1,2,3,4-tetrahydroquinoxaline-2-carboxylic acid (5)

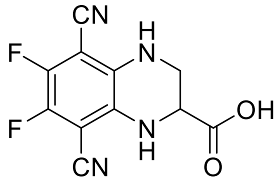

5

To a stirred solution of methyl-5,8-dicyano-6,7-difluoro-1,2,3,4-tetrahydroquinoxaline-2-carboxylate (**9**) (0.60 g, 2.14 mmol) in methanol (10 mL), K_2CO_3 (444 mg, 3.20 mmol) was added and the reaction mixture was heated under reflux for 12 h. After completion of the reaction, methanol was removed under reduced pressure. The residue was dissolved in water (25 mL), the pH was adjusted to 4 by the addition of 6 N HCl and the compound was precipitated from the solution. To this mixture, ethyl acetate (100 mL) was added to extract the compound into the organic phase. The organic phase was washed with water (2 x 25 mL) and brine (25 mL), dried over anhydrous $MgSO_4$ and concentrated under reduced pressure. The title compound 5,8-dicyano-6,7-difluoro-1,2,3,4-tetrahydroquinoxaline-2-carboxylic acid (**5**) (0.51 g, 1.95 mmol, 91%) was obtained as a yellow solid and used for the next step without further purification.

R_f (EtOAc/MeOH 4:1) 0.1;

mp 217–219 °C;

^{1}H NMR (500 MHz, DMSO-*d$_6$*) δ_H 13.03 (s, 1H), 7.31 (d, *J* = 3.1 Hz, 1H), 7.21 (d, *J* = 3.2 Hz, 1H), 4.20 (d, *J* = 2.1 Hz, 1H), 3.58 (d, *J* = 12.0 Hz, 1H), 3.42 (dd, *J* = 12.3, 3.8 Hz, 1H);

^{13}C NMR (126 MHz, DMSO-*d$_6$*) δ_C 172.5, 139.7 (dd, *J* = 22.6, 13.5 Hz), 137.8 (t, *J* = 12.9 Hz), 136.0, 135.9, 112.5, 85.5 (dd, *J* = 17.1, 1.4 Hz), 85.3 (dd, *J* = 17.1, 1.5 Hz), 50.9, 41.4;

^{19}F NMR (376 MHz, DMSO-*d$_6$*) δ_F –152.0 (d, *J* = 21.8 Hz), –152.2 (d, *J* = 21.8 Hz);

ν_{max} (neat) = 3341 (COOH), 2229 (CN), 1716 (C=O), 1505, 1235 cm^{-1};

MS (ESI$^-$) *m/z* 263.0 [M–H]$^-$;

HRMS (ESI/Q-TOF) [M–H]$^-$ calcd. for $C_{11}H_5F_2N_4O_2$ 263.0381, found 263.0386.

tert-Butyl (2-(5,8-dicyano-6,7-difluoro-1,2,3,4-tetrahydroquinoxaline-2-carboxamido) ethyl)carbamate (13)

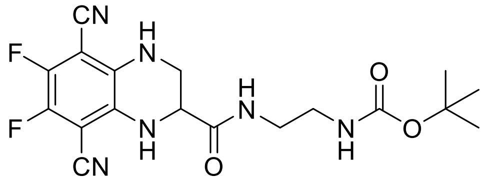

13

To a stirred solution of 5,8-dicyano-6,7-difluoro-1,2,3,4-tetrahydroquinoxaline-2-carboxylic acid (**5**) (0.20 g, 0.76 mmol) and HATU (0.43 g, 1.14 mmol) in anhydrous DMF (10 mL), a solution of *tert*-butyl (2-aminoethyl)carbamate[34] (121 mg, 0.76 mmol) and DIPEA (0.40 mL, 2.27 mmol) in anhydrous DMF (5.0 mL) was added dropwise at 0 °C and stirring was continued for 12 h at rt. After completion of the reaction, the mixture was extracted with ethyl acetate (50 mL), washed with saturated $NaHCO_3$ solution (2 x 25 mL), water (2 x 25 mL) and brine (25 mL). The organic phase was dried over anhydrous $MgSO_4$ and concentrated under reduced pressure. The crude residue was purified by column chromatography using ethyl acetate and petroleum ether (7:3, v/v) as eluent. The title compound *tert*-butyl (2-(5,8-dicyano-6,7-difluoro-1,2,3,4-tetrahydroquinoxaline-2-carboxamido)ethyl)carbamate (**13**) (0.13 g, 0.32 mmol, 42%) was obtained as a yellow solid.

R_f (EtOAc) 0.25;

mp 243 °C;

^{1}H NMR (500 MHz, DMSO-*d$_6$*) δ_H 8.06 (t, *J* = 5.6 Hz, 1H), 7.27 (d, *J* = 3.4 Hz, 1H), 7.11 (d, *J* = 2.1 Hz, 1H), 6.79 (t, *J* = 5.2 Hz, 1H), 4.08–4.03 (m, 1H), 3.50 (d, *J* = 12.0 Hz, 1H), 3.29 (dd, *J* = 11.9, 3.1 Hz, 1H), 3.19–3.01 (m, 2H), 3.01–2.92 (m, 2H), 1.38 (s, 9H);

^{13}C NMR (126 MHz, DMSO-*d$_6$*) δ_C 170.4 (d, *J* = 4.3 Hz), 156.1, 139.0 (dd, *J* = 241, 17.4 Hz), 138.3 (dd, *J* = 232, 8.7 Hz), 136.2, 135.9, 112.7 (dd, *J* = 24.0, 3.1 Hz), 85.6 (dd, *J* = 49.1, 16.9 Hz), 78.2, 52.2, 41.6, 28.7;

^{19}F NMR (376 MHz, DMSO-*d$_6$*) δ_F –152.4 (d, *J* = 21.9 Hz), –152.7 (d, *J* = 21.8 Hz);

ν_{max} (neat) = 3332 (NH), 2982, 2226 (CN), 1684 (C=O), 1670, 1527, 1490, 1201 cm^{-1};

MS (ESI$^-$) *m/z* 405.1 [M–H]$^-$;

HRMS (ESI/Q-TOF) [M–H]$^-$ calcd. for $C_{18}H_{19}F_2N_6O_3$ 405.1487, found 405.1492.

N-(2-Aminoethyl)-5,8-dicyano-6,7-difluoro-1,2,3,4-tetrahydroquinoxaline-2-carboxamide hydrochloride (14)

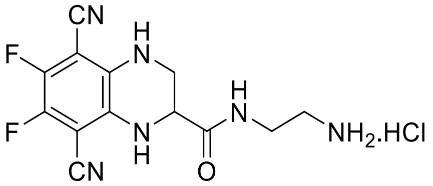

14

tert-Butyl (2-(5,8-dicyano-6,7-difluoro-1,2,3,4-tetrahydroquinoxaline-2-carboxamido)ethyl)carbamate (**13**) (0.10 g, 0.25 mmol) was dissolved in 4N HCl in 1,4-dioxane (5.0 mL) at 0 °C. The mixture was stirred at rt for 12 h and then concentrated under reduced pressure to give *N*-(2-aminoethyl)-5,8-dicyano-6,7-difluoro-1,2,3,4-tetrahydroquinoxaline-2-carboxamide hydrochloride (**14**) (75 mg, 0.22 mmol, 86%) as a yellow solid.

$\mathbf{R_f}$ (EtOAc) 0.3;

mp 189–191 °C;

^{1}H NMR (500 MHz, DMSO-d_6) δ_H 8.47 (d, J = 5.3 Hz, 1H), 8.10 (br. s, 3H), 7.23 (d, J = 3.3 Hz, 1H), 7.15 (d, J = 2.2 Hz, 1H), 4.13 (d, J = 3.0 Hz, 1H), 3.55 (d, J = 12.2 Hz, 1H), 3.42–3.23 (m, 3H), 2.86 (t, J = 6.3 Hz, 2H);

^{13}C NMR (126 MHz, DMSO-d_6) δ_C 171.0, 139.9–139.4 (m), 137.8 (t, J = 15.3 Hz), 136.2, 136.0, 112.7 (d, J = 2.9 Hz), 112.6 (d, J = 2.7 Hz), 85.8 (d, J = 16.6 Hz), 85.5 (d, J = 16.2 Hz), 52.2, 41.6, 38.9, 37.0;

^{19}F NMR (376 MHz, DMSO-d_6) δ_F –152.3 (d, J = 21.8 Hz), –152.4 (d, J = 21.8 Hz);

$\boldsymbol{\nu_{max}}$ (neat) = 3229 (NH), 2981, 2224 (CN), 1666 (C=O), 1645, 1514, 1480, 1235 cm^{-1};

MS (ESI$^-$) *m/z* 305.1 [M–H]$^-$;

HRMS (ESI/Q-TOF) [M–H]$^-$ calcd. for $C_{13}H_{11}F_2N_6O$ 305.0962, found 305.0968.

5,8-Dicyano-N-(2-(2,5-dioxo-2,5-dihydro-1H-pyrrol-1-yl)ethyl)-6,7-difluoro-1,2,3,4-tetrahydroquinoxaline-2-carboxamide (F2)

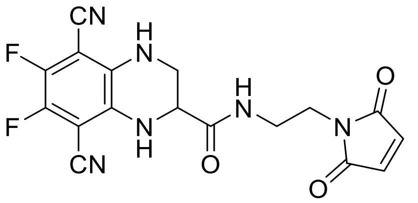

F2

Maleic anhydride (17 mg, 0.17 mmol) and potassium acetate (25 mg, 0.25 mmol) were dissolved in acetic acid (10 mL). To this solution, *N*-(2-aminoethyl)-5,8-dicyano-6,7-difluoro-1,2,3,4-tetrahydroquinoxaline-2-carboxamide hydrochloride (**14**) (85 mg, 0.25 mmol) was added and the mixture was heated under reflux for 12 h. After completion of the reaction, the mixture was cooled to room temperature and poured slowly into a chilled solution of sodium carbonate. The residue was extracted with ethyl acetate (2 x 25 mL), washed with water (25 mL) and brine (25 mL). The organic phase was dried over anhydrous $MgSO_4$ and then concentrated under reduced pressure. The crude residue was purified by column chromatography using ethyl acetate and petroleum ether (7:3, v/v) as eluent. The title compound 5,8-dicyano-*N*-(2-(2,5-dioxo-2,5-dihydro-1*H*-pyrrol-1-yl)ethyl)-6,7-difluoro-1,2,3,4-tetrahydroquinoxaline-2-carboxamide (**F2**) (50 mg, 0.13 mmol, 51%) was obtained as a yellow solid.

$\mathbf{R_f}$ (EtOAc) 0.3;

mp 242–244 °C;

^{1}H NMR (400 MHz, DMSO-*d$_6$*) δ_H 8.10 (t, *J* = 5.9 Hz, 1H), 7.22 (d, *J* = 3.8 Hz, 1H), 7.06 (s, 1H), 6.96 (s, 2H), 3.98 (d, *J* = 3.7 Hz, 1H), 3.45 (t, *J* = 5.7 Hz, 3H), 3.25 (dd, *J* = 12.2, 6.8 Hz, 3H);

^{13}C NMR (126 MHz, DMSO-*d$_6$*) δ_C 171.4, 170.5, 139.7 (dd, *J* = 14.9, 6.2 Hz), 137.8 (dd, *J* = 16.1, 7.4 Hz), 136.2, 135.6, 134.9, 112.7, 112.6, 85.8 (d, *J* = 14.5 Hz), 85.5 (d, *J* = 15.9 Hz), 52.2, 41.4, 37.4, 37.4;

^{19}F NMR (376 MHz, DMSO-*d$_6$*) δ_F –152.6 (d, *J* = 21.7 Hz), –152.6 (d, *J* = 21.9 Hz);

ν_{max} (neat) = 3336 (NH), 3112, 2225 (CN), 1704 (C=O), 1670, 1572, 1490, 1253 cm^{-1};

MS (ESI$^-$) *m/z* 385.1 [M–H]$^-$;

HRMS (ESI/Q-TOF) [M+Na]$^+$ calcd. for $C_{17}H_{12}F_2N_6NaO_3$ 409.0837, found 409.0831.

(*R*)-*N*-(2-Azidoethyl)-5,8-dicyano-6,7-difluoro-3,4-dihydro-2*H*-benzo[*b*][1,4]thiazine-3-carboxamide (6)

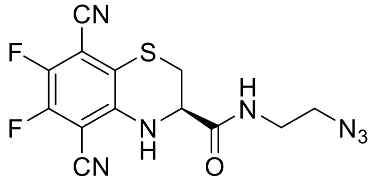

6

To a solution of (*R*)-5,8-dicyano-6,7-difluoro-3,4-dihydro-2*H*-benzo[*b*][1,4]thiazine-3-carboxylic acid[18] (0.10 g, 0.36 mmol) and HATU (176 mg, 0.46 mmol) in anhydrous DMF (5.0 mL), a solution of 2-azidoethan-1-amine[35] (37 mg, 0.43 mmol) and DIPEA (81 µL, 0.46 mmol) in anhydrous DMF (5.0 mL) was added dropwise at 0 °C and stirring was continued for 12 h at rt. After completion of the reaction, the mixture was diluted with ethyl acetate (50 mL), washed with saturated $NaHCO_3$ solution (1 x 30 mL), water (2 x 30 mL) and brine (1 x 25 mL). The organic phase was dried over anhydrous $MgSO_4$ and then concentrated under reduced pressure. The crude residue was purified by column chromatography using ethyl acetate and petroleum ether (4:1, v/v) as eluent. The title compound (*R*)-*N*-(2-azidoethyl)-5,8-dicyano-6,7-difluoro-3,4-dihydro-2*H*-benzo[*b*][1,4]thiazine-3-carboxamide (**6**) (50 mg, 0.14 mmol, 40%) was obtained as a yellow solid.

R_f (EtOAc) 0.3;

mp 181–183 °C;

^{1}H NMR (500 MHz, DMSO-*d$_6$*) δ_H 8.33 (d, *J* = 5.6 Hz, 1H), 7.64 (d, *J* = 5.3 Hz, 1H), 4.51 (dt, *J* = 5.2, 3.4 Hz, 1H), 3.46 (dd, *J* = 13.0, 3.3 Hz, 1H), 3.39–3.29 (m, 3H), 3.28–3.19 (m, 1H), 2.98 (dd, *J* = 13.0, 3.4 Hz, 1H);

^{13}C NMR (126 MHz, DMSO-*d$_6$*) δ_C 170.2, 147.8 (dd, *J* = 251, 13.6 Hz), 141.7, 140.0 (dd, *J* = 245, 13.7 Hz), 118.7 (d, *J* = 3.3 Hz), 111.9, 111.4 (d, *J* = 4.4 Hz), 104.6 (d, *J* = 14.3 Hz), 90.0 (d, *J* = 15.6 Hz), 54.0, 50.4, 39.2, 25.3;

^{19}F NMR (376 MHz, DMSO-*d$_6$*) δ_F –135.5 (d, *J* = 23.1 Hz), –148.9 (d, *J* = 22.3 Hz);

ν_{max} (neat) = 3349 (NH), 3278 (NH), 3104, 2924, 2233 (CN), 2093 (N_3), 1663 (C=O), 1570, 1473, 1263, 1252 cm^{-1};

MS (ESI$^-$) *m/z* 348.0 [M–H]$^-$;

HRMS (ESI/Q-TOF) [M+Na]$^+$ calcd. for $C_{13}H_9F_2N_7NaOS$ 372.0455, found 372.0450;

$[\alpha]_D^{28}$ –126.0 (*c* 0.0125, CH_3OH).

(*R*)-5,8-Dicyano-*N*-(2-(4-((2,5-dioxo-2,5-dihydro-1*H*-pyrrol-1-yl)methyl)-1*H*-1,2,3-triazol-1-yl)ethyl)-6,7-difluoro-3,4-dihydro-2*H*-benzo[*b*][1,4]thiazine-3-carboxamide (F3)

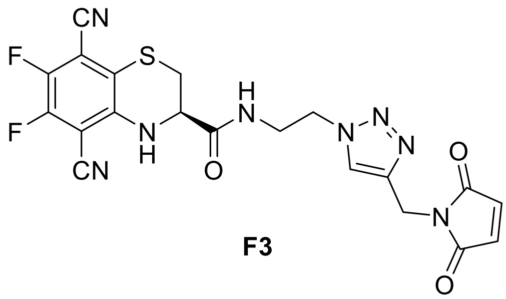

F3

To a solution of (*R*)-5,8-dicyano-*N*-(2-(2,5-dioxo-2,5-dihydro-1*H*-pyrrol-1-yl)ethyl)-6,7-difluoro-3,4-dihydro-2*H*-benzo[*b*][1,4]thiazine-3-carboxamide (**6**) (0.20 g, 0.57 mmol) and 1-(prop-2-yn-1-yl)-1*H*-pyrrole-2,5-dione (**8**) (68 mg, 0.50 mmol) in $CH_3CN:H_2O$ (2:1, 10 mL), CuI (54 mg, 0.28 mmol) and sodium ascorbate (56 mg, 0.28 mmol) were added and the mixture was heated to 80 °C for 12 h. After completion of the reaction, the mixture was extracted with ethyl acetate (50 mL), washed with saturated $NaHCO_3$ solution (2 x 25 mL), water (2 x 25 mL) and brine (25 mL). The organic phase was dried over anhydrous $MgSO_4$ and concentrated under reduced pressure. The crude residue was purified by column chromatography using ethyl acetate as eluent. The title compound (*R*)-5,8-dicyano-*N*-(2-(4-((2,5-dioxo-2,5-dihydro-1*H*-pyrrol-1-yl)methyl)-1*H*-1,2,3-triazol-1-yl)ethyl)-6,7-difluoro-3,4-dihydro-2*H*-benzo[*b*][1,4]thiazine-3-carboxamide (**F3**) (0.23 g, 0.48 mmol, 84%) was obtained as a yellow solid.

R_f (EtOAc/petroleum ether 8:2) 0.2;

mp 180–182 °C;

^{1}H NMR (400 MHz, DMSO-*d₆*) δ_H 8.24 (t, *J* = 5.3 Hz, 1H), 7.93 (s, 1H), 7.55 (d, *J* = 5.0 Hz, 1H), 7.08 (d, *J* = 8.7 Hz, 2H), 4.63 (s, 2H), 4.46 (dd, *J* = 8.0, 4.0 Hz, 1H), 4.38 (t, *J* = 5.5 Hz, 2H), 4.20 (d, *J* = 2.3 Hz, 1H), 3.51 (dd, *J* = 11.2, 5.4 Hz, 2H), 2.97 (dd, *J* = 13.0, 3.2 Hz, 1H);

^{13}C NMR (126 MHz, DMSO-d_6) δ_C 170.9, 170.2, 147.8 (dd, *J* = 252, 13.5 Hz), 142.5, 141.7, 141.1 (dd, *J* = 247, 14.0 Hz), 135.4, 135.2, 123.9, 118.7 (d, *J* = 3.5 Hz), 111.9 (d, *J* = 3.0 Hz), 111.4 (d, *J* = 4.2 Hz), 104.7 (d, *J* = 14.1 Hz), 89.8 (d, *J* = 15.5 Hz), 74.2, 53.8, 49.0, 32.9, 26.8, 25.3;

^{19}F NMR (376 MHz, DMSO-d_6) δ_F –135.4 (d, *J* = 22.6 Hz), –148.6 (d, *J* = 22.6 Hz);

ν_{max} (neat) = 3349 (NH), 2979, 2226 (CN), 1699 (C=O), 1593, 1515, 1397, 1245 cm^{-1};

MS (ESI^-) *m/z* 483.0 $[M–H]^-$;

HRMS (ESI/Q-TOF) $[M+Na]^+$ calcd. for $C_{20}H_{14}F_2N_8NaO_3S$ 507.0775, found 507.0770;

$[\alpha]_D{}^{28}$ –89.4 (*c* 0.033, CH_3OH).

***N*-(2-Azidoethyl)-5,8-dicyano-6,7-difluoro-1,2,3,4-tetrahydroquinoxaline-2-carboxamide (7)**

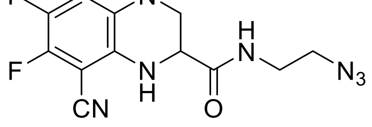

7

To a stirred solution of 5,8-dicyano-6,7-difluoro-1,2,3,4-tetrahydroquinoxaline-2-carboxylic acid (**5**) (0.10 g, 0.38 mmol) and HATU (0.19 g, 0.49 mmol) in anhydrous DMF (5.0 mL), a solution of 2-azidoethan-1-amine[35] (36 mg, 0.42 mmol) and DIPEA (0.20 mL, 1.14 mmol) in anhydrous DMF (5.0 mL) was added dropwise at 0 °C and stirring was continued for 12 h at rt. After completion of the reaction, the mixture was diluted with ethyl acetate (50 mL), washed with saturated $NaHCO_3$ solution (1 x 30 mL), water (2 x 30 mL) and brine (1 x 25

mL). The organic phase was dried over anhydrous $MgSO_4$ and then concentrated under reduced pressure. The crude residue was purified by column chromatography using ethyl acetate and petroleum ether (7:3, v/v) as eluent. The title compound *N*-(2-azidoethyl)-5,8-dicyano-6,7-difluoro-1,2,3,4-tetrahydroquinoxaline-2-carboxamide (**7**) (106 mg, 0.32 mmol, 84%) was obtained as a yellow solid.

R$_f$ (EtOAc/petroleum ether 4:1) 0.3;

mp 235–237 °C;

^{1}H NMR (500 MHz, DMSO-*d$_6$*) δ$_H$ 8.25 (t, *J* = 5.6 Hz, 1H), 7.32 (d, *J* = 3.9 Hz, 1H), 7.13 (d, *J* = 2.7 Hz, 1H), 4.10 (d, *J* = 2.9 Hz, 1H), 3.52 (d, *J* = 12.1 Hz, 1H), 3.36 (m, 2H), 3.33–3.22 (m, 3H);

^{13}C NMR (126 MHz, DMSO-*d$_6$*) δ$_C$ 170.8, 139.6 (dd, *J* = 30.2, 11.7 Hz), 137.7 (dd, J = 20.6 Hz, 18.8 Hz), 136.2, 135.9, 112.8, 112.6, 85.7 (d, *J* = 15.4 Hz), 85.3 (d, *J* = 16.5 Hz), 52.1, 50.4, 41.6, 39.0;

^{19}F NMR (376 MHz, DMSO-*d$_6$*) δ$_F$ –152.5 (d, *J* = 21.9 Hz), –152.7 (d, *J* = 21.8 Hz);

IR ν_{max} (neat) = 3337 (NH), 3097, 2224 (CN), 2103 (N_3) 1664 (C=O), 1538, 1489, 1305 cm^{-1};

MS (ESI$^-$) *m/z* 331.1 [M–H]$^-$;

HRMS (ESI/Q-TOF) [M–H]$^-$ calcd. for $C_{13}H_9F_2N_8O$ 331.0867, found 331.0873.

5,8-Dicyano-*N*-(2-(4-((2,5-dioxo-2,5-dihydro-1*H*-pyrrol-1-yl)methyl)-1*H*-1,2,3-triazol-1-yl)ethyl)-6,7-difluoro-1,2,3,4-tetrahydroquinoxaline-2-carboxamide (F4)

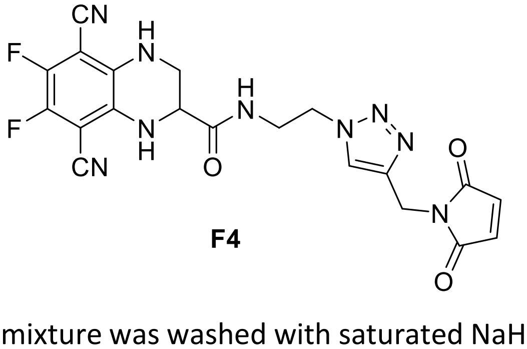

F4

To a solution of *N*-(2-azidoethyl)-5,8-dicyano-6,7-difluoro-1,2,3,4-tetrahydroquinoxaline-2-carboxamide (**7**) (0.20 g, 0.60 mmol) and 1-(prop-2-yn-1-yl)-1*H*-pyrrole-2,5-dione (**8**) (90 mg, 0.67 mmol) in CH_3CN:H_2O (2:1, 10 mL), $CuSO_4.5H_2O$ (0.15 g, 0.60 mmol) and sodium ascorbate (0.12 g, 0.60 mmol) were added and the mixture was heated to 80 °C for 12 h. The reaction mixture was cooled to rt and diluted with ethyl acetate (50 mL). The mixture was washed with saturated $NaHCO_3$ solution (2 x 25 mL), water (2 x 25 mL) and brine (25 mL). The organic phase was dried over anhydrous $MgSO_4$ and concentrated under reduced pressure. The crude residue was purified by column chromatography using ethyl acetate as eluent. The title compound 5,8-dicyano-*N*-(2-(4-((2,5-dioxo-2,5-dihydro-1*H*-pyrrol-1-yl)methyl)-1*H*-1,2,3-triazol-1-yl)ethyl)-6,7-difluoro-1,2,3,4-tetrahydroquinoxaline-2-carboxamide (**F4**) (0.24 g, 0.52 mmol, 87%) was obtained as a yellow solid.

R$_f$ (EtOAc) 0.15;

mp 135–137 °C;

^{1}H NMR (500 MHz, DMSO-*d$_6$*) δ$_H$ 8.19 (t, *J* = 5.6 Hz, 1H), 7.96 (s, 1H), 7.27 (d, *J* = 3.7 Hz, 1H), 7.08 (s, 3H), 4.66 (s, 2H), 4.38 (t, *J* = 6.0 Hz, 2H), 4.05 (d, *J* = 3.1 Hz, 1H), 3.51–3.43 (m, 3H), 3.28 (dd, *J* = 12.1, 3.4 Hz, 1H);

^{13}C NMR (126 MHz, DMSO-*d6*) δ_C 171.0, 170.8, 142.6, 138.8 (dd, *J* = 242, 16.5 Hz), 138.7 (dd, *J* = 247, 20.5 Hz), 136.1, 135.9, 135.2, 123.9, 112.7 (d, *J* = 2.9 Hz), 112.6 (d, *J* = 3.2 Hz), 85.8 (dd, *J* = 17.1, 1.5 Hz), 85.5 (dd, *J* = 17.1, 1.4 Hz), 52.0, 49.1, 41.6, 33.0;

^{19}F NMR (376 MHz, DMSO-*d6*) δ_F –152.3 (d, *J* = 21.8 Hz), –152.5 (d, *J* = 21.9 Hz);

***ν*max** (neat) = 3319 (NH), 3101, 2227 (CN), 1771 (C=O), 1703, 1669, 1567, 1521, 1323, 1228 cm^{-1};

MS (ESI$^-$) *m/z* 466.1 [M–H]$^-$;

HRMS (ESI/Q-TOF) [M+Na]$^+$ calcd. for $C_{20}H_{15}F_2N_9NaO_3$ 490.1164, found 490.1158.

Methyl *N*-(*tert*-butoxycarbonyl)-*S*-(1-(2-((*R*)-5,8-dicyano-6,7-difluoro-3,4-dihydro-2*H*-benzo[*b*][1,4]thiazine-3-carboxamido)ethyl)-2,5-dioxopyrrolidin-3-yl)-L-cysteinate (F5)

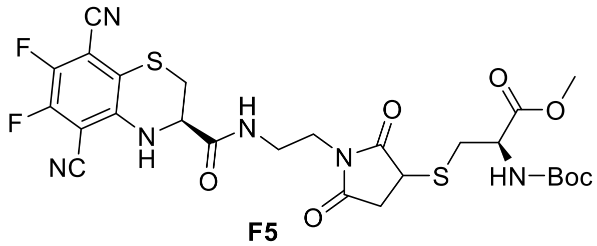

F5

To a solution of (*R*)-5,8-dicyano-*N*-(2-(2,5-dioxo-2,5-dihydro-1*H*-pyrrol-1-yl)ethyl)-6,7-difluoro-3,4-dihydro-2*H*-benzo[*b*][1,4]thiazine-3-carboxamide (**F1**) (60 mg, 0.15 mmol) and *N*-(*tert*-butoxycarbonyl)-L-cysteine methyl ester (**10**) (35 mg, 0.15 mmol) in anhydrous CH_2Cl_2 (10 mL), triethylamine (42 µL, 0.30 mmol) was added and the mixture was stirred at rt for 24 h. After completion of the reaction, the mixture was diluted with ethyl acetate (50 mL), washed with saturated $NaHCO_3$ solution (2 x 25 mL), water (2 x 25 mL) and brine (25 mL). The organic phase was dried over anhydrous $MgSO_4$ and then concentrated under reduced pressure. The crude residue was purified by column chromatography using ethyl acetate and petroleum ether (2:3, v/v) as eluent. **F5** was obtained as a mixture of diastereomers (51 mg, 0.08 mmol, 53%).

R_f (EtOAc/petroleum ether 3:2) 0.2;

mp 130–132 °C;

^{1}H NMR (400 MHz, DMSO-*d6*) δ_H 8.18 (dt, *J* = 13.2, 6.7 Hz, 1H), 7.52 (dd, *J* = 9.7, 5.1 Hz, 1H), 7.37 (d, *J* = 8.2 Hz, 1H), 4.43 (m, 1H), 4.23 (m, 1H), 3.97 (dd, *J* = 8.7, 4.0 Hz, 1H), 3.65 (s, 3H), 3.51–3.35 (m, 4H), 3.28–2.75 (m, 6H), 1.39 (s, 9H);

^{13}C NMR (126 MHz, DMSO-*d6*) δ_C 177.1 (d, *J* = 4.1 Hz), 175.4, 171.8 (d, *J* = 6.0 Hz), 170.0, 155.8 (d, *J* = 18.9 Hz), 148.8 (d, *J* = 13.8 Hz), 146.8 (d, *J* = 13.9 Hz), 142.1 (d, *J* = 14.1 Hz), 141.5 (d, *J* = 11.5 Hz), 140.1 (d, *J* = 14.0 Hz), 118.9, 111.8, 111.5, 104.6 (d, *J* = 12.9 Hz), 90.0 (d, *J* = 4.4 Hz), 89.9 (d, *J* = 4.5 Hz), 79.0, 54.0, 53.9, 53.3, 52.6 (d, *J* = 5.8 Hz), 38.5, 38.4, 36.7, 36.5, 36.2, 32.9, 32.6, 28.6, 25.2, 25.1;

^{19}F NMR (376 MHz, DMSO-*d6*) δ_F –135.6 (m), –148.8 (m);

***ν*max** (neat) = 3349 (NH), 2952, 2232 (CN), 1701 (C=O), 1471, 1397, 1164 cm^{-1};

MS (ESI$^+$) *m/z* 661.1 [M+Na]$^+$;

HRMS (ESI/Q-TOF) [M+Na]$^+$ calcd. for $C_{26}H_{28}F_2N_6NaO_7S_2$ 661.1327, found 661.1321.

Methyl *N*-(*tert*-butoxycarbonyl)-*S*-(1-(2-(5,8-dicyano-6,7-difluoro-1,2,3,4-tetrahydroquinoxaline-2-carboxamido)ethyl)-2,5-dioxopyrrolidin-3-yl)-L-cysteinate (F6)

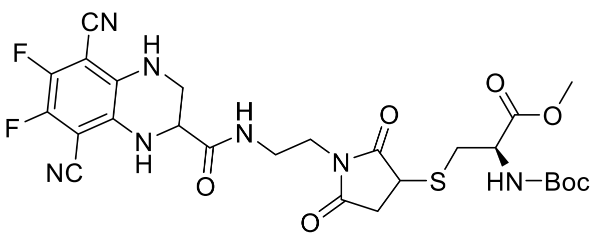

F6

To a solution of 5,8-dicyano-*N*-(2-(2,5-dioxo-2,5-dihydro-1*H*-pyrrol-1-yl)ethyl)-6,7-difluoro-1,2,3,4-tetrahydroquinoxaline-2-carboxamide (**F2**) (60 mg, 0.16 mmol) and *N*-(*tert*-butoxycarbonyl)-L-cysteine methyl ester (**10**) (37 mg, 0.16 mmol) in anhydrous CH_2Cl_2 (10 mL), triethylamine (44 µL, 0.32 mmol) was added and the reaction mixture was stirred for 12 h at rt. After completion of the reaction, the mixture was diluted with ethyl acetate (50 mL), washed with saturated $NaHCO_3$ solution (2 x 25 mL), water (2 x 25 mL) and brine (25 mL). The organic phase was dried over anhydrous $MgSO_4$ and then concentrated under reduced pressure. The crude residue was purified by column chromatography using ethyl acetate and petroleum ether (2:3, v/v) as eluent. **F6** was obtained as a mixture of diastereomers (62 mg, 0.10 mmol, 61%).

R_f (EtOAc/petroleum ether 3:2) 0.22;

mp 113–115 °C;

^{1}H NMR (400 MHz, DMSO-*d₆*) δ_H 8.12 (td, *J* = 9.9, 6.4 Hz, 1H), 7.41–7.33 (m, 1H), 7.25–7.16 (m, 1H), 7.08 (s, 1H), 4.31–4.17 (m, 1H), 4.03–3.91 (m, 2H), 3.65 (s, 3H), 3.52–3.40 (m, 3H), 3.30–3.18 (m, 4H), 3.15–2.96 (m, 2H), 2.86 (d, *J* = 10.4 Hz, 1H), 1.39 (s, 9H);

^{13}C NMR (126 MHz, DMSO-d_6) δ_C 177.1, 175.3, 171.8, 170.6, 155.8 (d, *J* = 18.5 Hz), 139.7 (dd, *J* = 16.0, 10.5 Hz), 137.8 (dd, *J* = 15.6, 11.2 Hz), 136.1, 135.5 (d, *J* = 9.1 Hz), 112.7, 112.6, 86.0, 85.8–85.4 (m), 79.0, 54.0, 53.4, 52.6, 52.5, 52.3, 52.2, 41.4, 41.3, 38.5, 36.6, 36.4, 36.1, 32.9, 32.6, 28.6;

^{19}F NMR (376 MHz, DMSO-d_6) δ_F –152.3 (m);

ν_{max} (neat) = 3349 (NH), 2979, 2226 (CN), 1699 (C=O), 1593, 1515, 1397, 1245 cm^{-1};

MS (ESI$^+$) *m/z* 644.2 $[M+Na]^+$;

HRMS (ESI/Q-TOF) $[M+Na]^+$ calcd. for $C_{26}H_{29}F_2N_7NaO_7S$ 644.1715, found 644.1709.

3. UV/vis and fluorescence spectroscopy

A solution of a fluorescent compound (**F1**–**F6**) was prepared in either EtOH or water to a given concentration (nM–mM). Prior to UV/vis spectroscopy, a blank was measured in EtOH or water, respectively. All measurements were carried out using a quartz cuvette (10 mm × 10 mm) filled to a total volume of 2 mL.

The data was normalised by dividing the UV absorption/fluorescence values at each wavelength by the maximal UV absorption/emission value for each set of data.

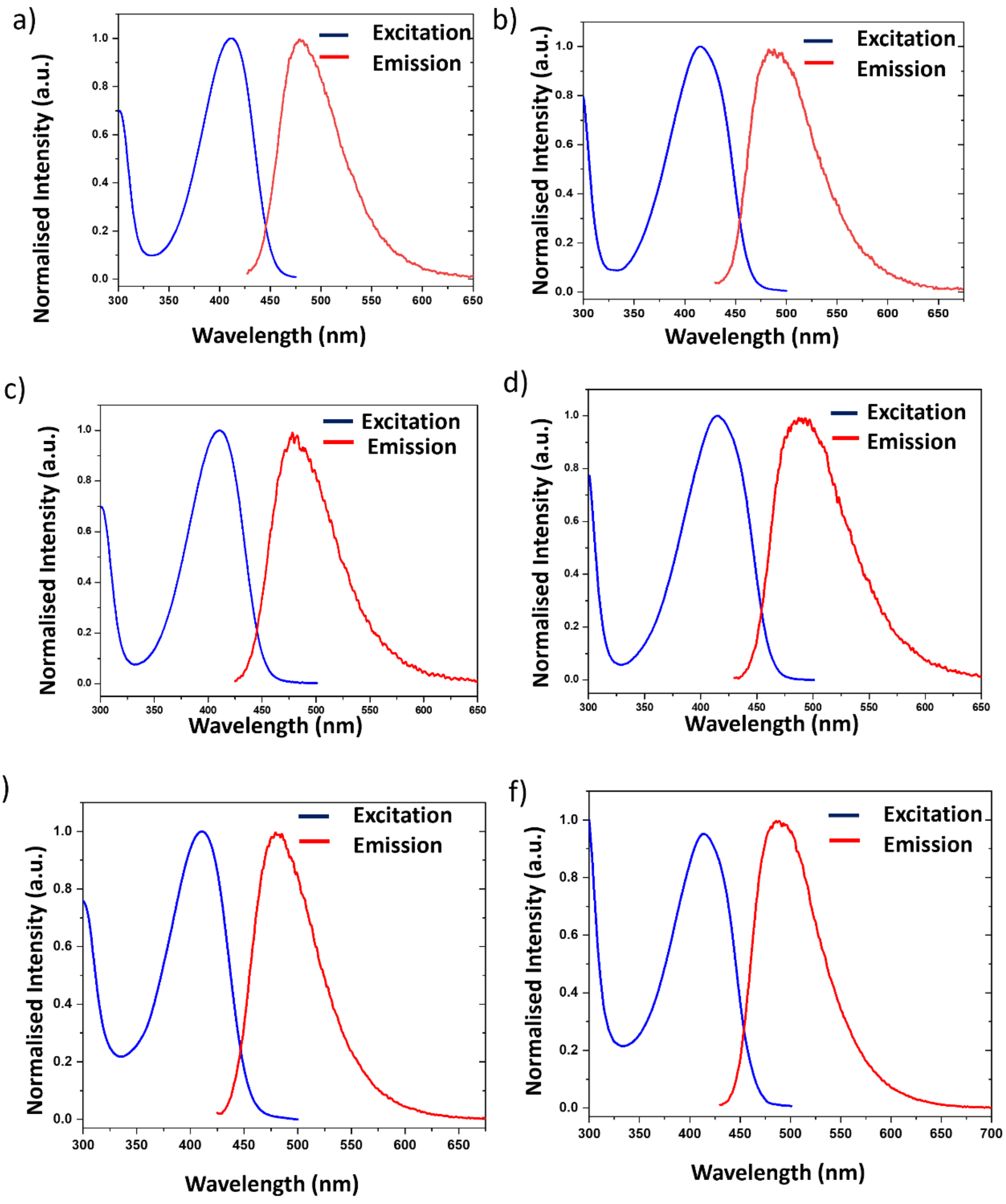

Figure S1. Excitation and emission spectra for compounds **F1** (a), **F2** (b), **F3** (c), **F4** (d), **F5** (e), and **F6** (f) in EtOH.

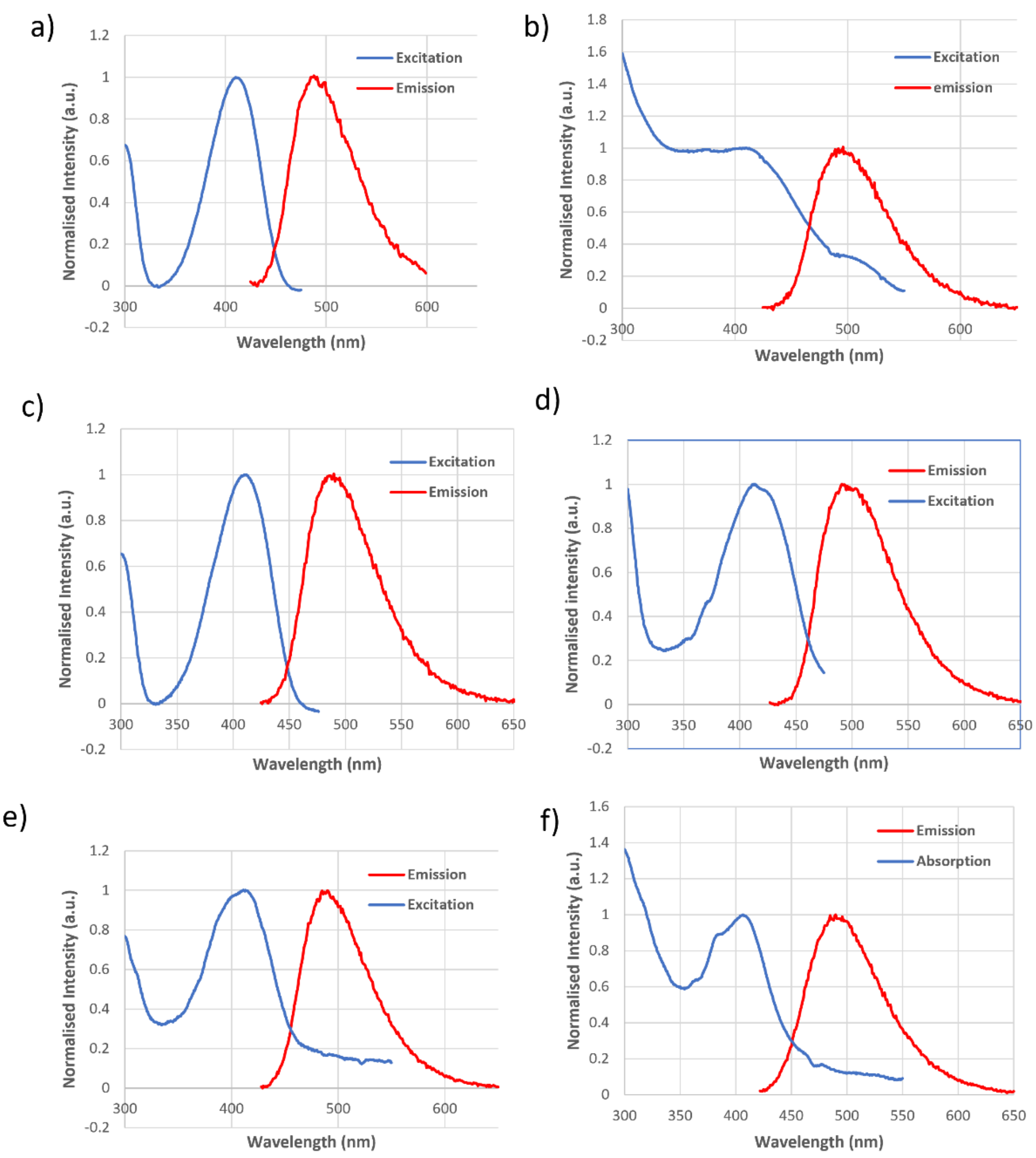

Figure S2. Excitation and emission spectra for compounds **F1** (a), **F2** (b), **F3** (c), **F4** (d), **F5** (e), and **F6** (f) in water.

Fluorescence quantum yield calculations

The fluorescence quantum yields for compounds **F1**–**F6** were measured by comparison with the standard fluorophore coumarin 6, which has a quantum yield of 0.78 in ethanol. Quantum yields were determined either in ethanol or water as the solvent. Experiments were performed in triplicate and the results are expressed as the mean.

The formula used to calculate the fluorescence quantum yield is:

$$\Phi_{\text{Sample}} = \Phi_{\text{Reference}} \times \frac{A_{\text{Sample}}}{A_{\text{Reference}}} \times \frac{Abs_{\text{Reference}}}{Abs_{\text{Sample}}} \times \frac{{n_{\text{Sample}}}^2}{{n_{\text{Reference}}}^2}$$

Φ = quantum yield, A = area under the fluorescence curve, Abs = absorbance, n = refraction index of the solvent.

4. Actin protein labelling

β-Actin protein was purified as described before.[29] The protein was desalted to remove DTT using desalting columns (Thermo Fisher Scientific, 89882). The column was pre-equilibrated and the sample was eluted with ice cold G buffer [5 mM HEPES pH 7.4, 0.2 mM $CaCl_2$, 0.01% (w/v) NaN_3, 0.2 mM ATP]. Then the monomeric actin was polymerised by addition of 10x MEK buffer [20 mM $MgCl_2$, 50 mM glycol-bis(2-aminoethylether)-*N*,*N*,*N′*,*N′*-tetraacetic (EGTA) and 1 M KCl] for 1 h at room temperature.

Compounds **F1**, **F2**, **F3** and **F4** were dissolved in DMSO and left at room temperature for 3 h. Then each sample was incubated with polymerised actin using a molar ratio 1:10 actin to fluorophore for 1 h at room temperature. The reaction was quenched by addition of 10 mM of dithiothreitol (DTT). The protein was pelleted at 100000 g at 4 °C for 1 h. Filamentous actin was depolymerised by dialysing the actin in G buffer with DTT for 2 days.

Imaging actin protein filaments

The actin proteins labelled with fluorophores were visualised in chambers made of a microscope slide and a coverslip. A coverslip was coated with 1% nitrocellulose. Then three strips of double-sided tape were attached on a slide with 2–3 mm gaps between the tape strips. Finally, a coverslip was attached on top of the tape forming two chambers. Then 1 mg/ml of myosin (Hypermol, 8306-01) was loaded into a chamber and incubated for 10 min. It was washed with 20 µL of running buffer twice. Labelled actin filaments were diluted with running buffer to 100 nM. 20 µL of actin filaments was loaded into chamber followed by sealing with nail polish. The sample was imaged with a confocal fluorescence microscope straight away. Actin polymers were visualised using 405 nm excitation wavelength and GFP filter. The fluorescence intensity of actin filaments was quantified using Fiji image processing package using single particle analysis function. Two images of each compound were analysed.

SUMO-NLS construct generation and protein purification

Primers containing the NLS and 3x cysteines (F: CTGCACCAAACTGGAGGCCAGCCGAAGAAGAAACGCAAAGTGGGCAGCCCGAAGAAGAAACGCAAAGTGGGCAGCTGCGGCAGCTGCGGCAGCTGCTAATGCAAGCTTGCGGCCGCACT; R: AGTGCGGCCGCAAGCTTGCATTAGCAGCTGCCGCAGCTGCCGCAGCTGCCCACTTTGCGTTTCTTCTTCGGGCTGCCCACTTTGCGTTTCTTCTTCGGCTGGCCTCCAGTTGGTGCAG) were annealed and Gibson cloned into an NdeI restricted and linearised pET-SUMO vector containing a 6x His tag. The cloned construct was transformed into BL21(DE3) *E.coli* cells. The cells were grown to 0.6 OD and were induced with 0.5 mM IPTG at 30 °C for 4 hours. The cells were lysed by lysozyme treatment followed by sonication in buffer containing 50 mM sodium phosphate buffer pH 7.5, 150 mM NaCl and 10 mM imidazole and the protein pulled down using Ni-NTA beads. The beads were washed with buffer consisting of 50 mM sodium phosphate buffer pH 7.5, 500 mM NaCl and 30 mM imidazole. The protein was eluted using the same buffer containing 500 mM imidazole. DTT to a concentration of 5 mM was added to it and the buffer was exchanged into 1x phosphate buffered saline (PBS) using a PD10 column. For dye labelling, a 3.5 molar excess of the malemide dye was added to the purified protein, which was incubated at room temperature for 1 hour.[30] The excess dye was then washed out using a 3 kD cutoff filter. The protein was then aliquoted and stored frozen at –80 °C. The labelling efficiency for **F2** with SUMO-NLS protein was determined to be ca. 2.88.

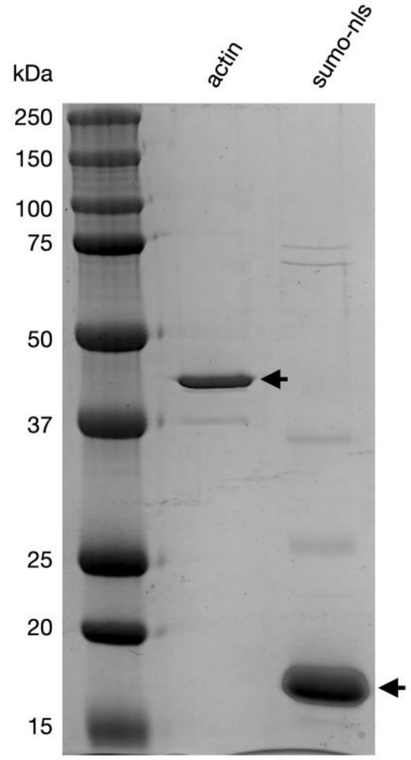

Figure S3. Gel for purified and labelled actin and SUMO-NLS protein next to protein ladder.

Microinjection, mounting and imaging of zebrafish

Experiments involving zebrafish embryos were authorised by the *Animal Welfare & Ethical Review Body* (AWERB) at the University of Warwick, which ensured that UK Home Office guidelines were fully followed. Wild-type fish of the Tübingen or Ab backgrounds were used. Adult fish were kept at the University of Warwick animal facility at their ambient temperature. Only wild-type embryos less than a day of fertilisation were used. Embryos from pooled matings were collected in 0.3X Danieau's buffer with methylene blue (17 mM NaCl, 2 mM KCl, 0.12 mM $MgSO_4$, 1.8 mM, $Ca(NO_3)_2$, 1.5 mM HEPES, pH 7.6). Embryos at the 1-4 cell stage were injected with ~1–2 ng of labelled actin protein or ~4 ng of labelled or unlabelled SUMO-NLS protein. The embryos were grown for 3 hours at 28.5 °C. The embryos were then manually dechorionated with forceps, mounted in agarose moulds[36] and imaged on an Andor SD spinning disk microscope, which consisted of a Nikon Eclipse Ti inverted microscope, a Nikon Plan Apo λ 20x/0.75 dry objective or a Nikon Plan Fluor 40x/1.30 oil immersion objective. Images were collected using an Andor iXon Ultra EMCCD camera. All imaging was performed using live embryos at 28.5 °C. For imaging actin, a total of 6 embryos from two separate experiments were used. For the SUMO-NLS protein, a total of 8 embryos from three different experiments were examined.

5. Depiction of single crystal X-ray structures

Single Crystal X-ray Structure of 6 (CCDC 2295310). Single crystals of $C_{13}H_9F_2N_7OS$ were grown from 8:2 mixture of hexane and ethyl acetate. A suitable crystal was selected and mounted on a XtaLAB Synergy, Dualflex, HyPix diffractometer. The crystal was kept at 100(2) K during data collection. Using Olex2,[37] the structure was solved with the SHELXT[38] structure solution program using Intrinsic Phasing and refined with the SHELXL[38] refinement package using Least Squares minimisation.

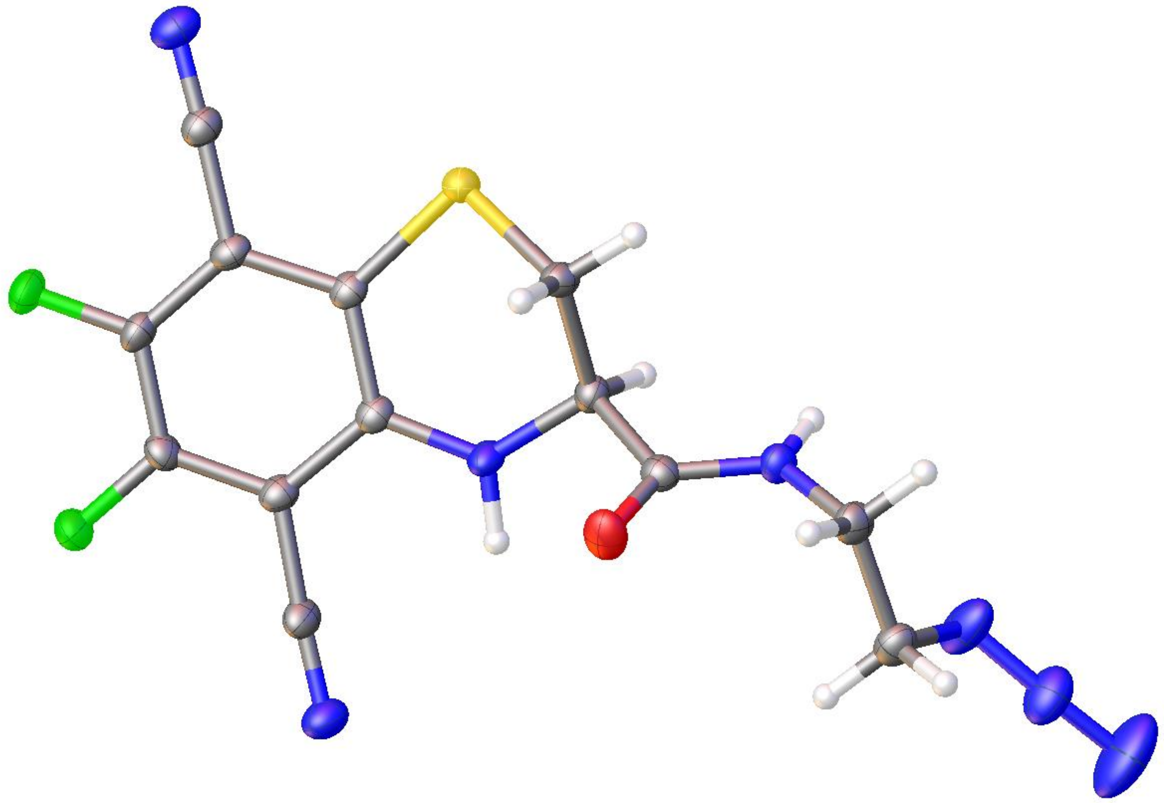

Crystal Data for $C_{13}H_9F_2N_7OS$ (M =349.33 g/mol): orthorhombic, space group $P2_12_12_1$ (no. 19), a = 4.71070(10) Å, b = 14.1474(2) Å, c = 22.7544(3) Å, V = 1516.45(4) Å^3, Z = 4, T = 100(2) K, μ(Cu Kα) = 2.283 mm^{-1}, $Dcalc$ = 1.530 g/cm^3, 48172 reflections measured (7.358° ≤ 2Θ ≤ 160.618°), 3255 unique (R_{int} = 0.0951, R_{sigma} = 0.0270) which were used in all calculations. The final R_1 was 0.0365 (I > 2σ(I)) and wR_2 was 0.0982 (all data). The Flack x and Hooft y parameters were respectively 0.002(8) (Shelx2018) and -0.014(6) (Olex2). Additionally, the synthesis was conducted from a starting material of known handedness.

Single Crystal X-ray Structure of 9 (CCDC 2295311). Single crystals of $C_{12}H_8F_2N_4O_2$ were grown from 8:2 mixture of hexane and dichloromethane. A suitable crystal was selected and mounted on a glass fibre with Fomblin oil and placed on a Rigaku Oxford Diffraction Super Nova Diffractometer with a duel source (Cu at zero) equipped with an Atlas S2 CCD area detector. The crystal was kept at 150(2) K during data collection.

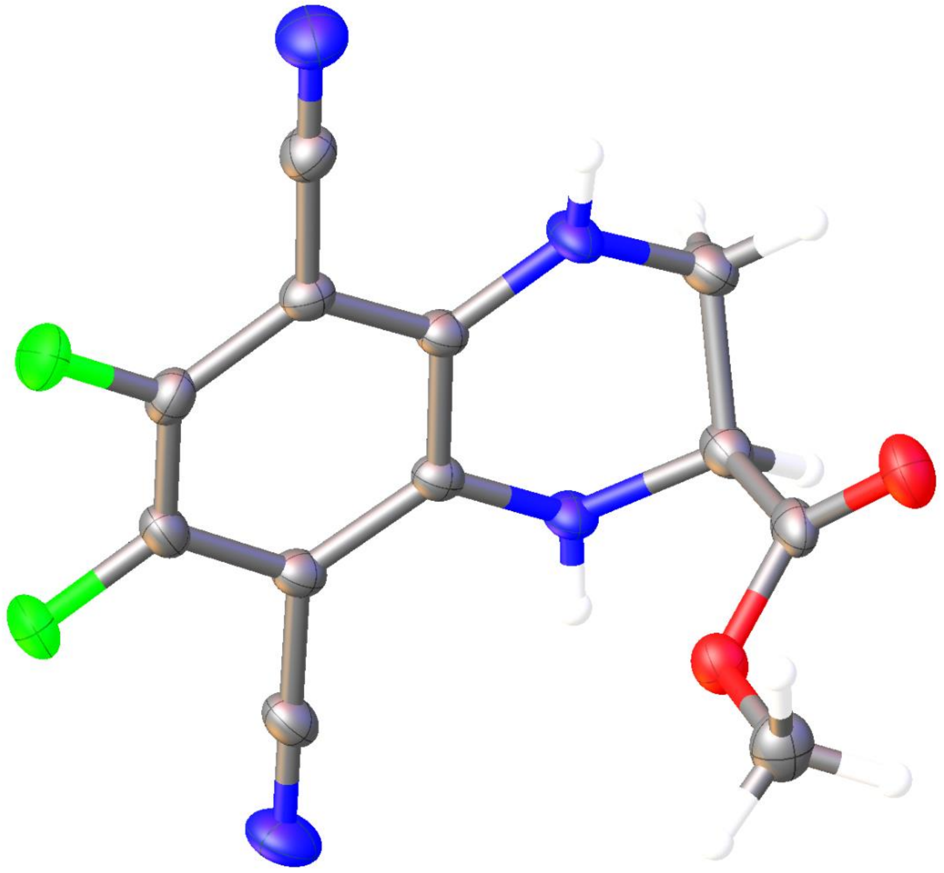

Crystal Data for $C_{12}H_8F_2N_4O_2$ (M =278.22 g/mol): orthorhombic, space group Pbca (no. 61), a = 12.96980(10) Å, b = 8.91900(10) Å, c = 19.92040(10) Å, V = 2304.34(3) Å^3, Z = 8, T = 150(2) K, μ(Cu Kα) = 1.173 mm^{-1}, $Dcalc$ = 1.604 g/cm^3, 33370 reflections measured (8.878° ≤ 2Θ ≤ 147.132°), 2323 unique (R_{int} = 0.0282, R_{sigma} = 0.0133) which were used in all calculations. The final R_1 was 0.0349 (I > 2σ(I)) and wR_2 was 0.1014 (all data).

Single Crystal X-ray Structure of 12 (CCDC 2295312). Single crystals of $C_{26}H_{24}Cl_2F_4N_{10}O_{4.1}S_2$ were grown by vapour diffusion of ethyl acetate into a methanol solution of the compound. A suitable crystal was selected and mounted on a Mitegen head with Fomblin oil and placed on a Rigaku Oxford Diffraction Synergy-S diffractometer with a dual source equipped with a Hybrid pixel array detector. The crystal was kept at 100(2) K during data collection.

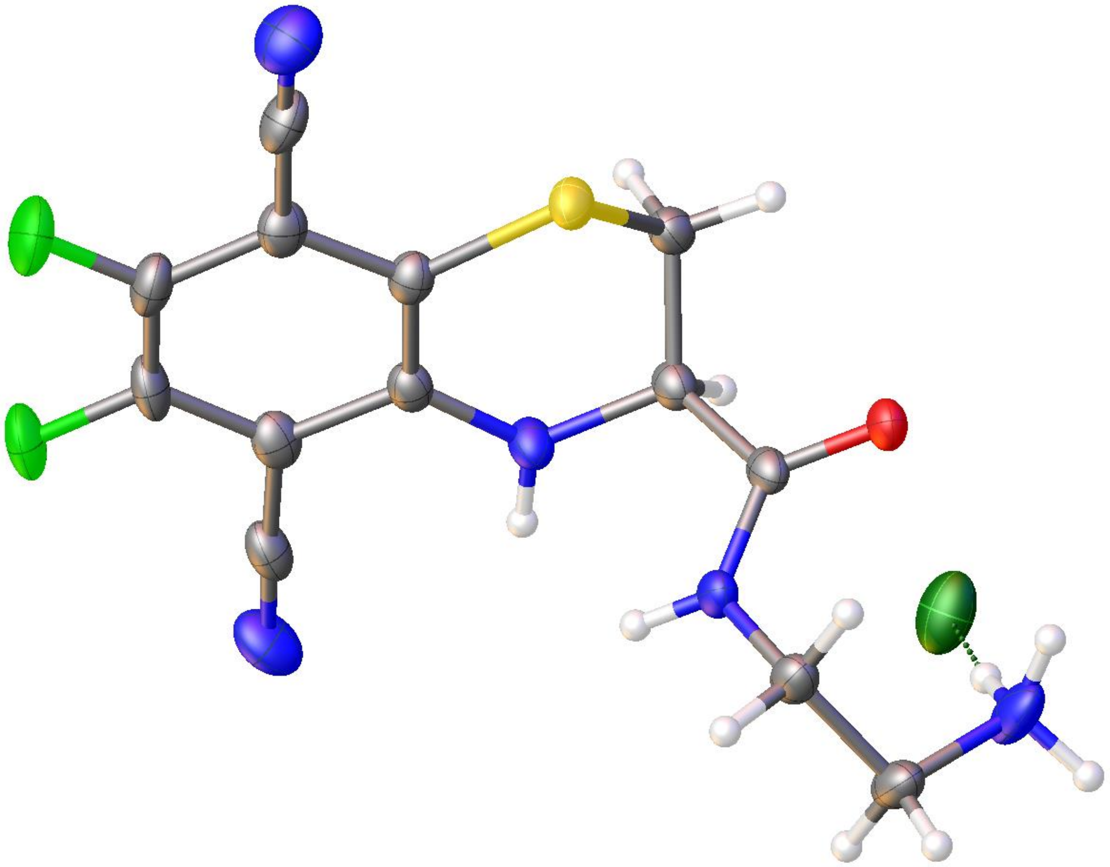

Crystal Data for $C_{26}H_{24}Cl_2F_4N_{10}O_4S_2$ (M =751.57 g/mol): monoclinic, space group C2 (no. 5), a = 16.1668(2) Å, b = 8.11270(10) Å, c = 25.9487(3) Å, β = 99.4980(10)°, V = 3356.68(7) Å^3, Z = 4, T = 100(2) K, μ(CuKα) = 3.541 mm^{-1}, $Dcalc$ = 1.487 g/cm^3, 44181 reflections measured (6.908° ≤ 2Θ ≤ 160.03°), 7137 unique (R_{int} = 0.0690, R_{sigma} = 0.0409) which were used in all calculations. The final R_1 was 0.0523 (I > 2σ(I)) and wR_2 was 0.1445 (all data). The Flack x parameter was 0.01(2) by classical fit to all intensities and 0.006(10) from 2906 selected quotients (Parsons' method) (Shelx2019) and the Hooft y parameter was 0.009(6) (Olex2). Additionally, the synthesis was conducted from a starting material of known handedness.

Single Crystal X-ray Structure of F1 (CCDC 2295313). Single crystals of $C_{17}H_{11}F_2N_5O_3S$ were grown by vapour diffusion of acetone/ethanol. A suitable crystal was selected and mounted on a Mitegen head with Fomblin oil and placed on a Rigaku Oxford Diffraction Synergy-S diffractometer with a dual source equipped with a Hybrid pixel array detector. The crystal was kept at 150(2) K during data collection. Using Olex2,[37] the structure was solved with the SHELXT[38] structure solution program using Intrinsic Phasing and refined with the SHELXL[38] refinement package using Least Squares minimisation.

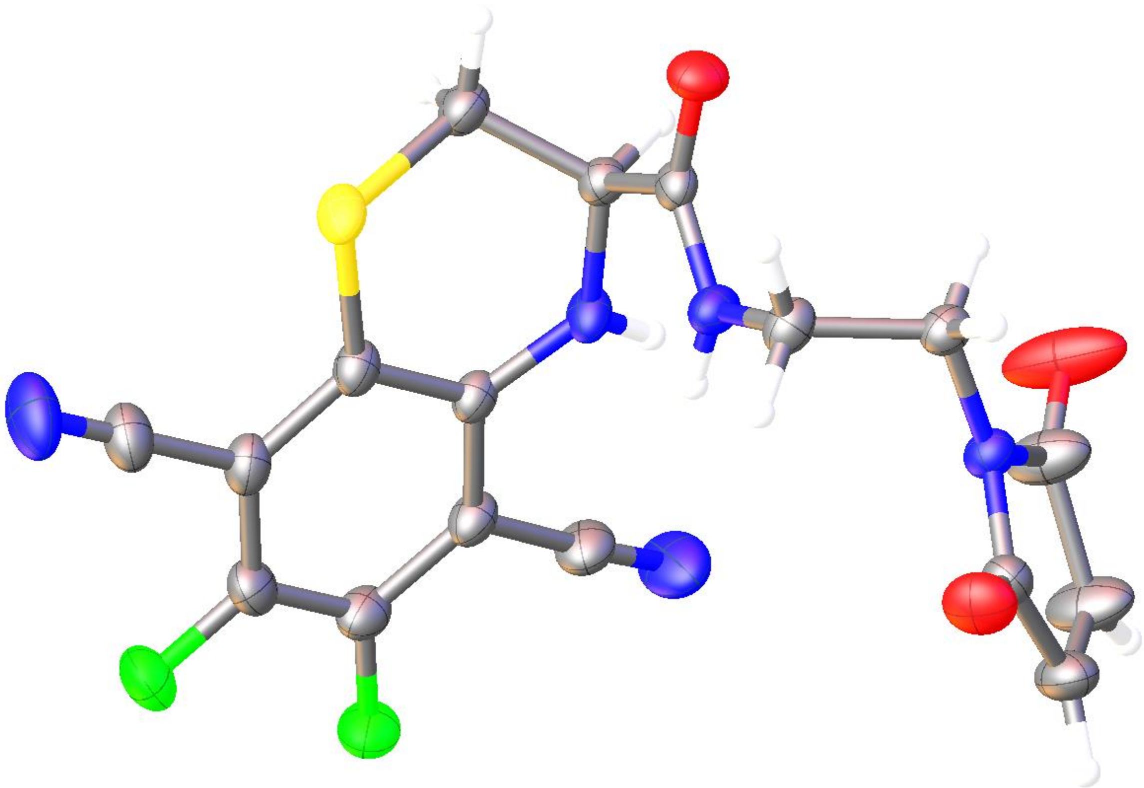

Crystal Data for $C_{17}H_{11}F_2N_5O_3S$ (M =403.37 g/mol): orthorhombic, space group $P2_12_12_1$ (no. 19), a = 7.55286(4) Å, b = 8.90166(5) Å, c = 26.43926(12) Å, V = 1777.592(16) Å^3, Z = 4, T = 150(2) K, μ(Cu Kα) = 2.088 mm^{-1}, $Dcalc$ = 1.507 g/cm^3, 58260 reflections measured (6.686° ≤ 2Θ ≤ 147.252°), 3591 unique (R_{int} = 0.0627, R_{sigma} = 0.0180) which were used in all calculations. The final R_1 was 0.0311 (I > 2σ(I)) and wR_2 was 0.0825 (all data). The Flack x parameter was 0.006(23) by classical fit to all intensities and 0.000(12) from 1472 selected quotients (Parsons' method) (Shelx2018) and the Hooft y parameter was -0.005(5) (Olex2). Additionally, the synthesis was conducted from a starting material of known handedness.

6. Copies of ^{1}H, ^{13}C and ^{19}F NMR spectra

^{1}H NMR (400 MHz, DMSO-d_6)

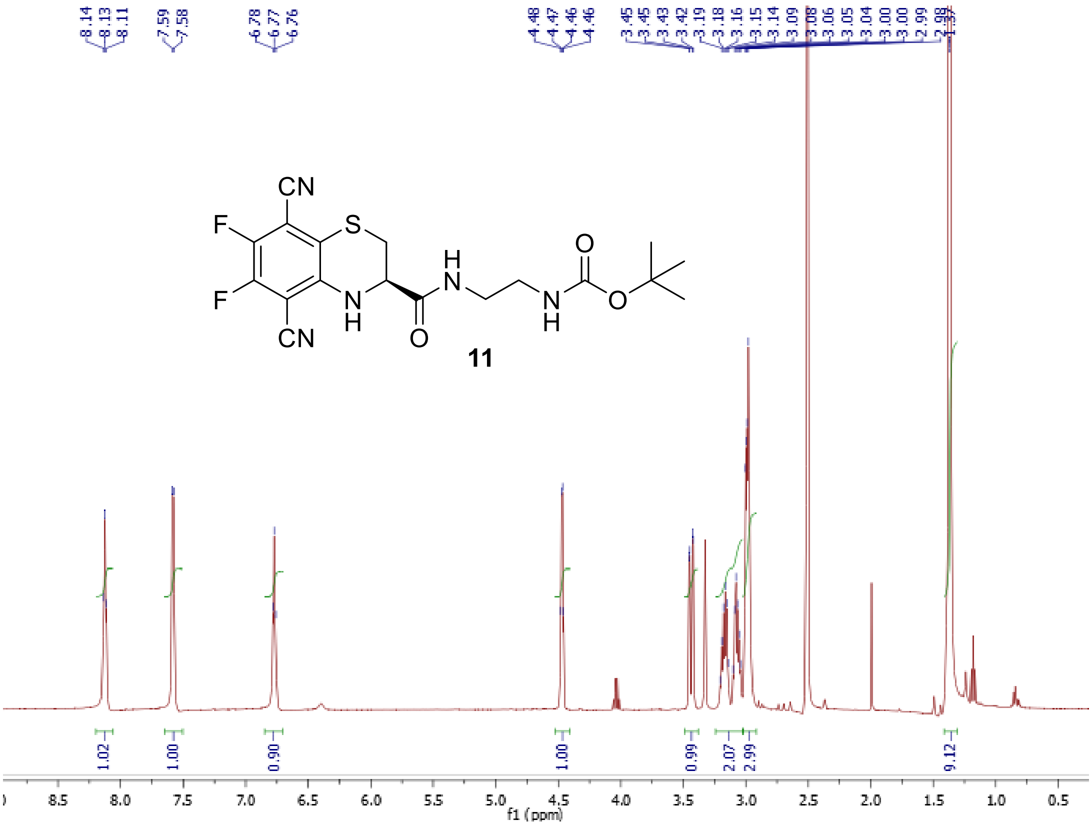

^{13}C NMR (126 MHz, DMSO-d_6)

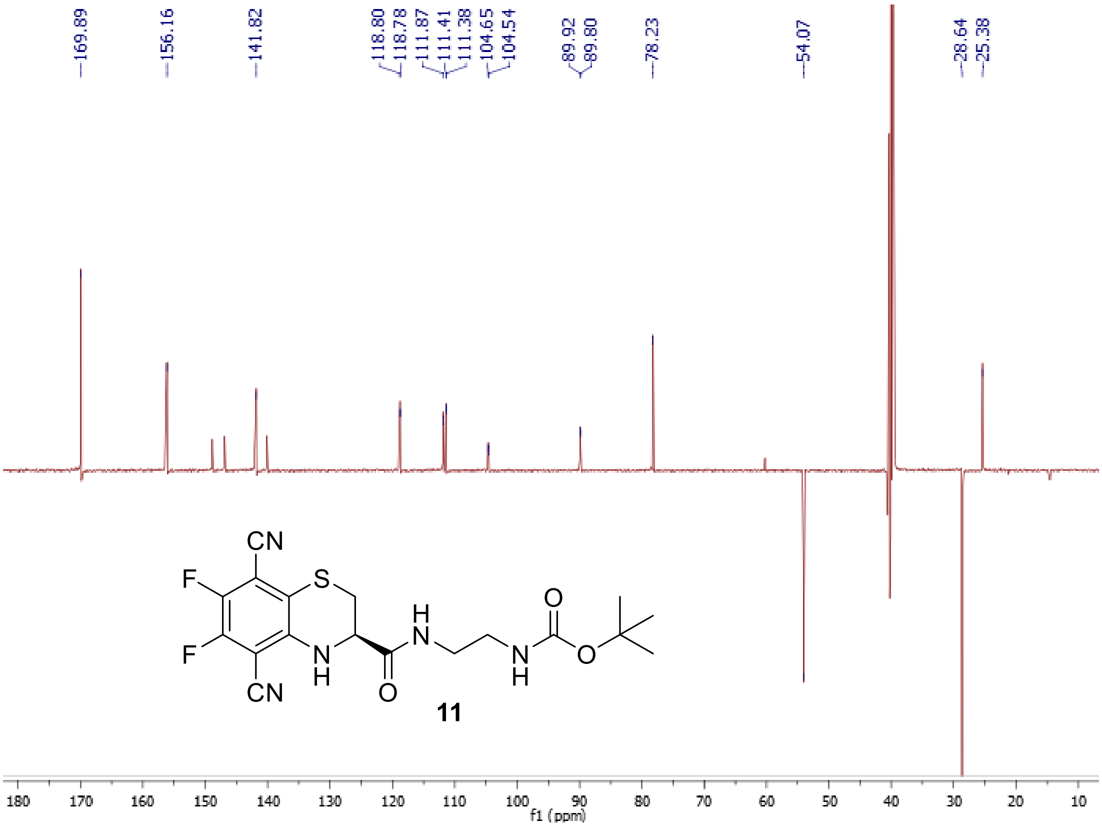

^{19}F NMR (376 MHz, DMSO-d_6)

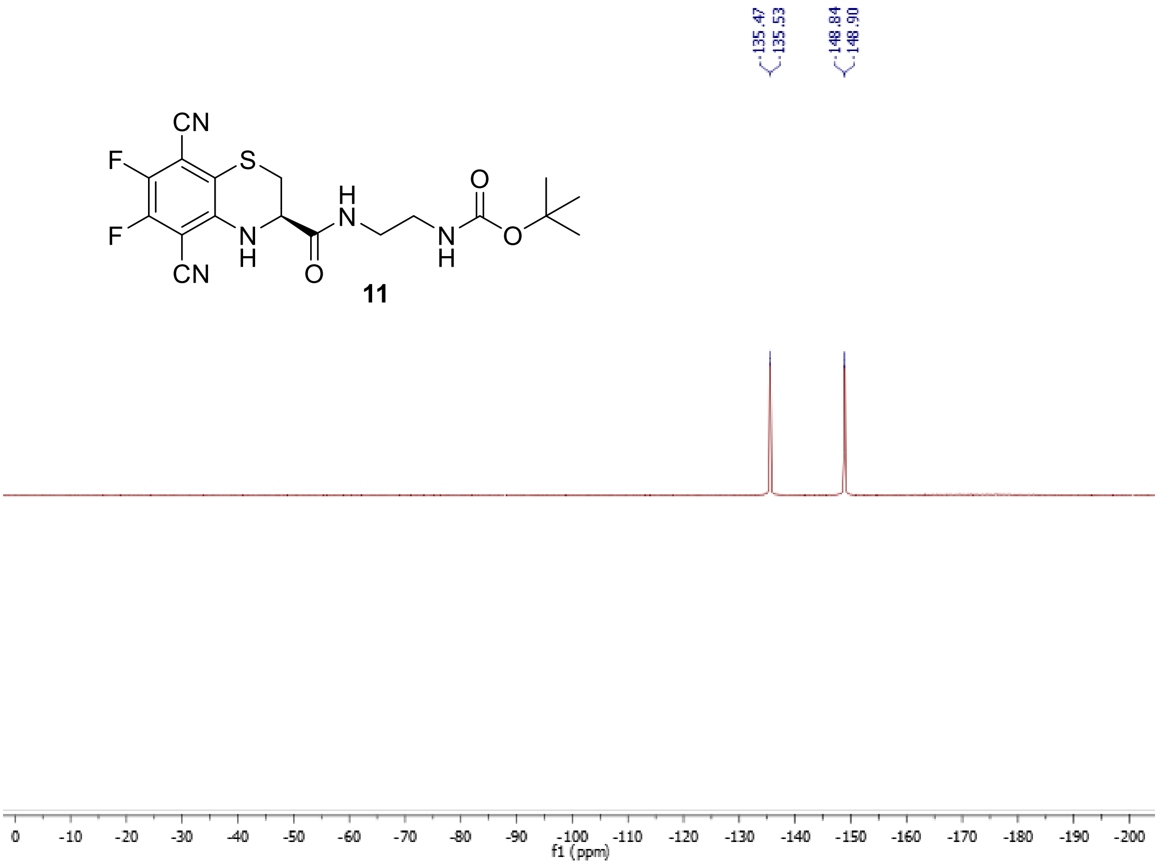

^{1}H NMR (400 MHz, DMSO-d_6)

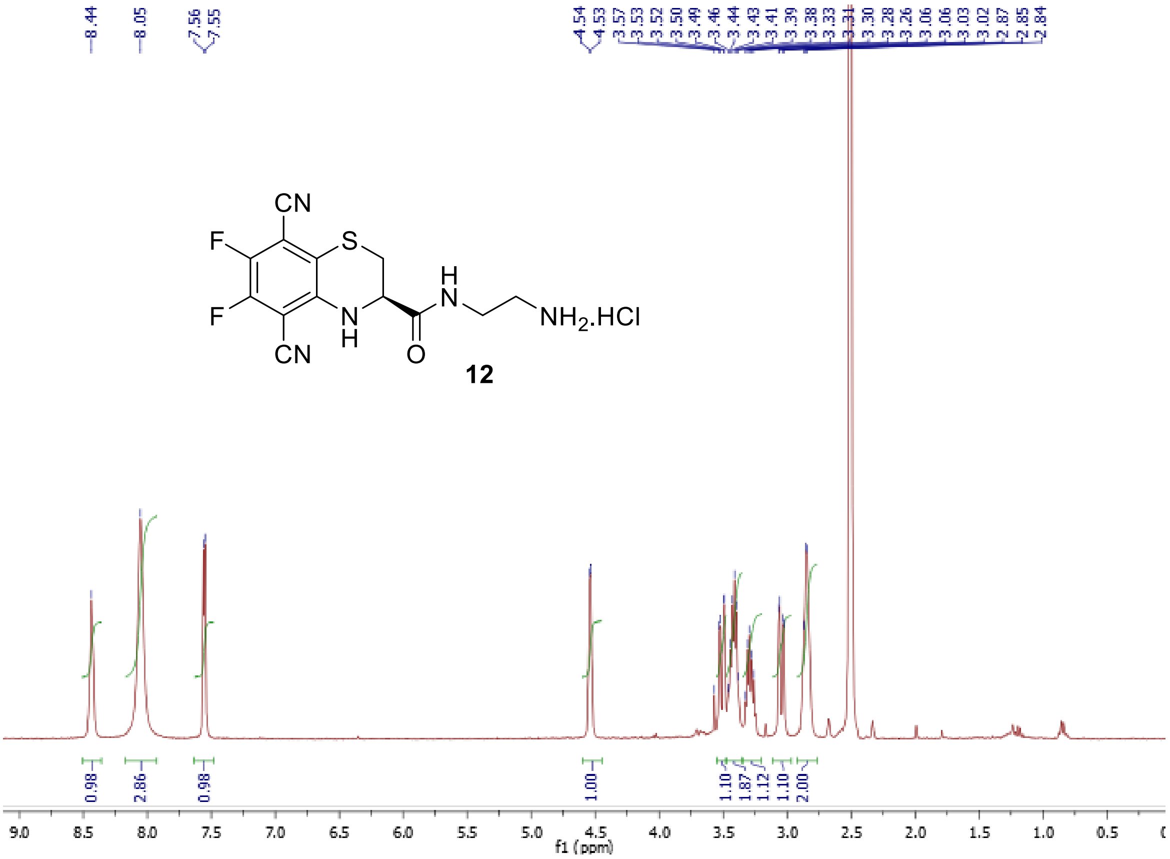

^{13}C NMR (126 MHz, DMSO-d_6)

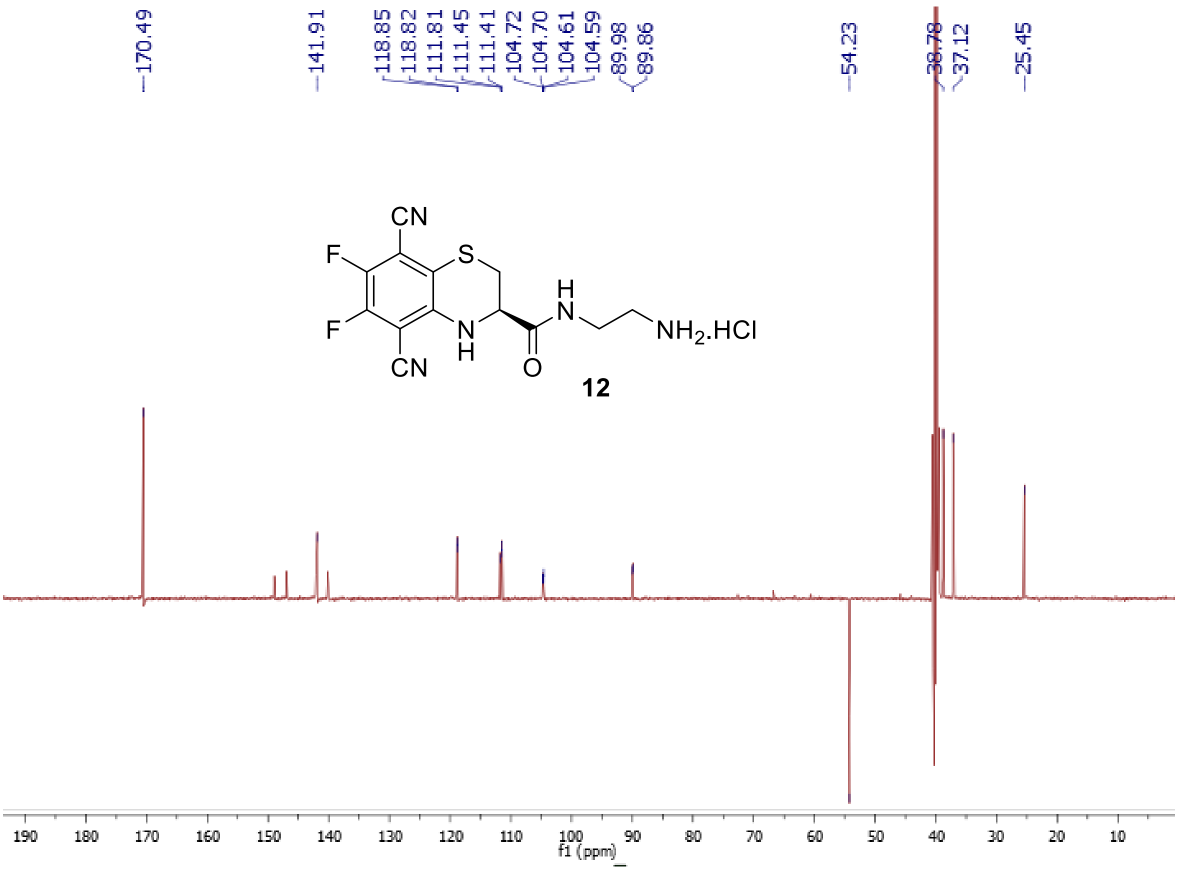

^{19}F NMR (376 MHz, DMSO-d_6)

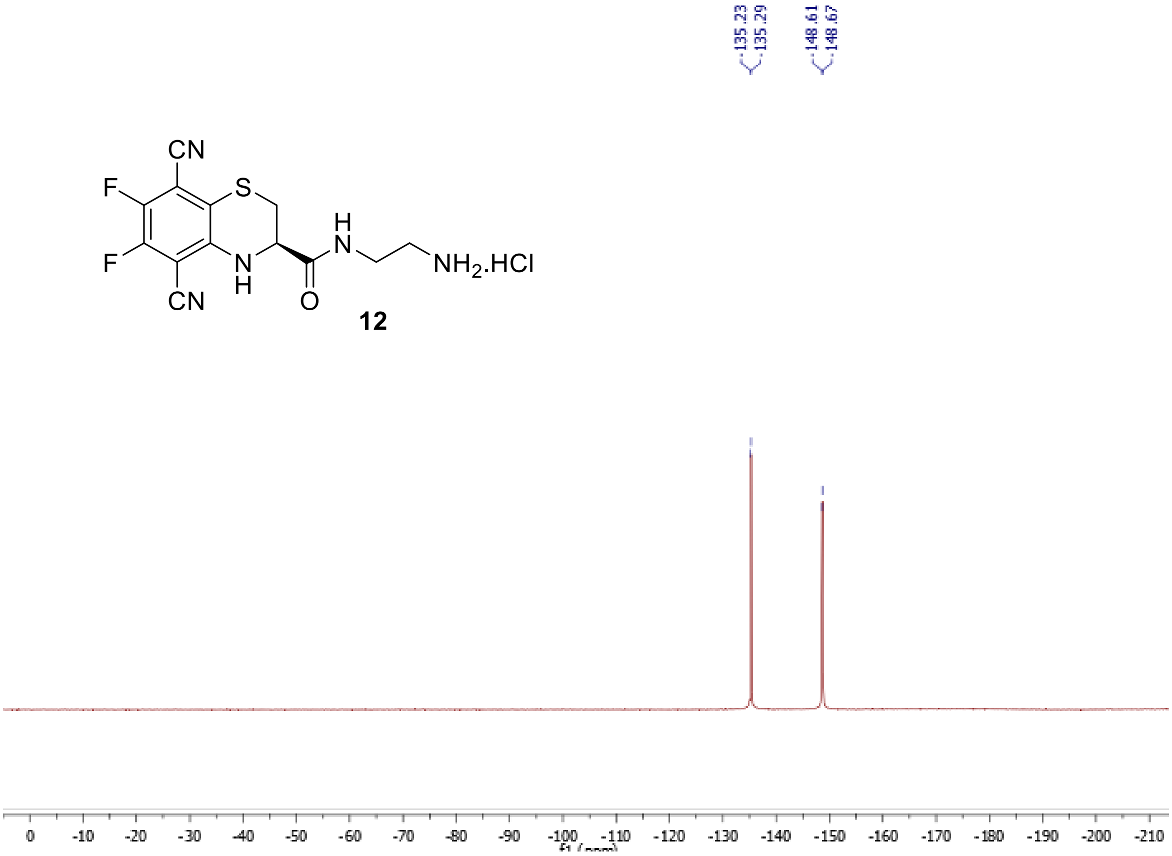

^{1}H NMR (400 MHz, DMSO-d_6)

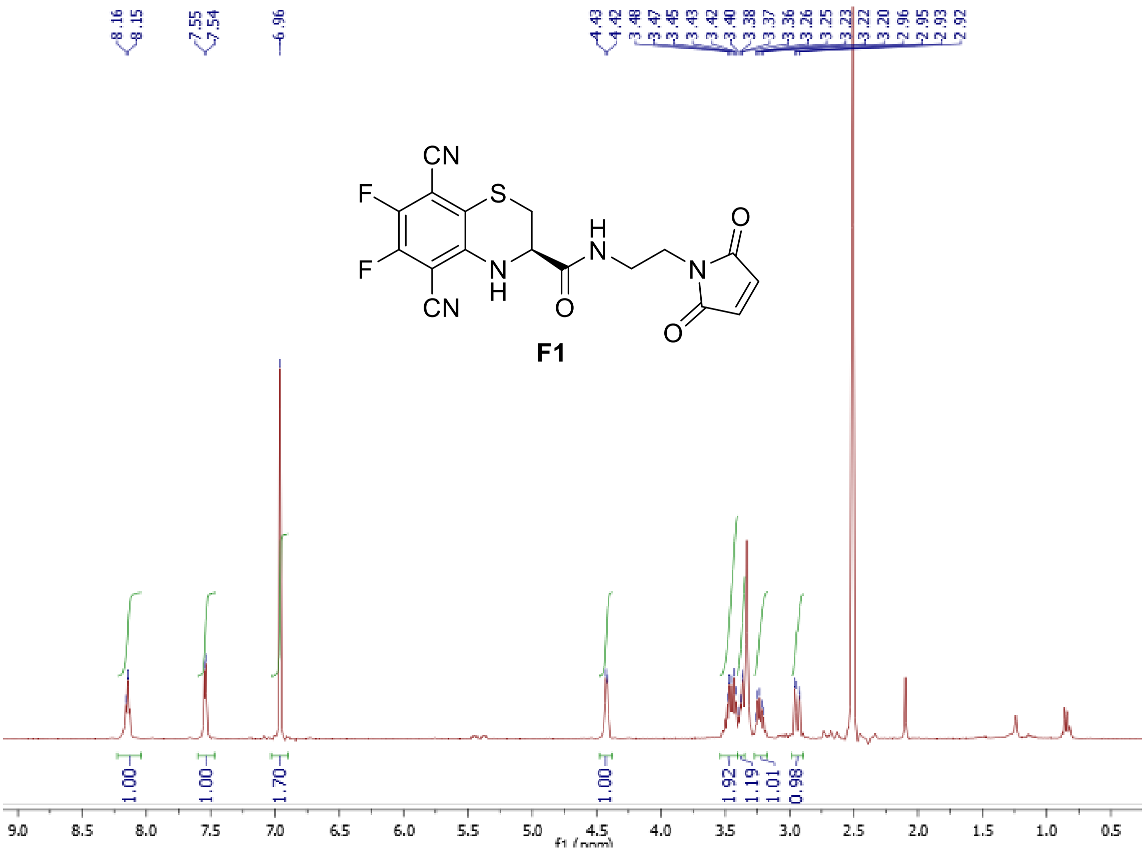

^{13}C NMR (126 MHz, DMSO-d_6)

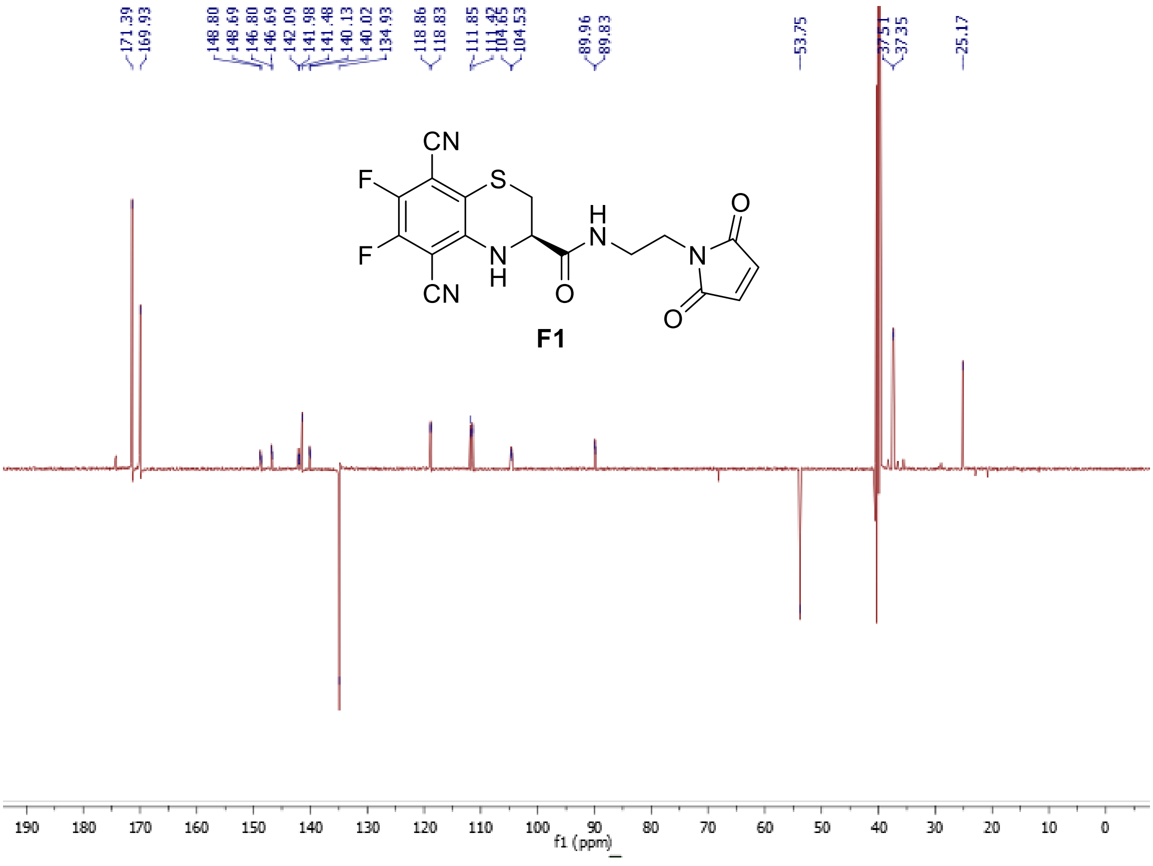

^{19}F NMR (376 MHz, DMSO-d_6)

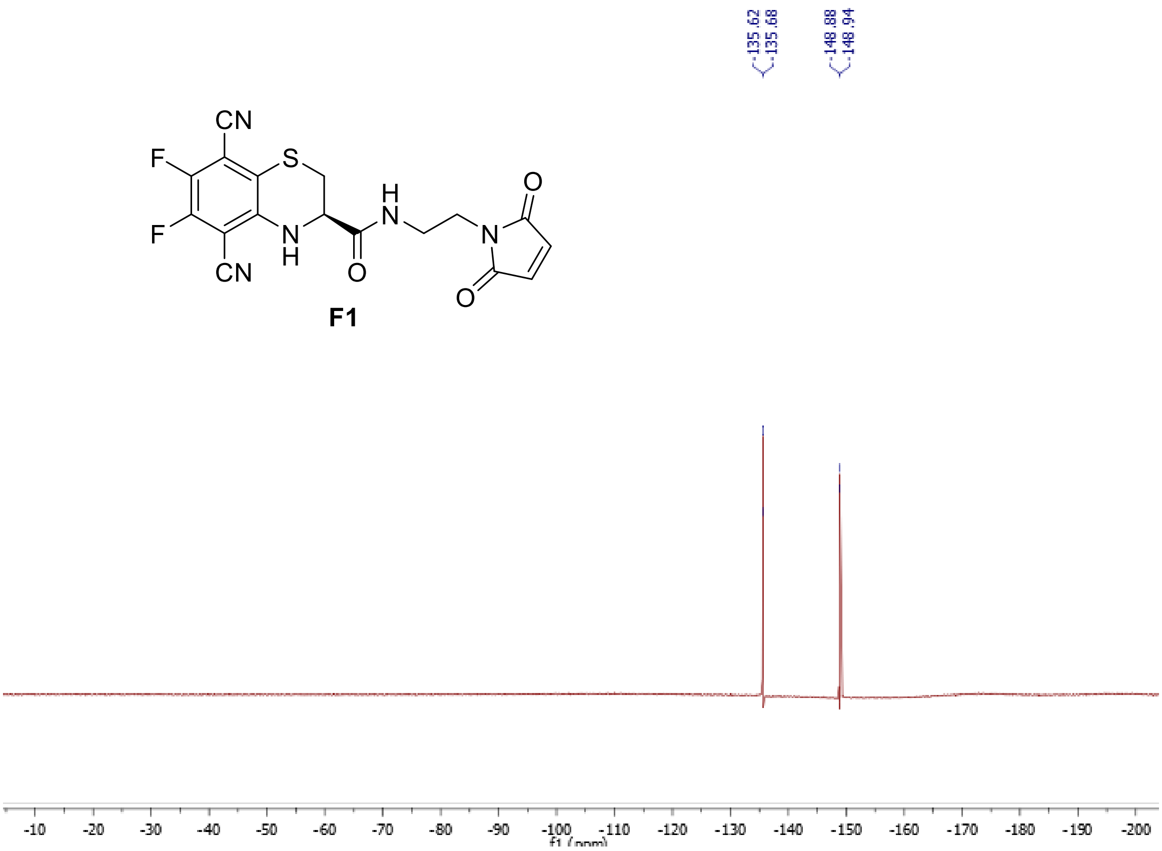

^{1}H NMR (400 MHz, DMSO-d_6)

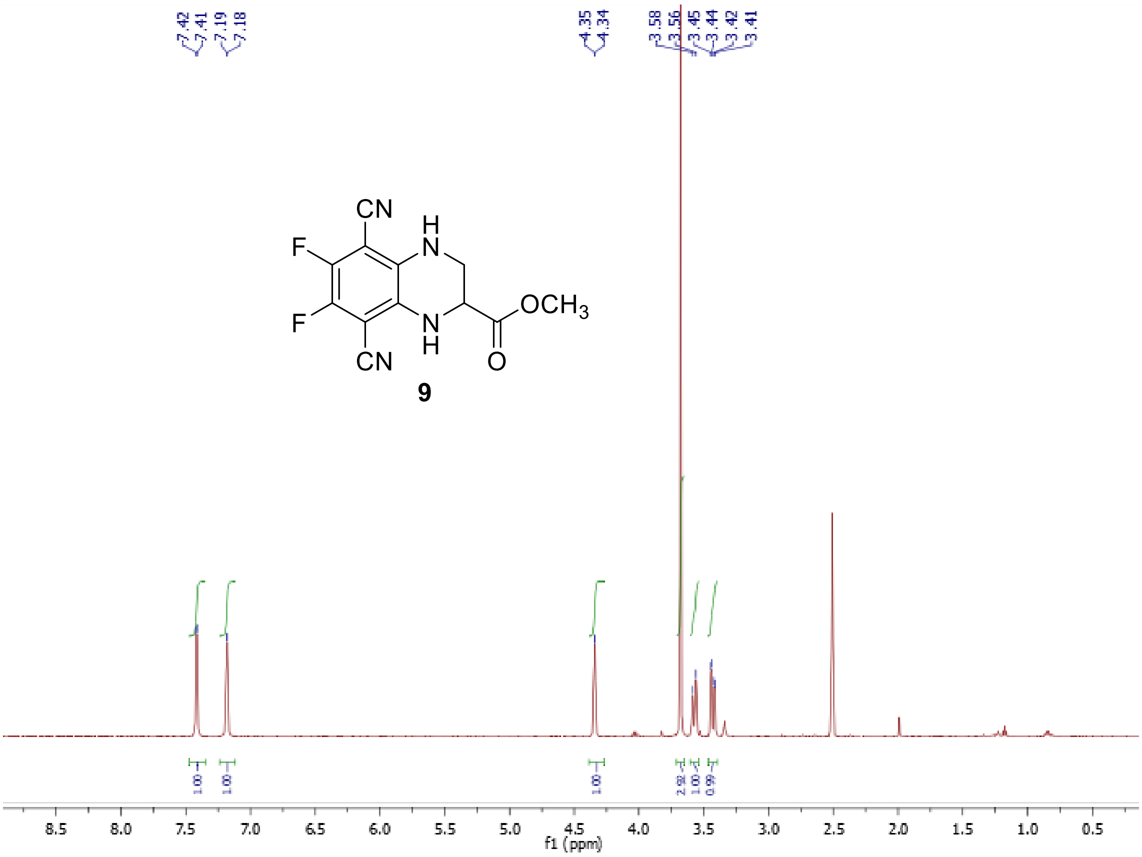

^{13}C NMR (126 MHz, DMSO-d_6)

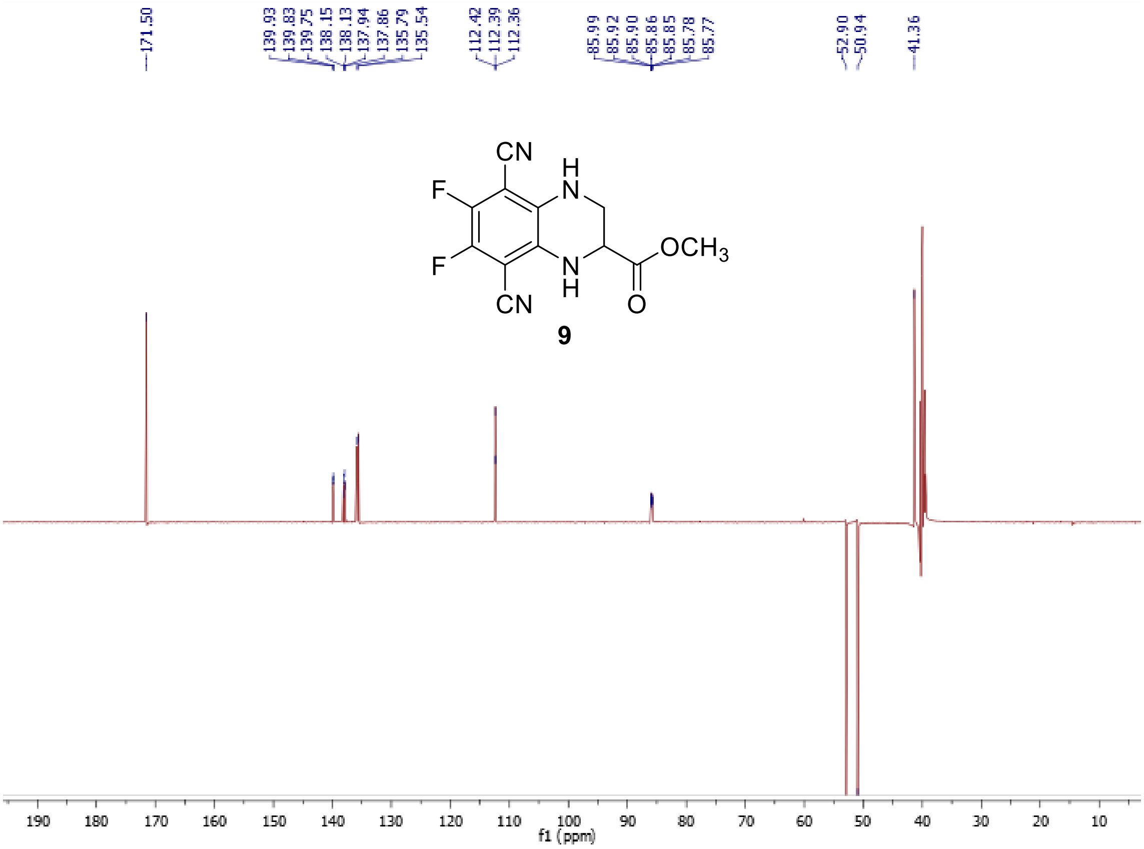

^{19}F NMR (376 MHz, DMSO-d_6)

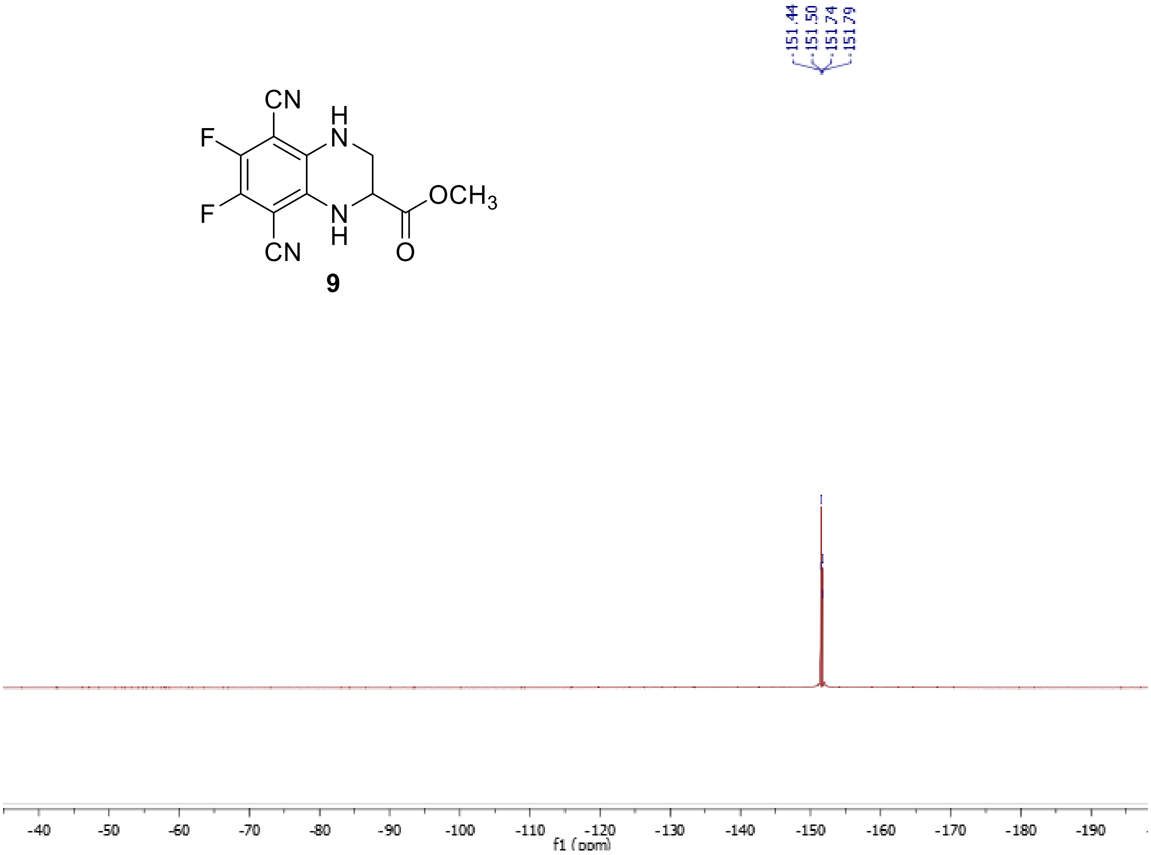

^{1}H NMR (400 MHz, DMSO-d_6)

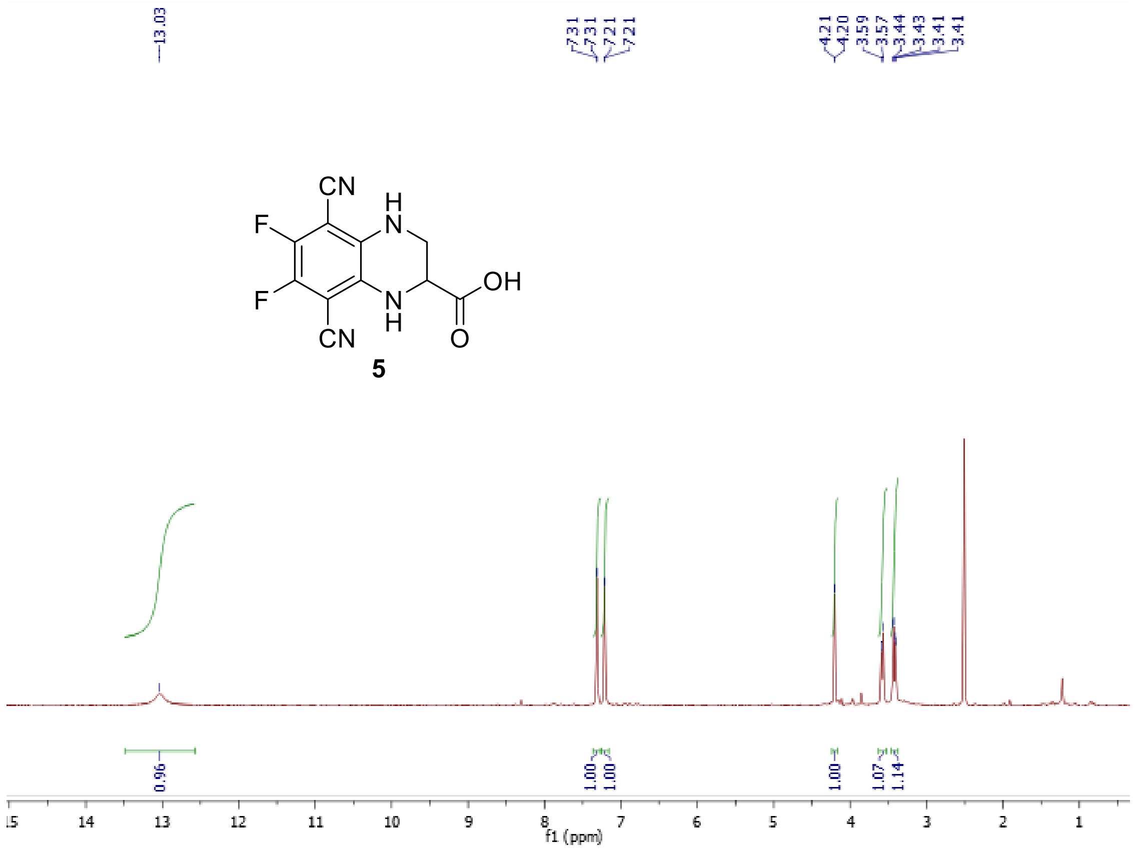

^{13}C NMR (126 MHz, DMSO-d_6)

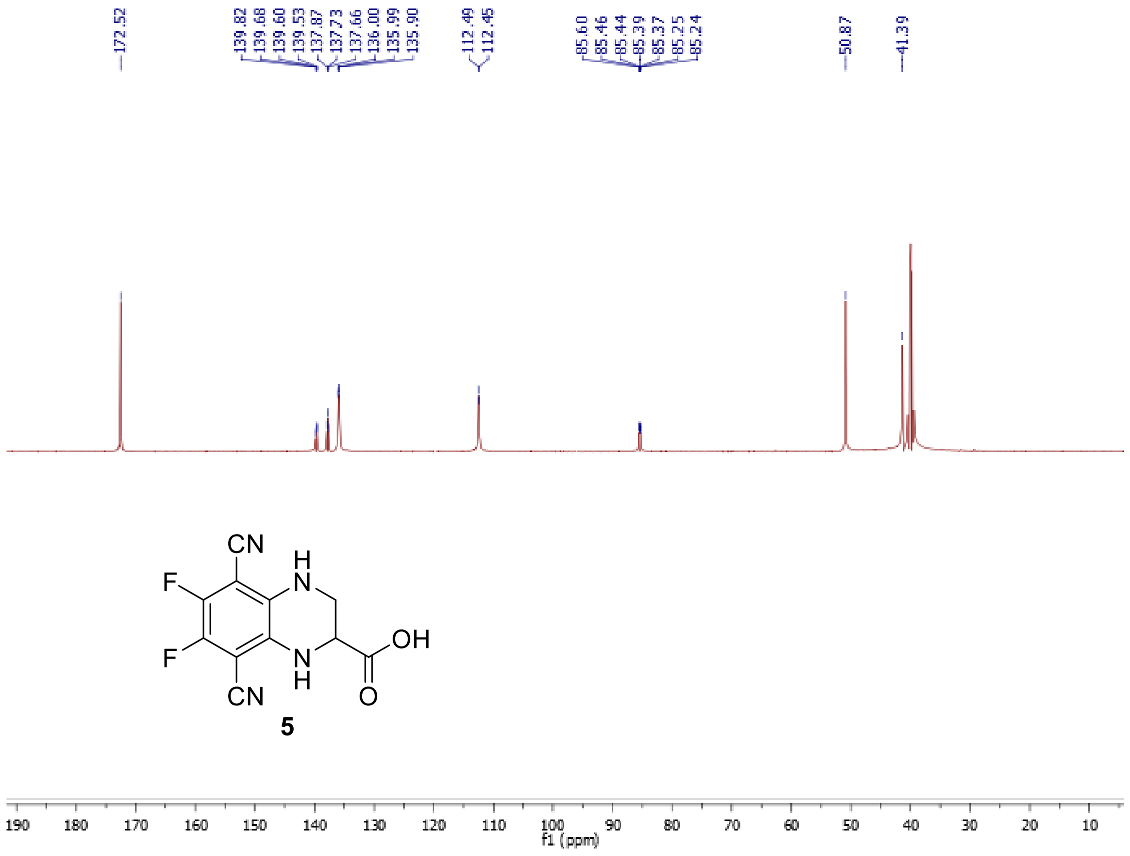

^{19}F NMR (376 MHz, DMSO-d_6)

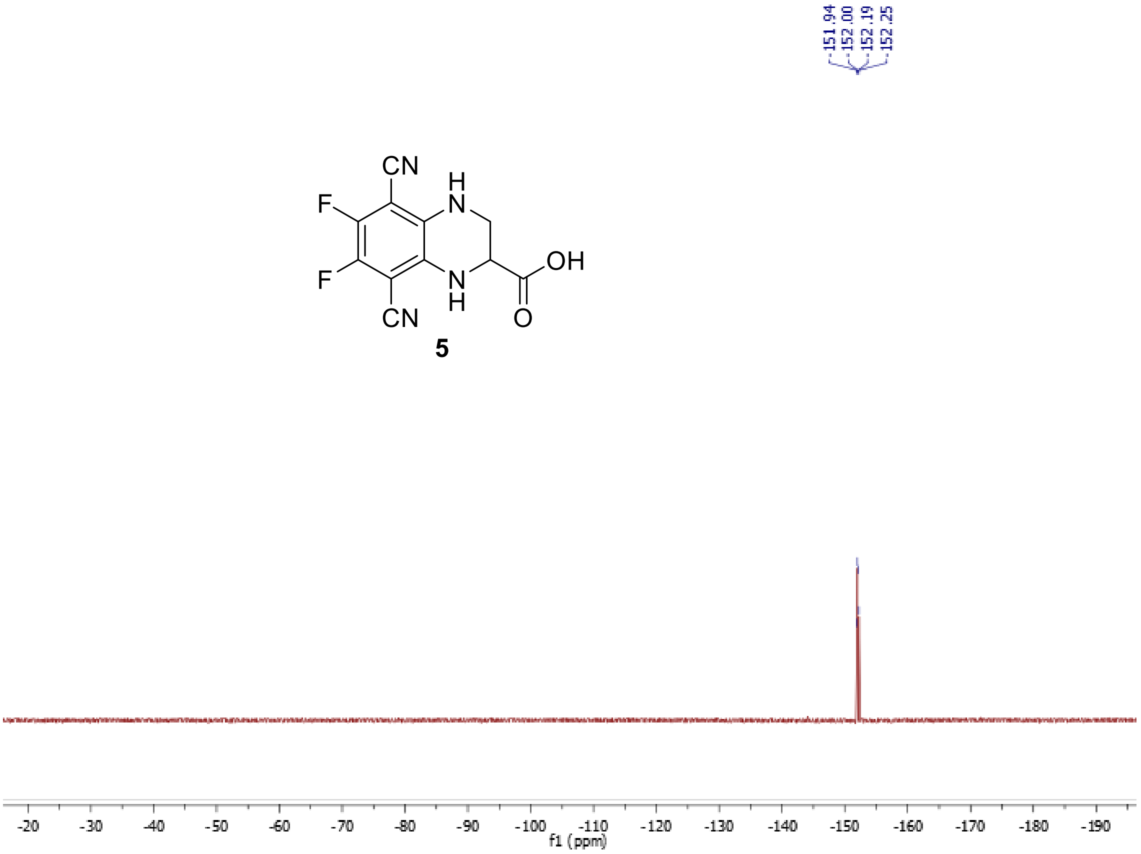

^{1}H NMR (400 MHz, DMSO-d_6)

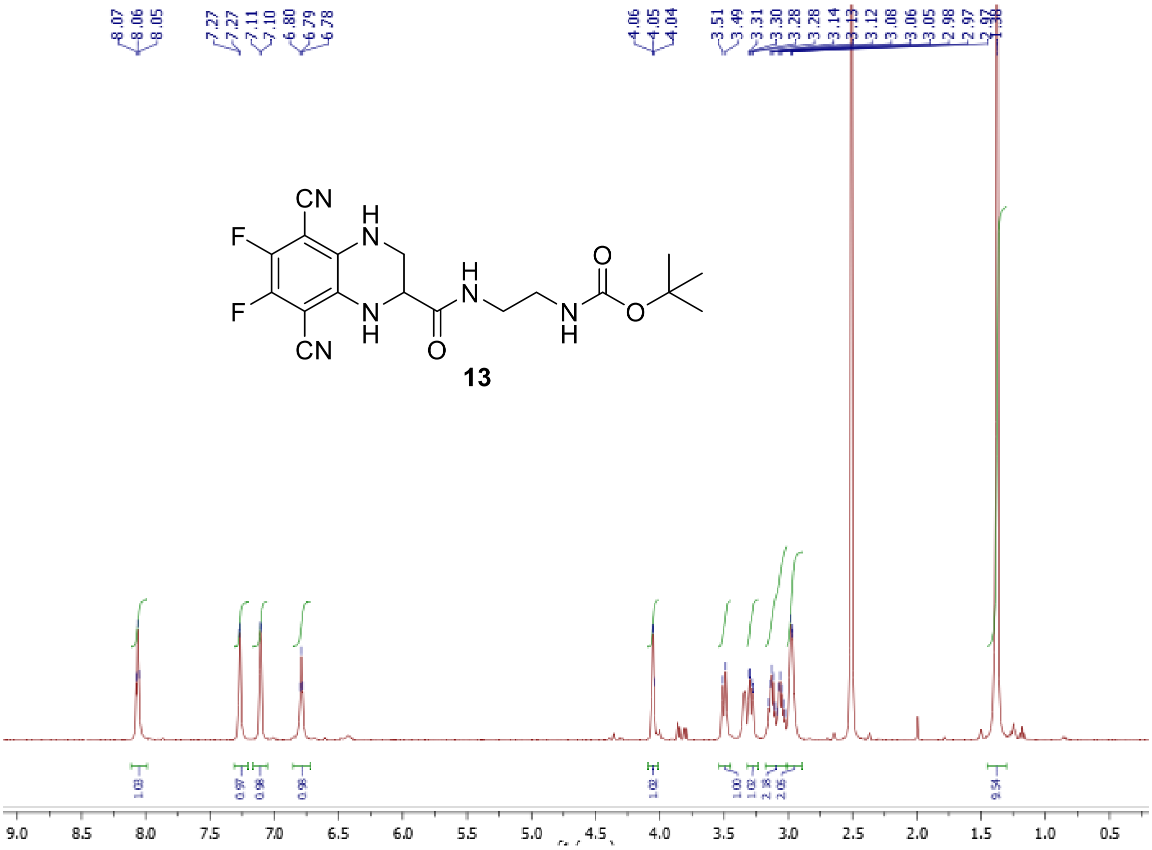

^{13}C NMR (126 MHz, DMSO-d_6)

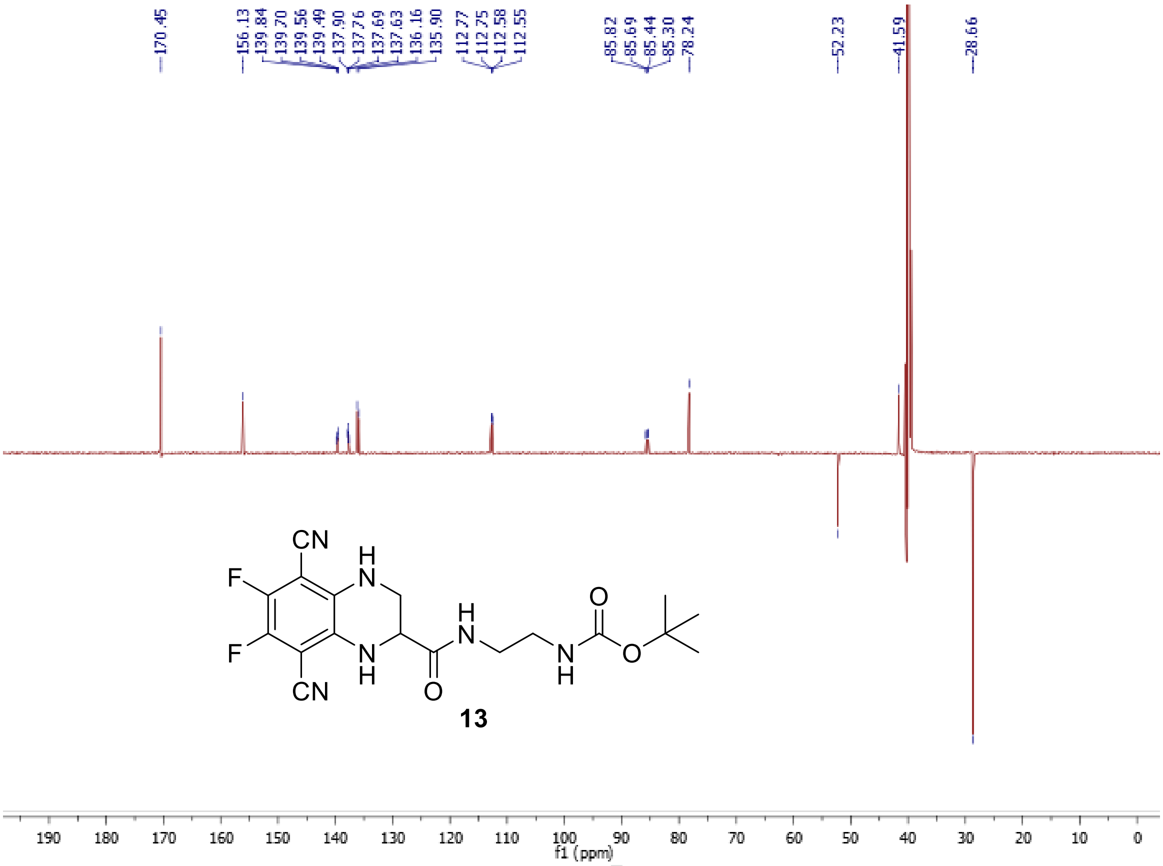

^{19}F NMR (376 MHz, DMSO-d_6)

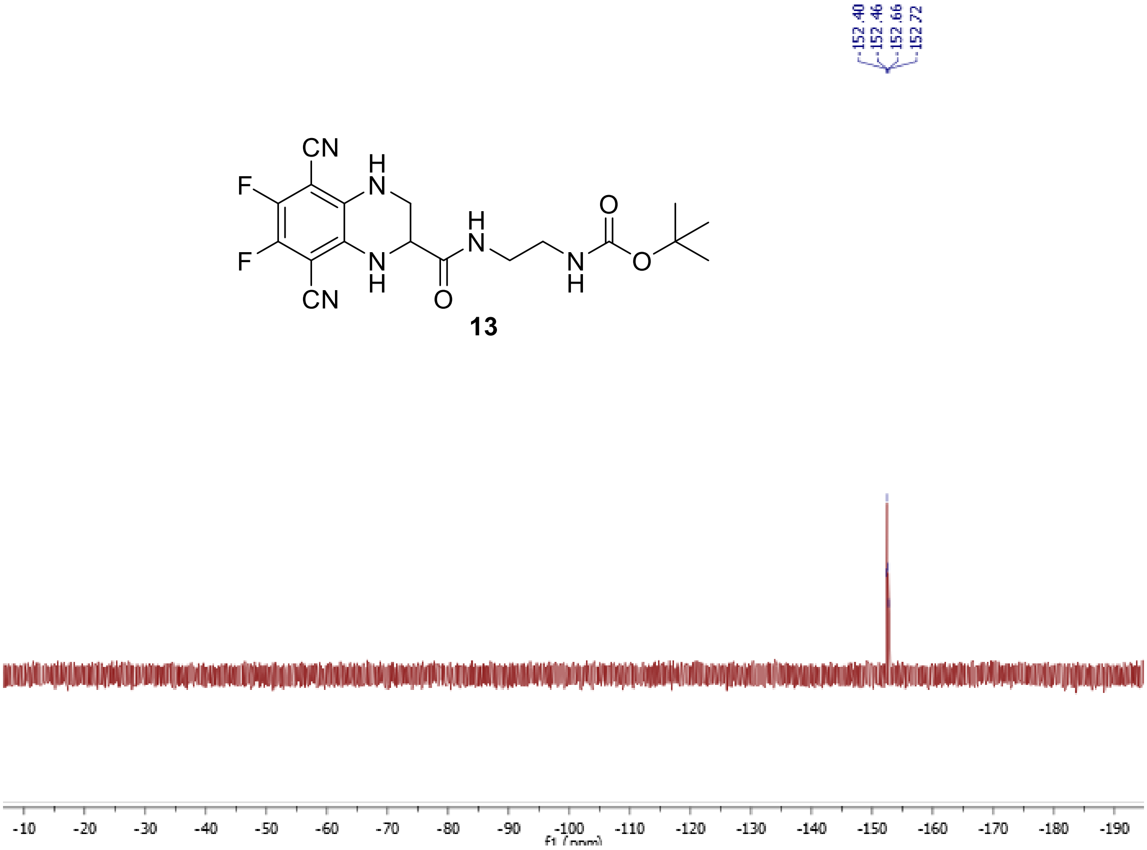

^{1}H NMR (400 MHz, DMSO-d_6)

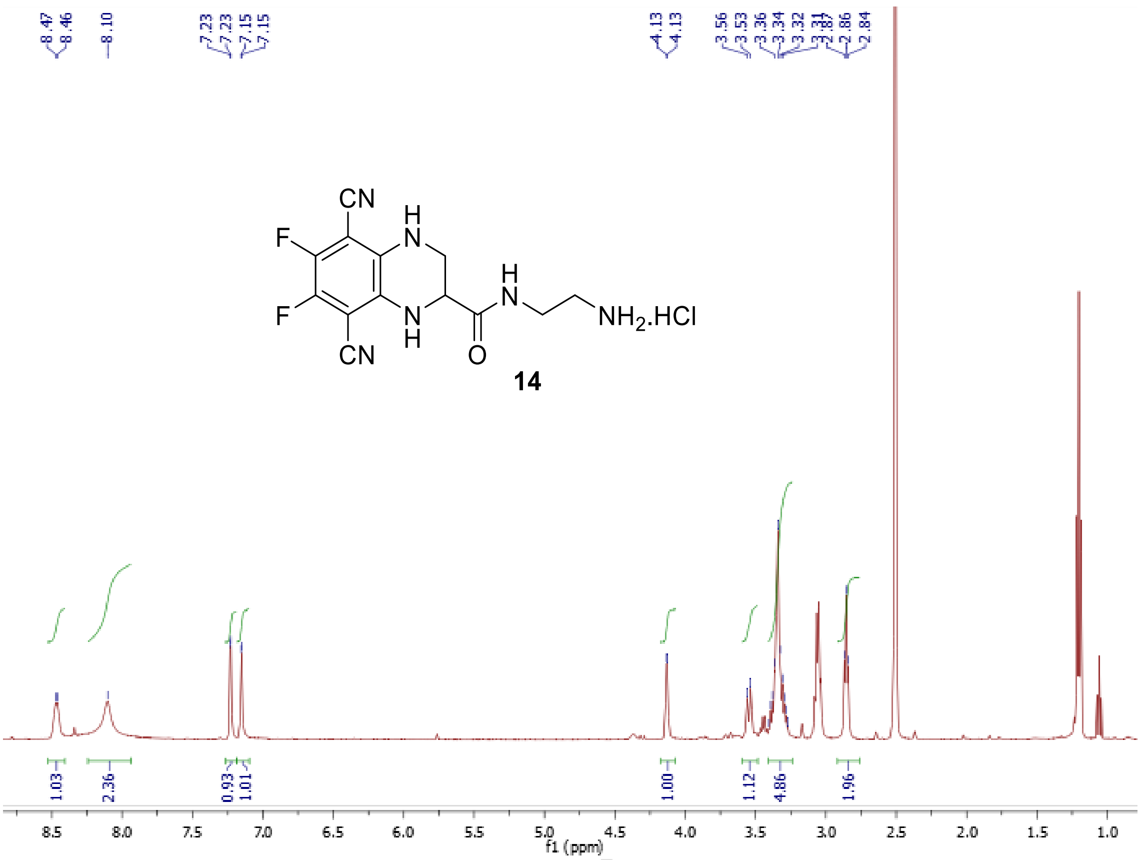

^{13}C NMR (126 MHz, DMSO-d_6)

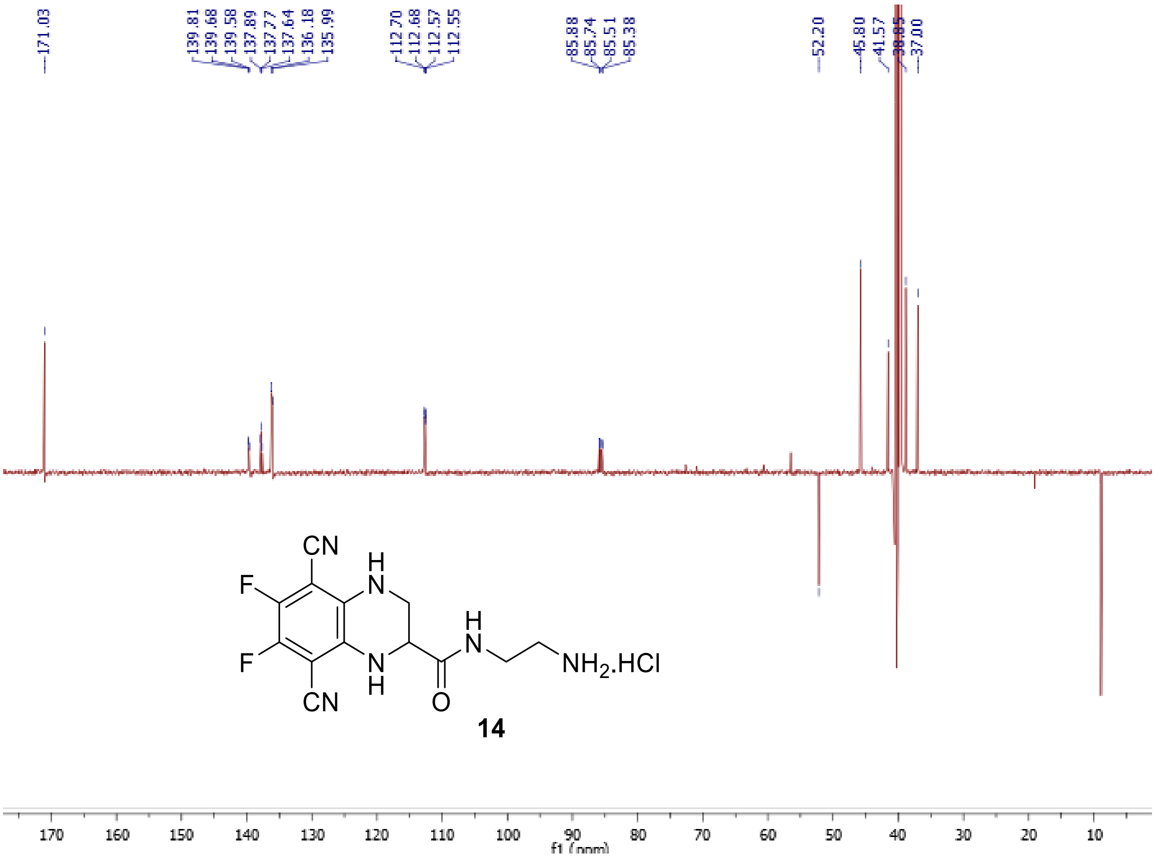

^{19}F NMR (376 MHz, DMSO-d_6)

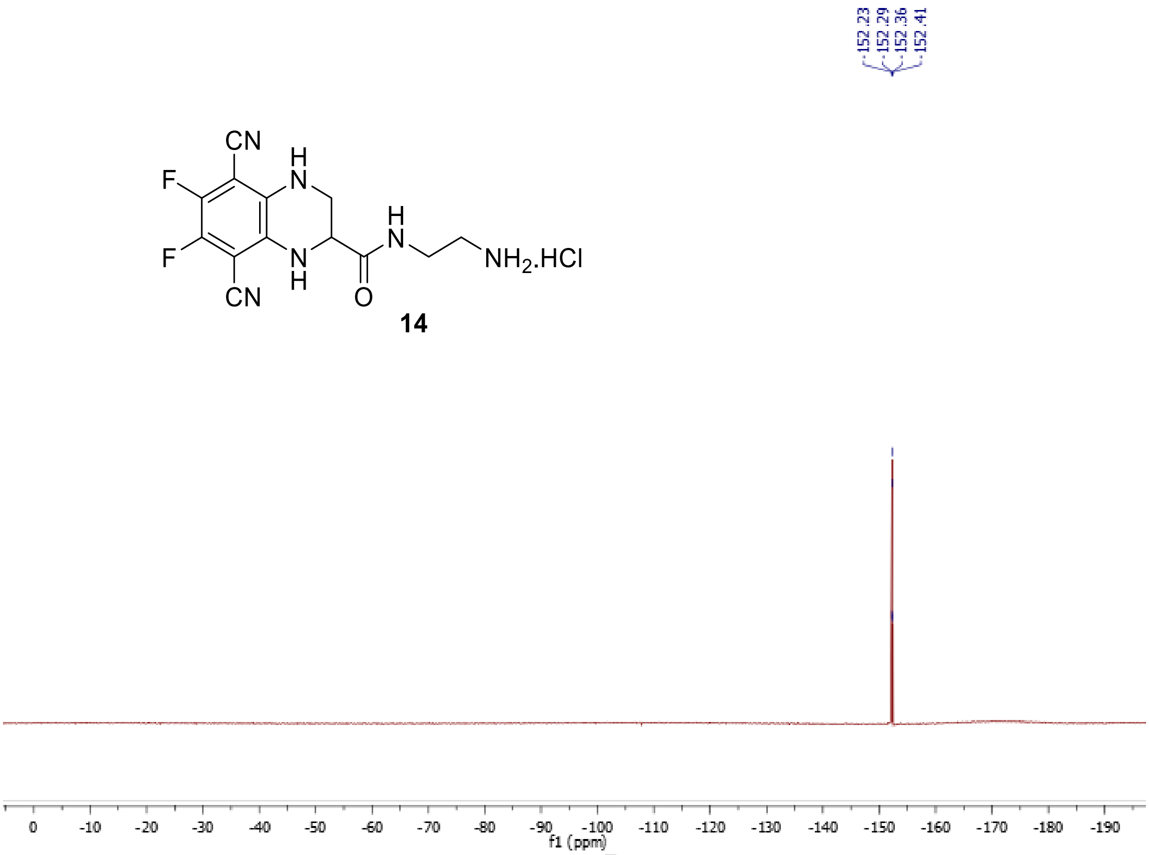

^{1}H NMR (400 MHz, DMSO-d_6)

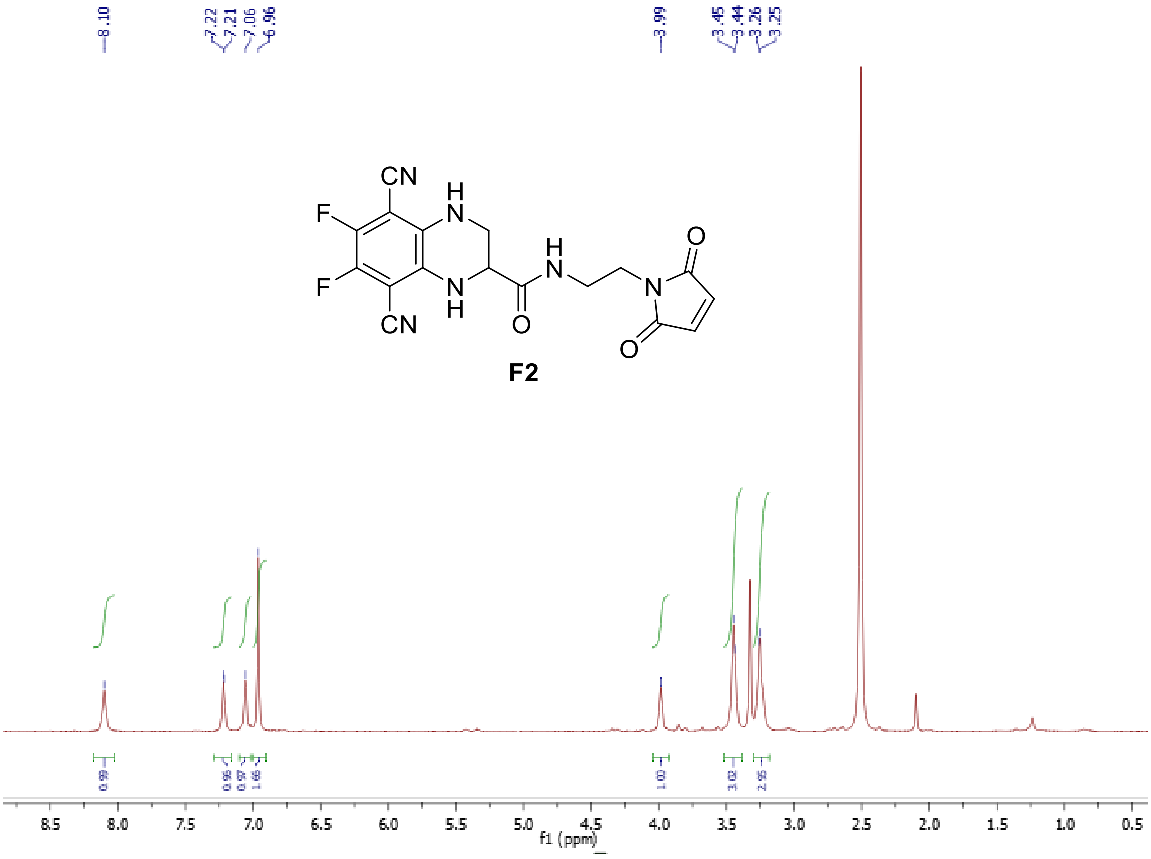

^{13}C NMR (126 MHz, DMSO-d_6)

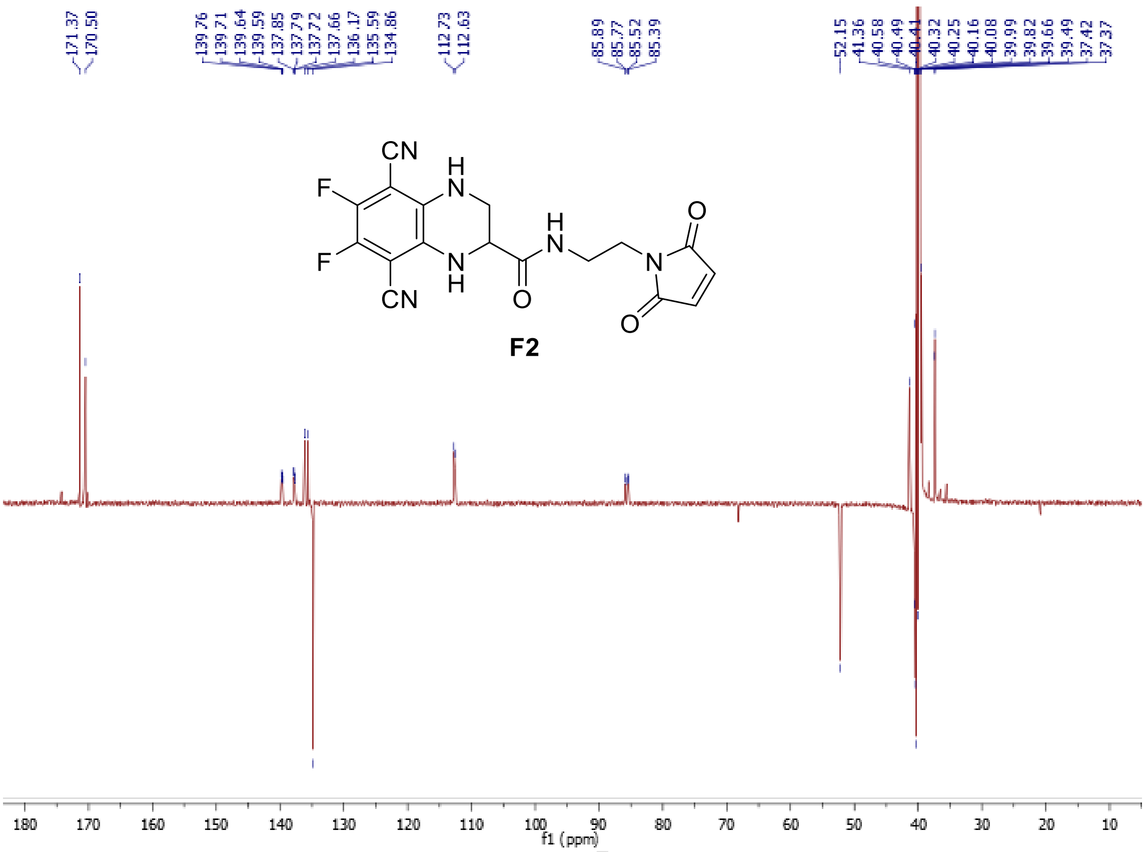

^{19}F NMR (376 MHz, DMSO-d_6)

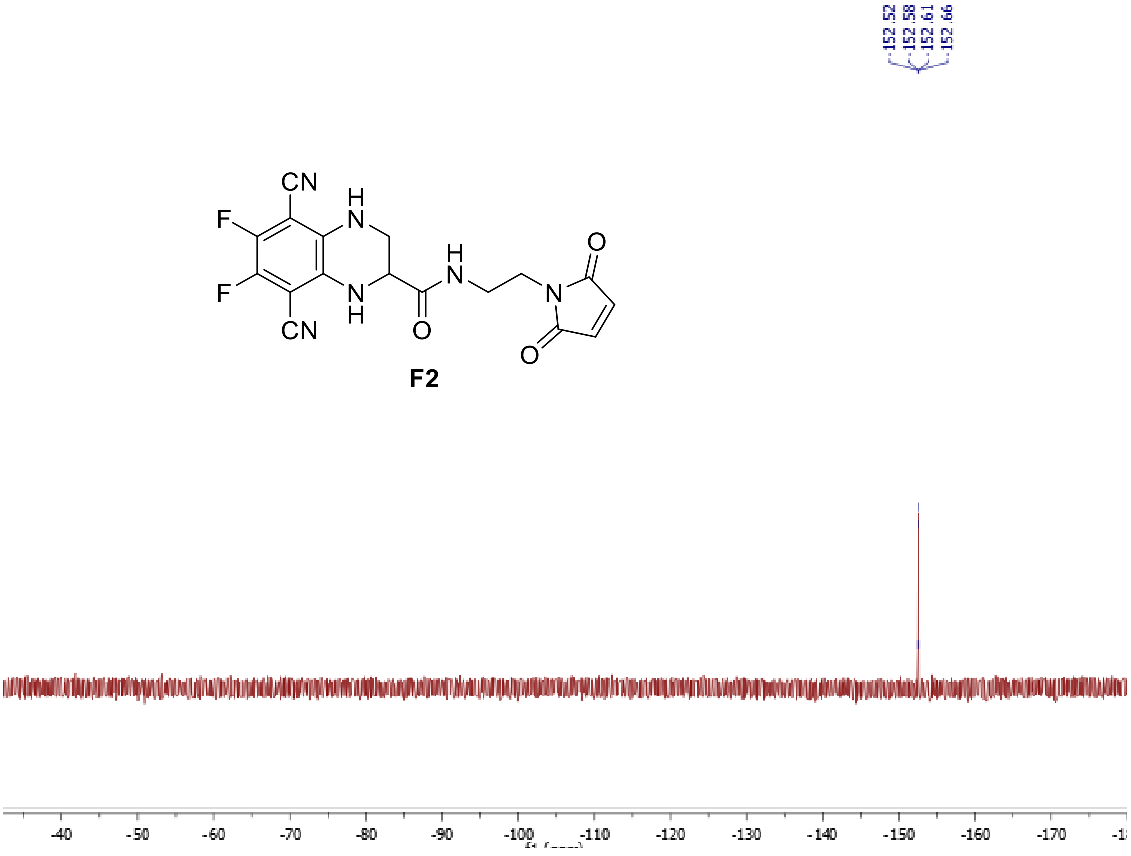

^{1}H NMR (400 MHz, DMSO-d_6)

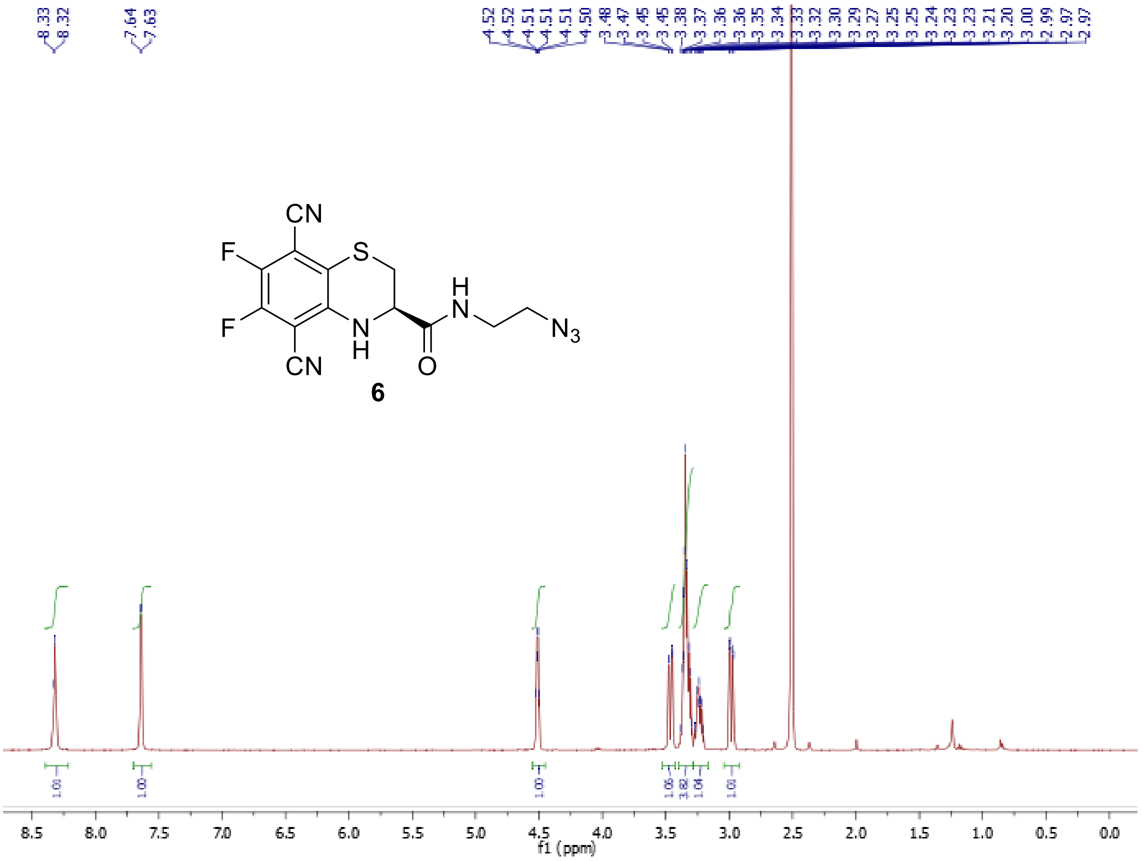

^{13}C NMR (126 MHz, DMSO-d_6)

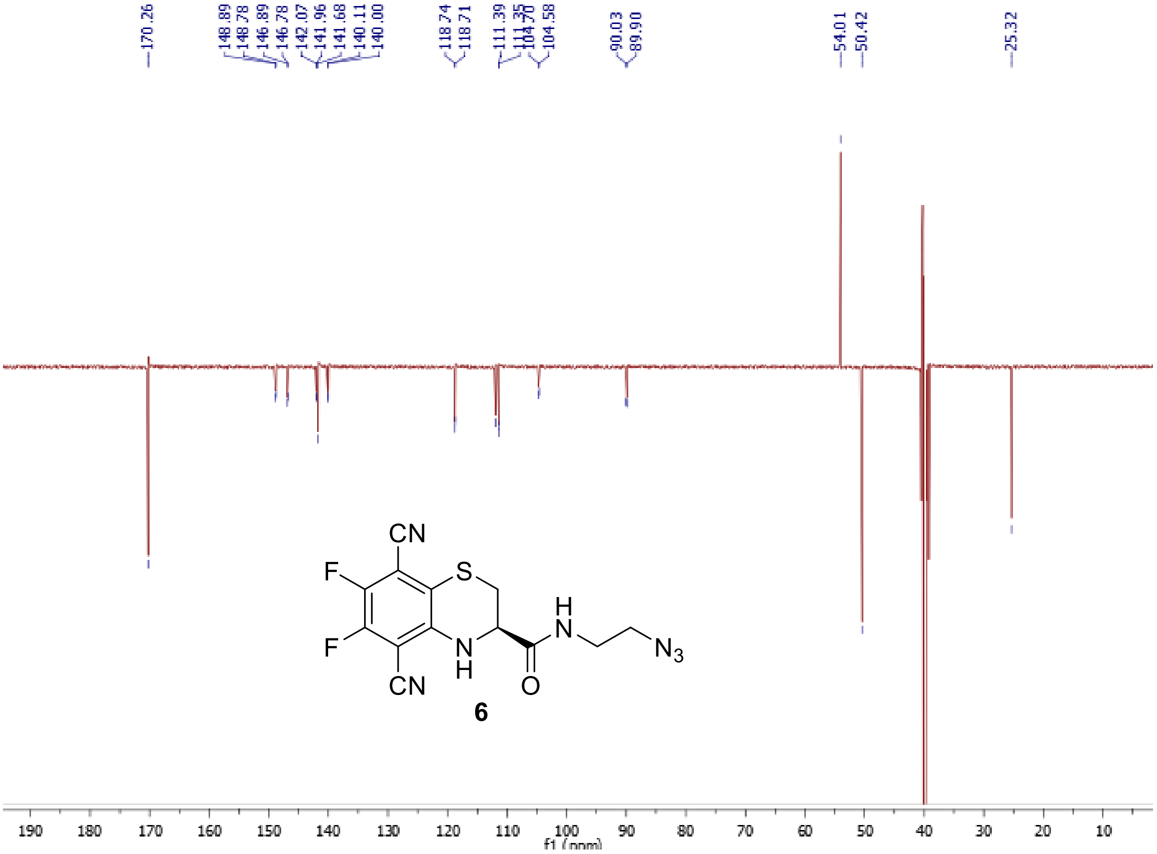

^{19}F NMR (376 MHz, DMSO-d_6)

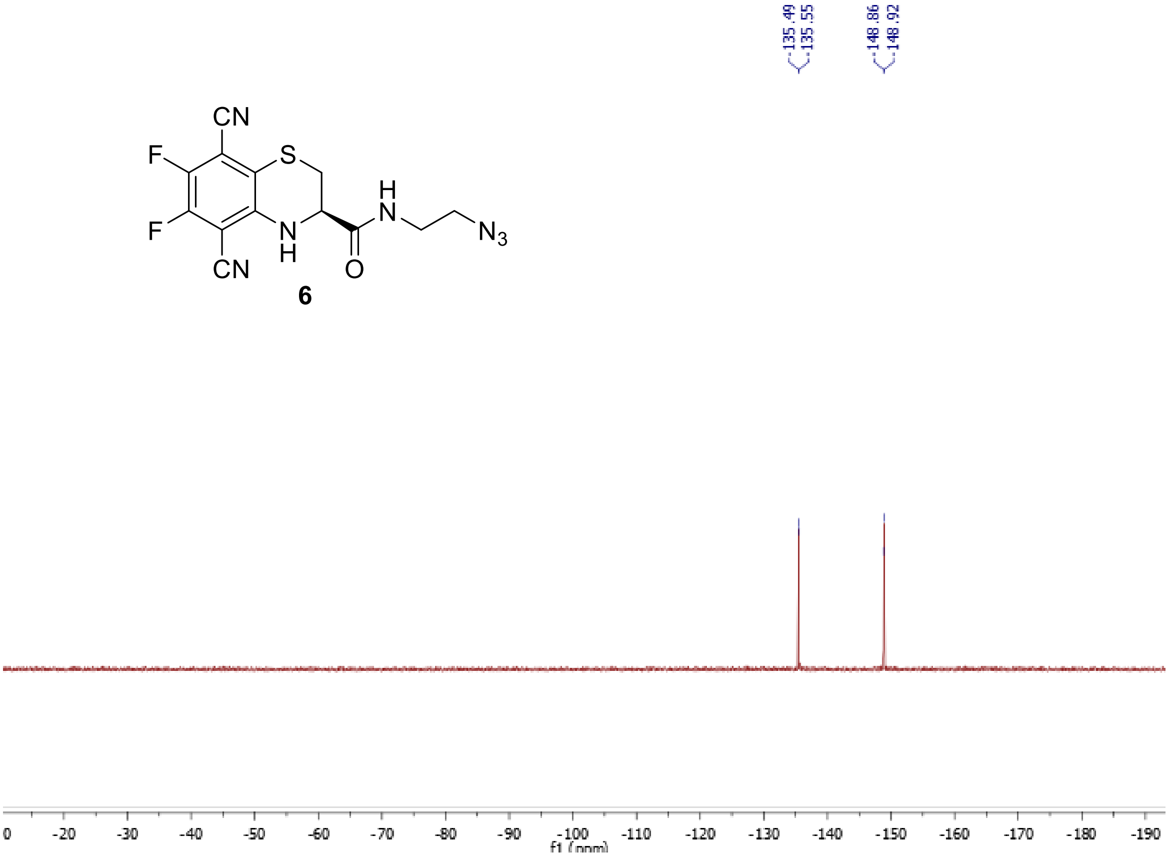

^{1}H NMR (400 MHz, DMSO-d_6)

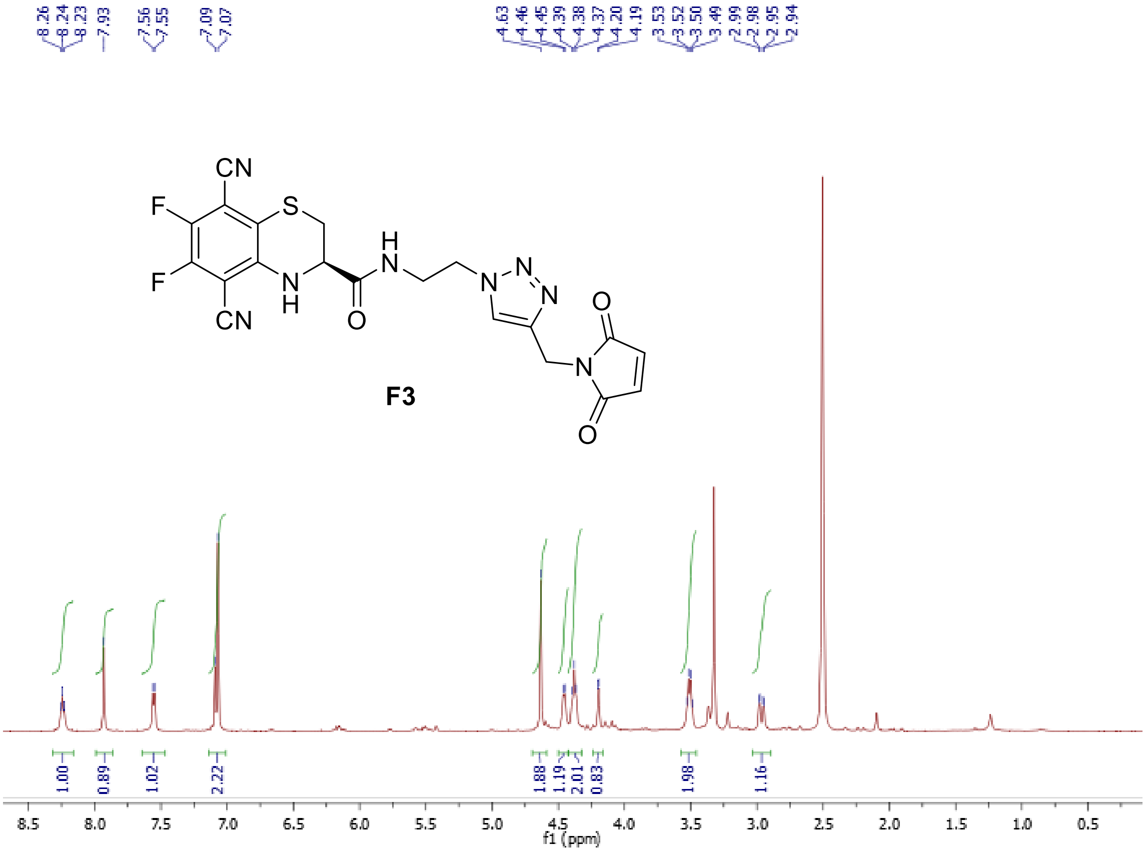

^{13}C NMR (126 MHz, DMSO-d_6)

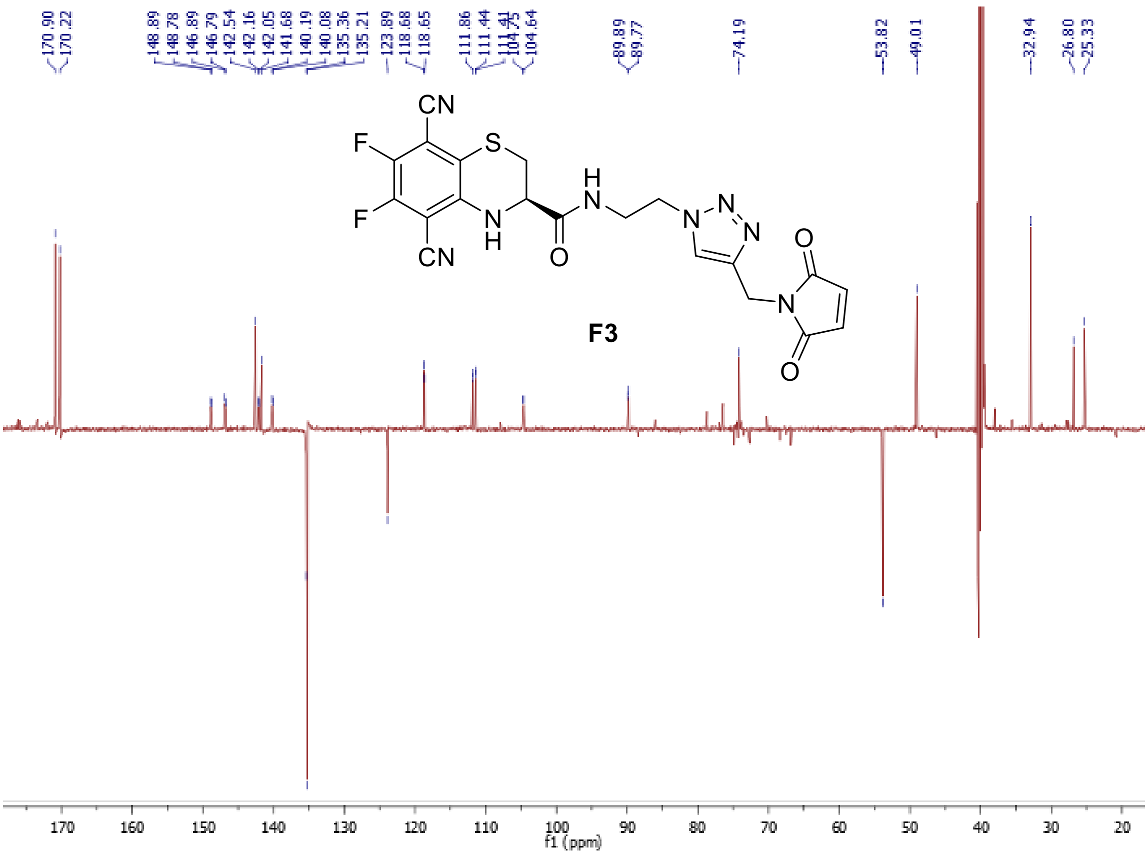

^{19}F NMR (376 MHz, DMSO-d_6)

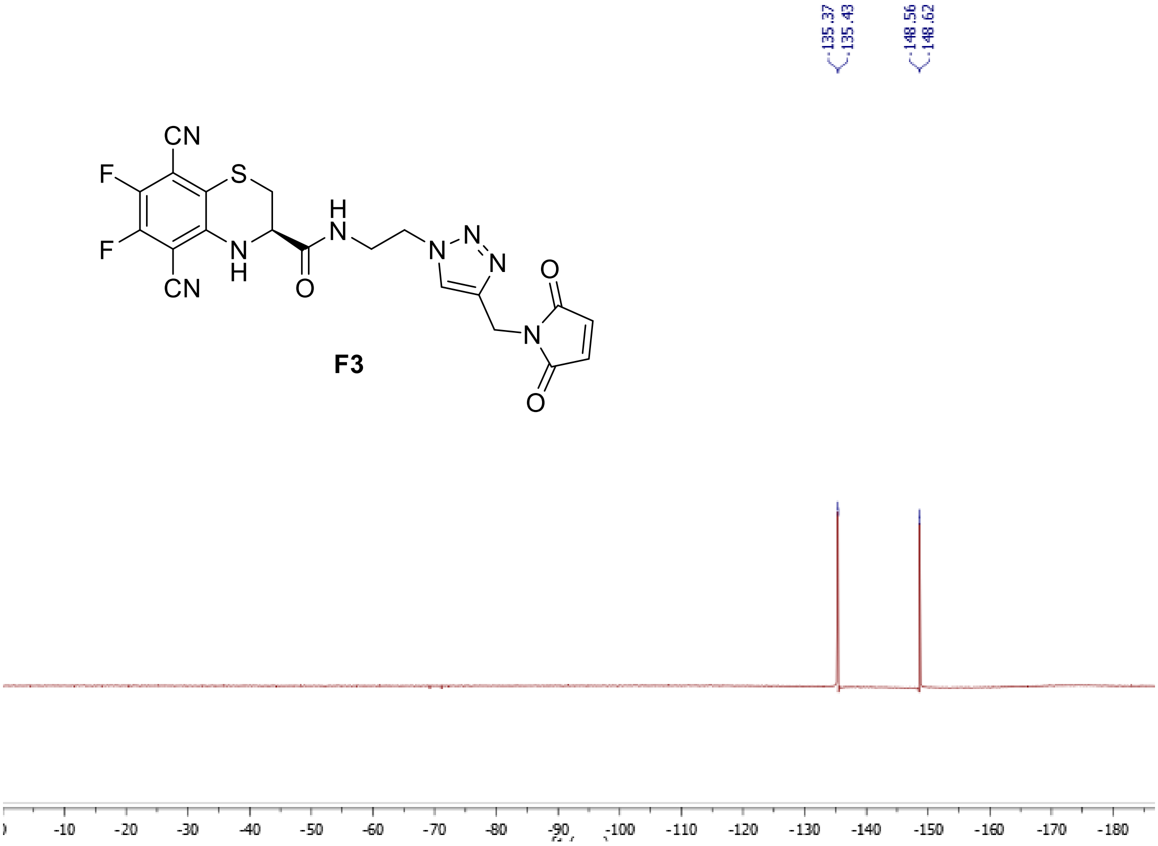

^{1}H NMR (400 MHz, DMSO-d_6)

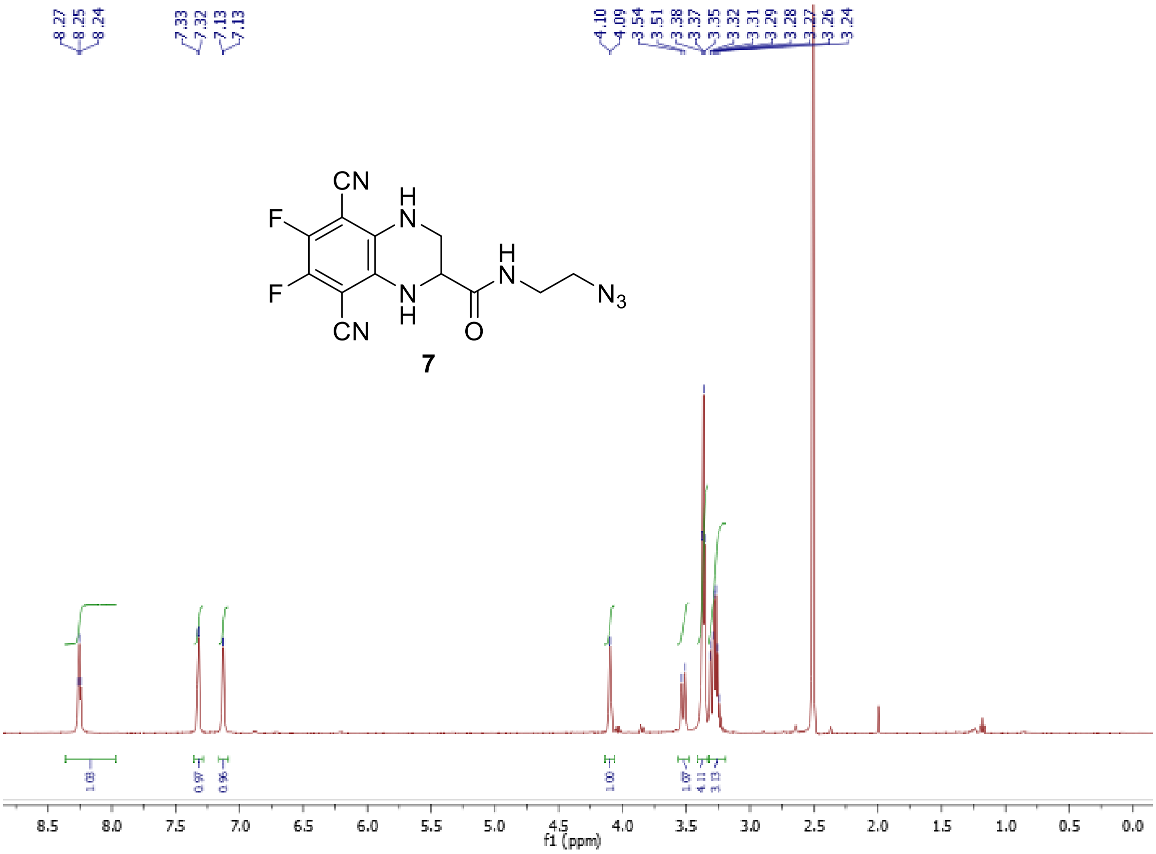

^{13}C NMR (126 MHz, DMSO-d_6)

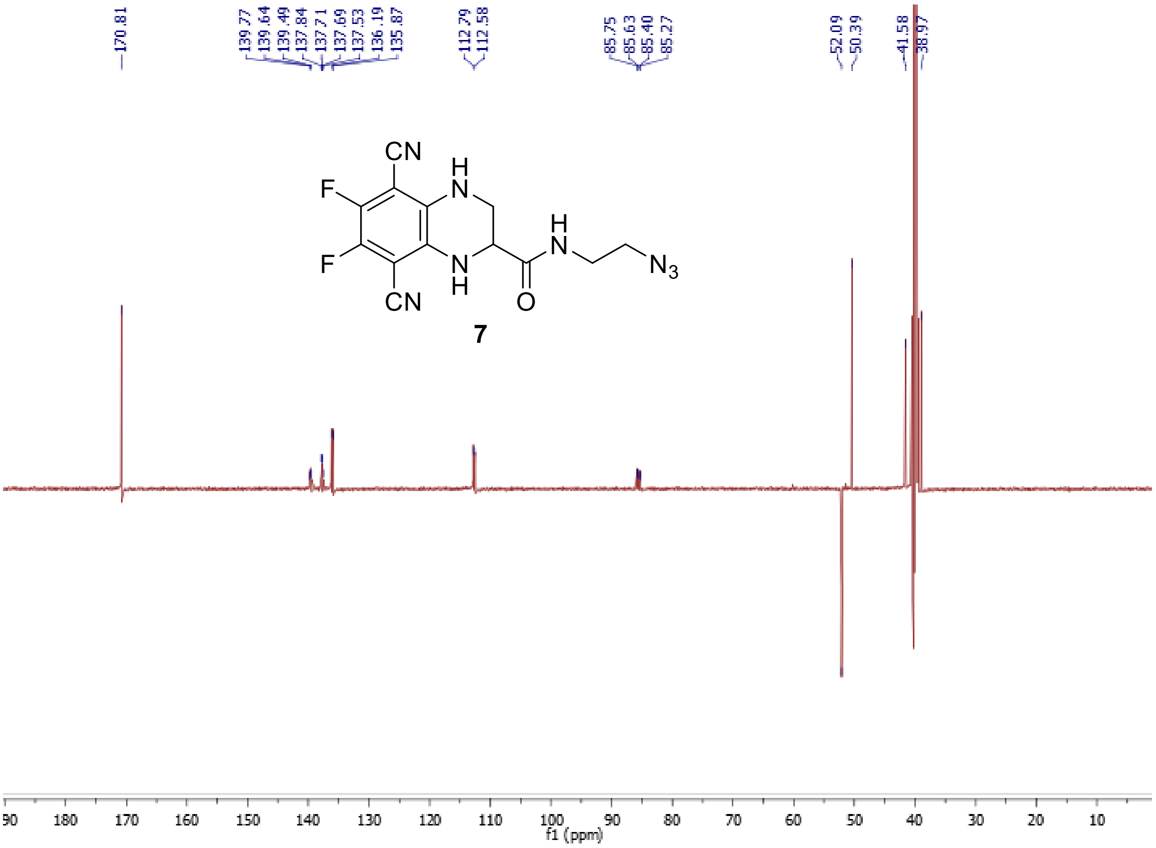

^{19}F NMR (376 MHz, DMSO-d_6)

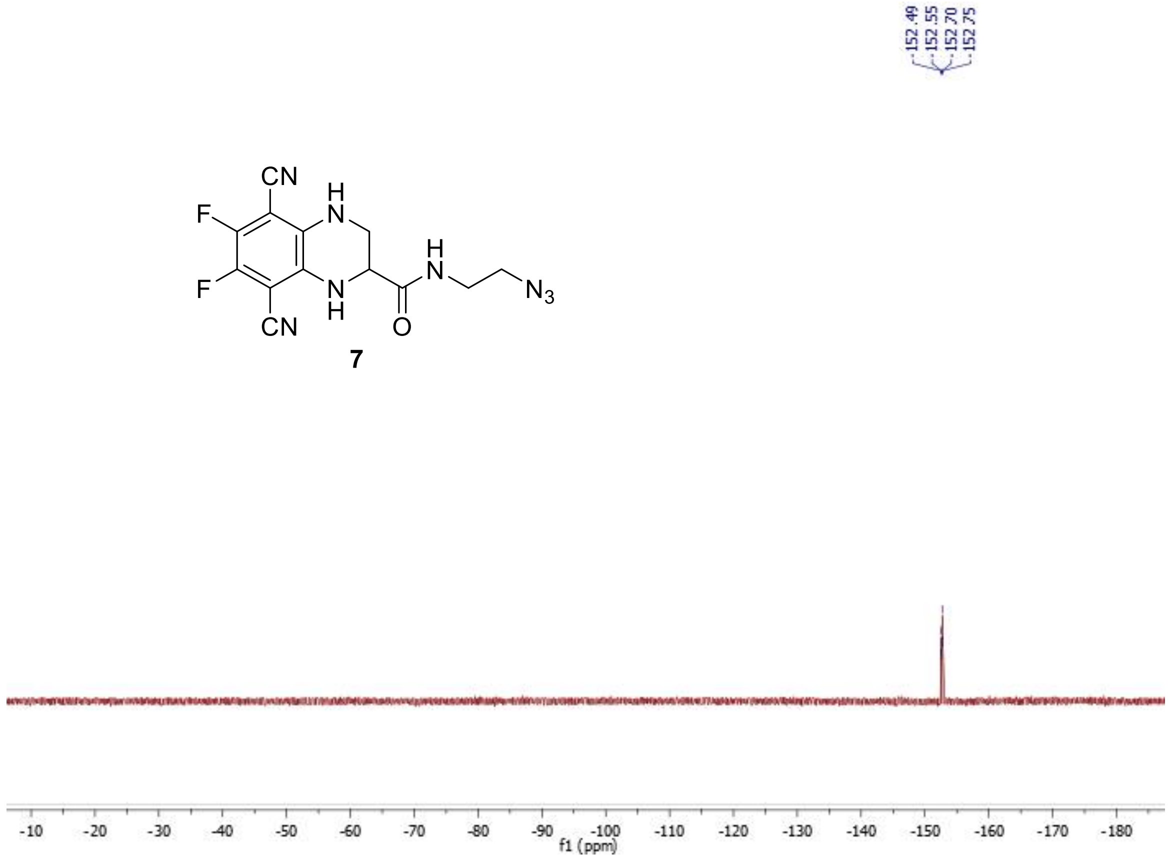

^{1}H NMR (400 MHz, DMSO-d_6)

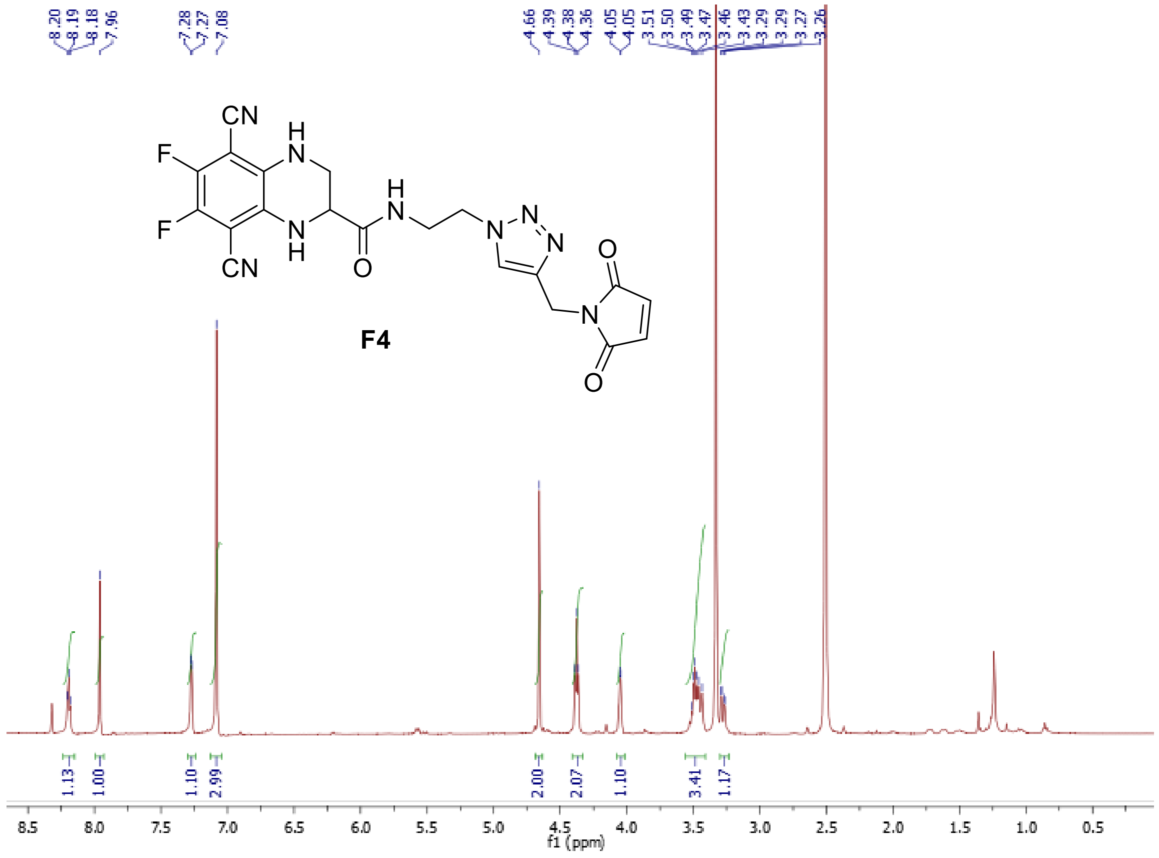

^{13}C NMR (126 MHz, DMSO-d_6)

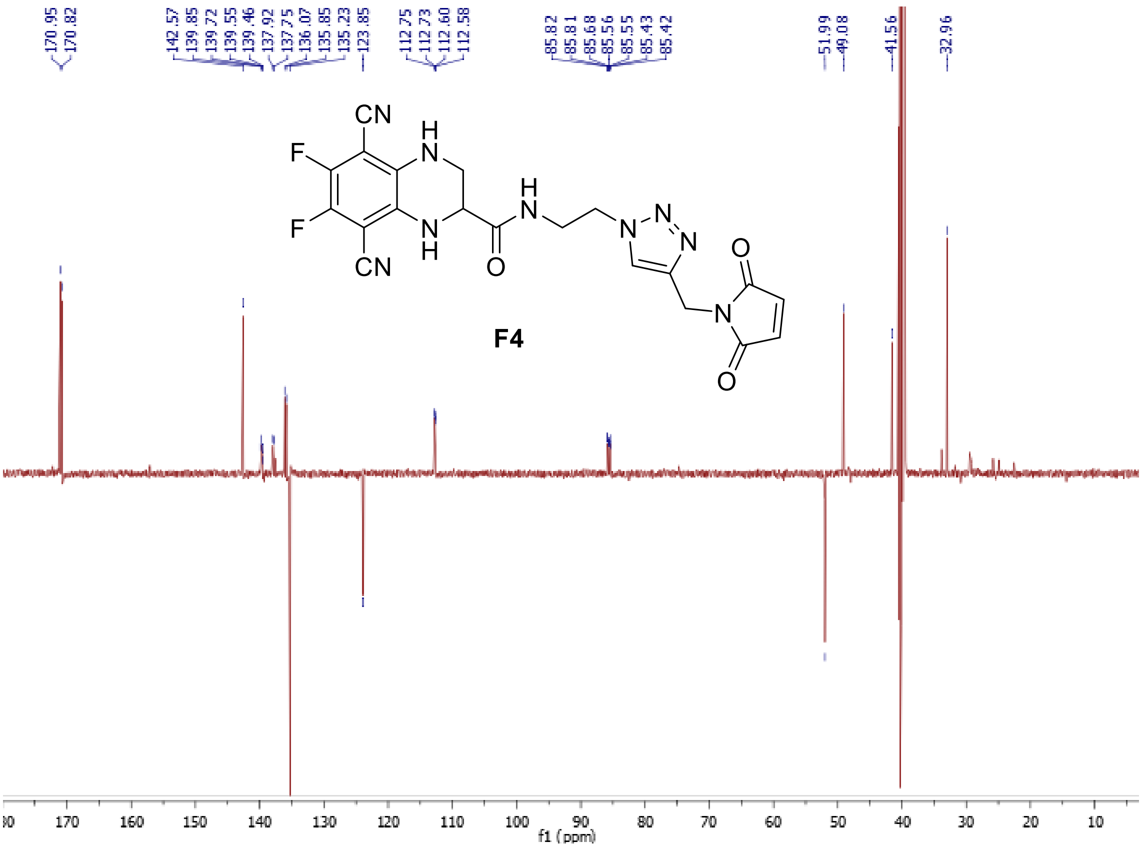

^{19}F NMR (376 MHz, DMSO-d_6)

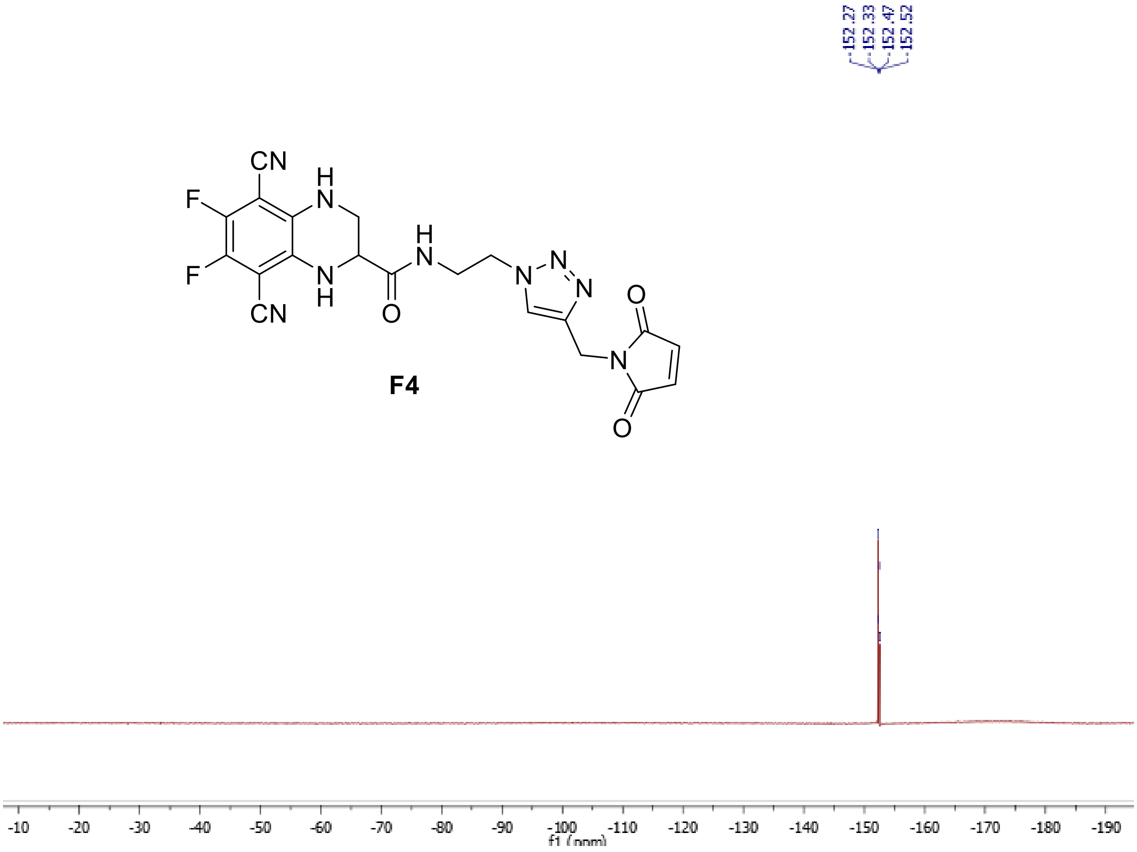

^{1}H NMR (400 MHz, DMSO-d_6)

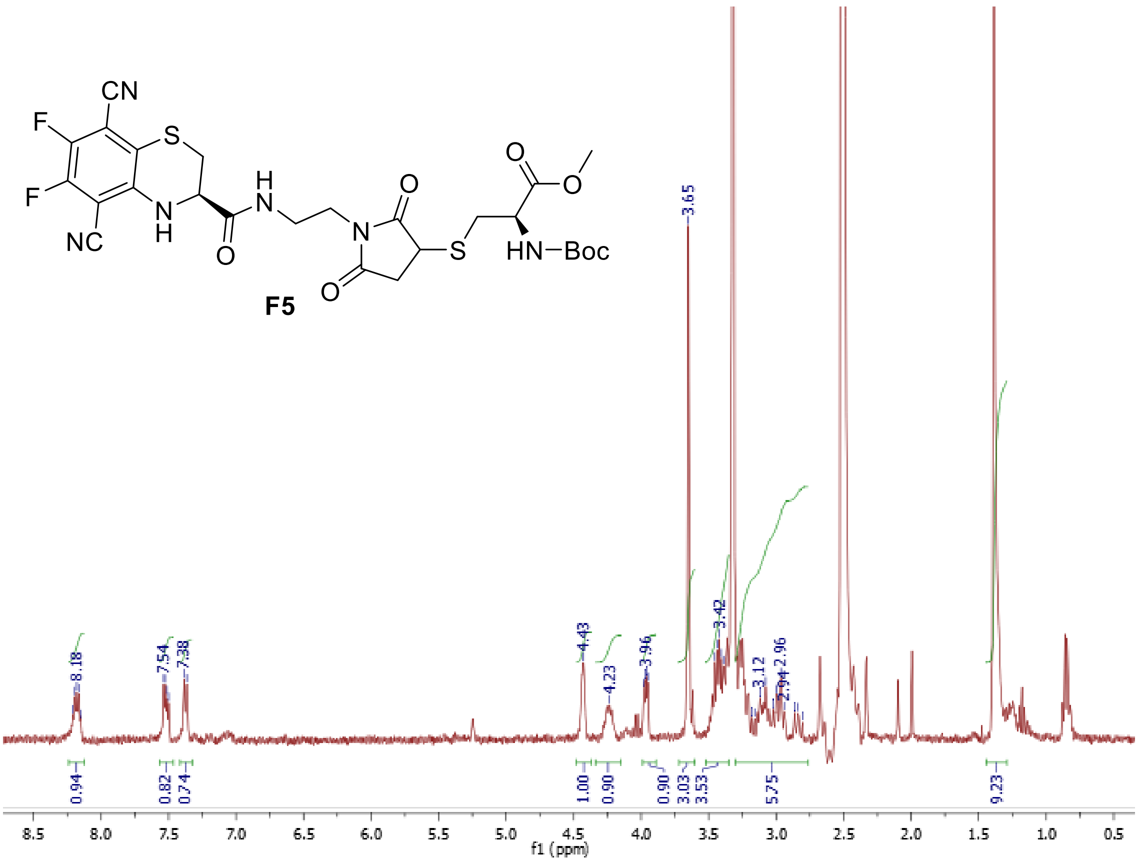

^{13}C NMR (126 MHz, DMSO-d_6)

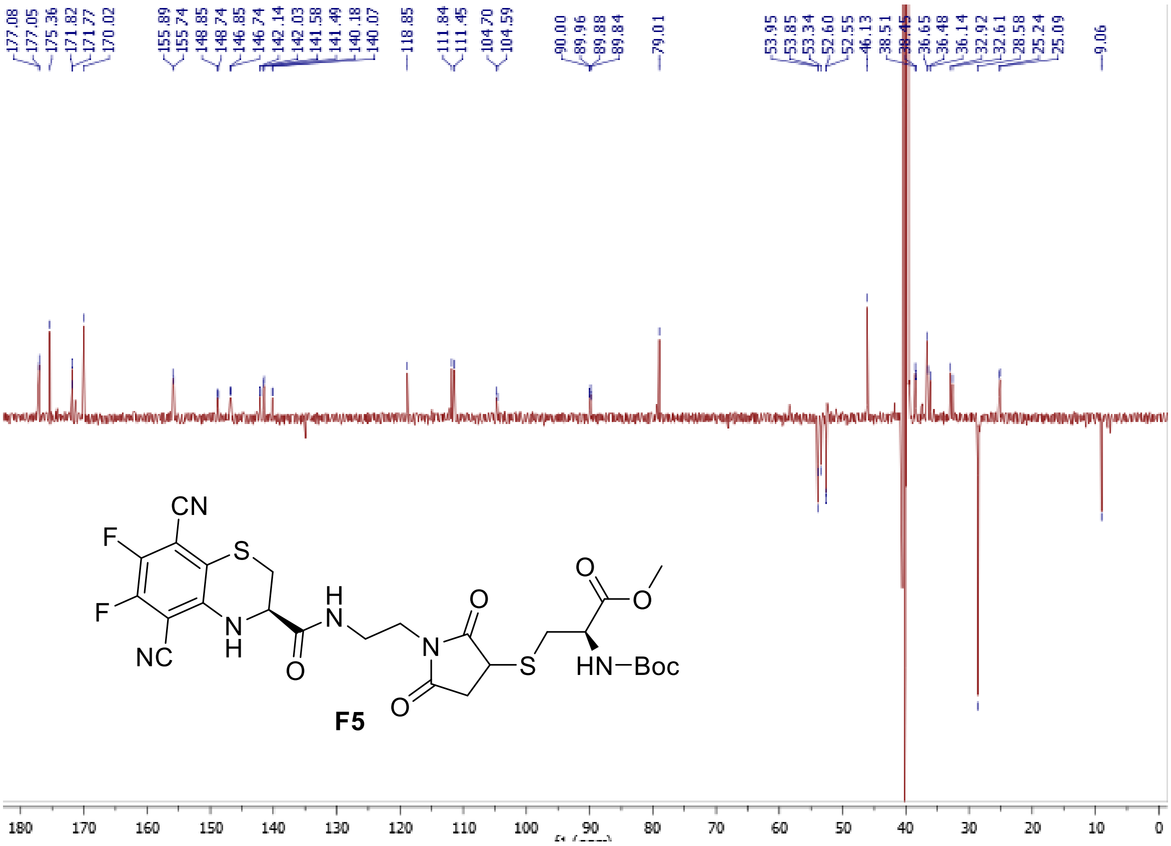

^{19}F NMR (376 MHz, DMSO-d_6)

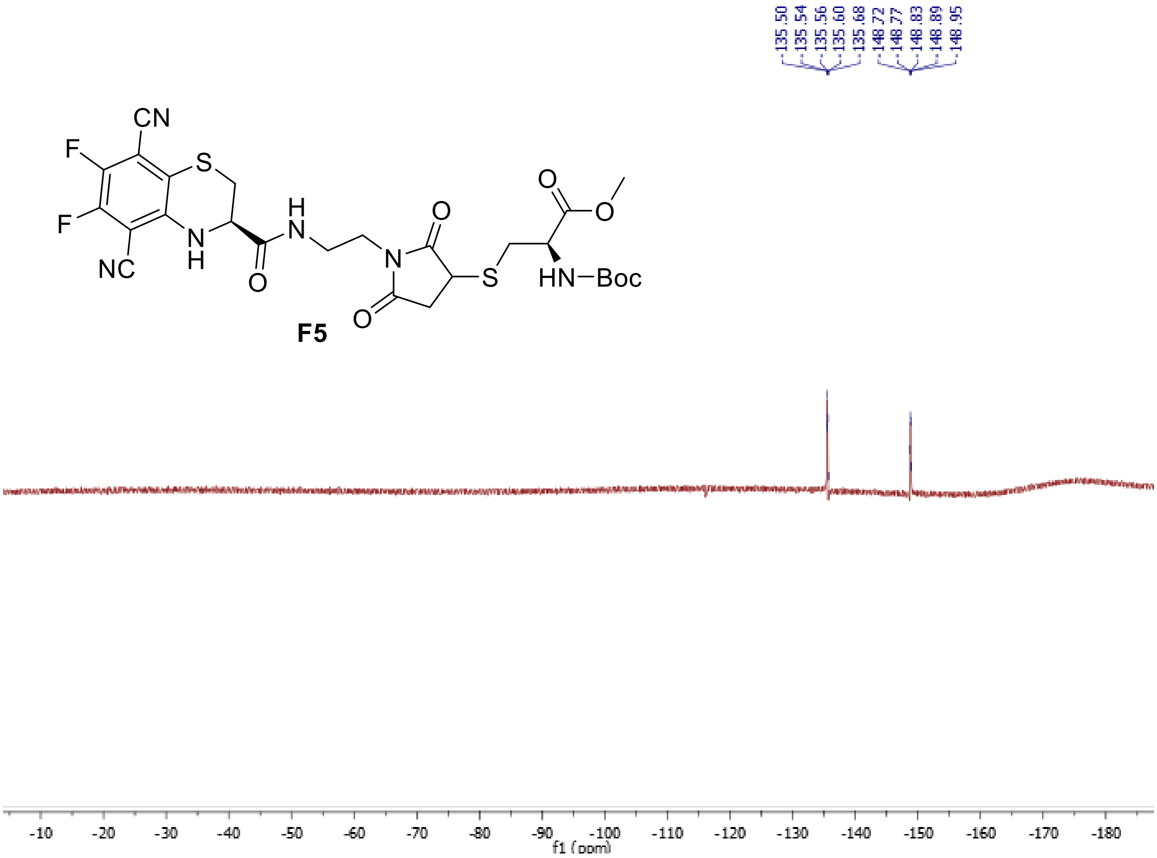

^{1}H NMR (400 MHz, DMSO-d_6)

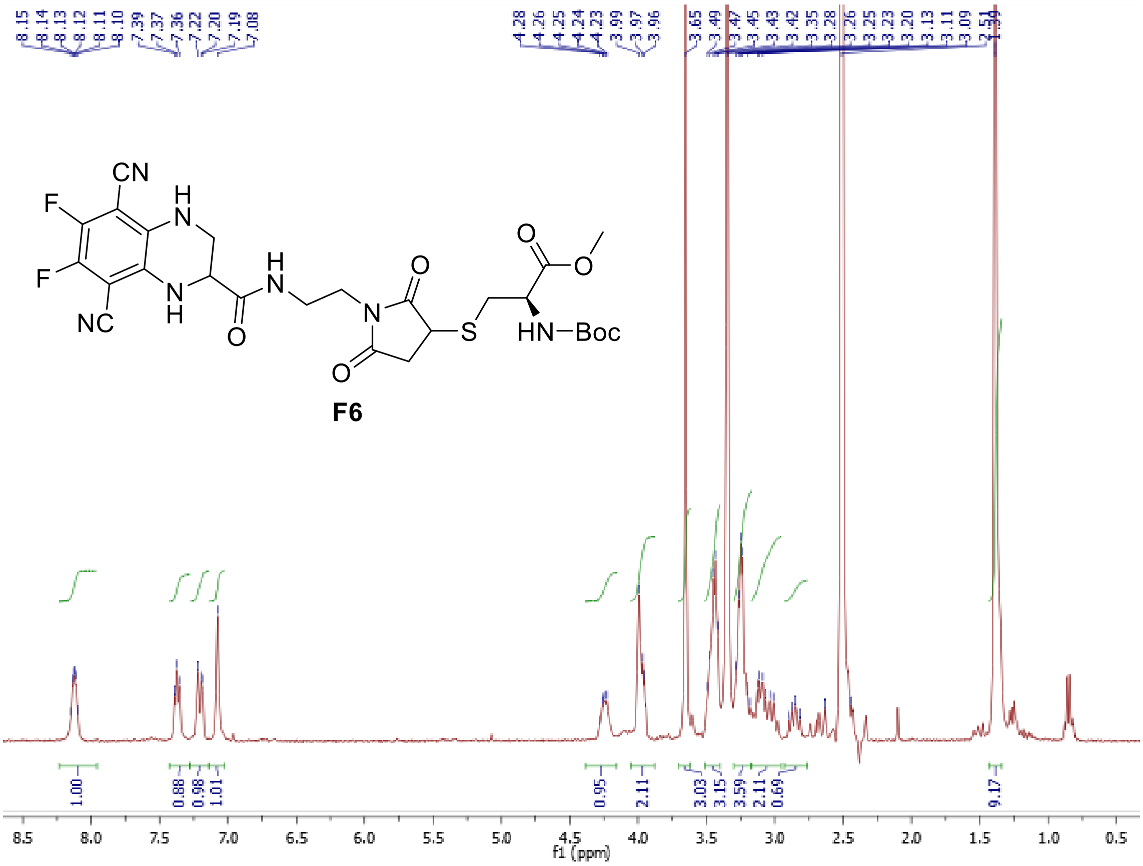

^{13}C NMR (126 MHz, DMSO-d_6)

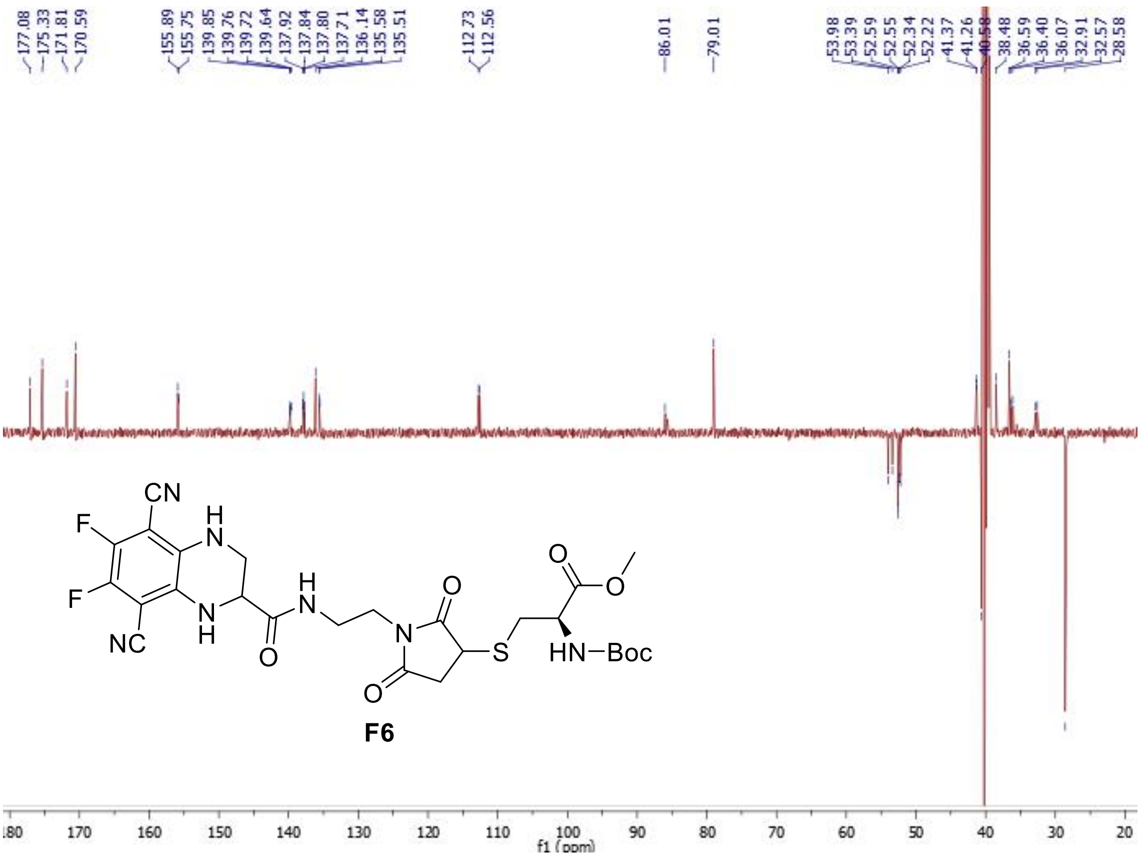

^{19}F NMR (376 MHz, DMSO-d_6)

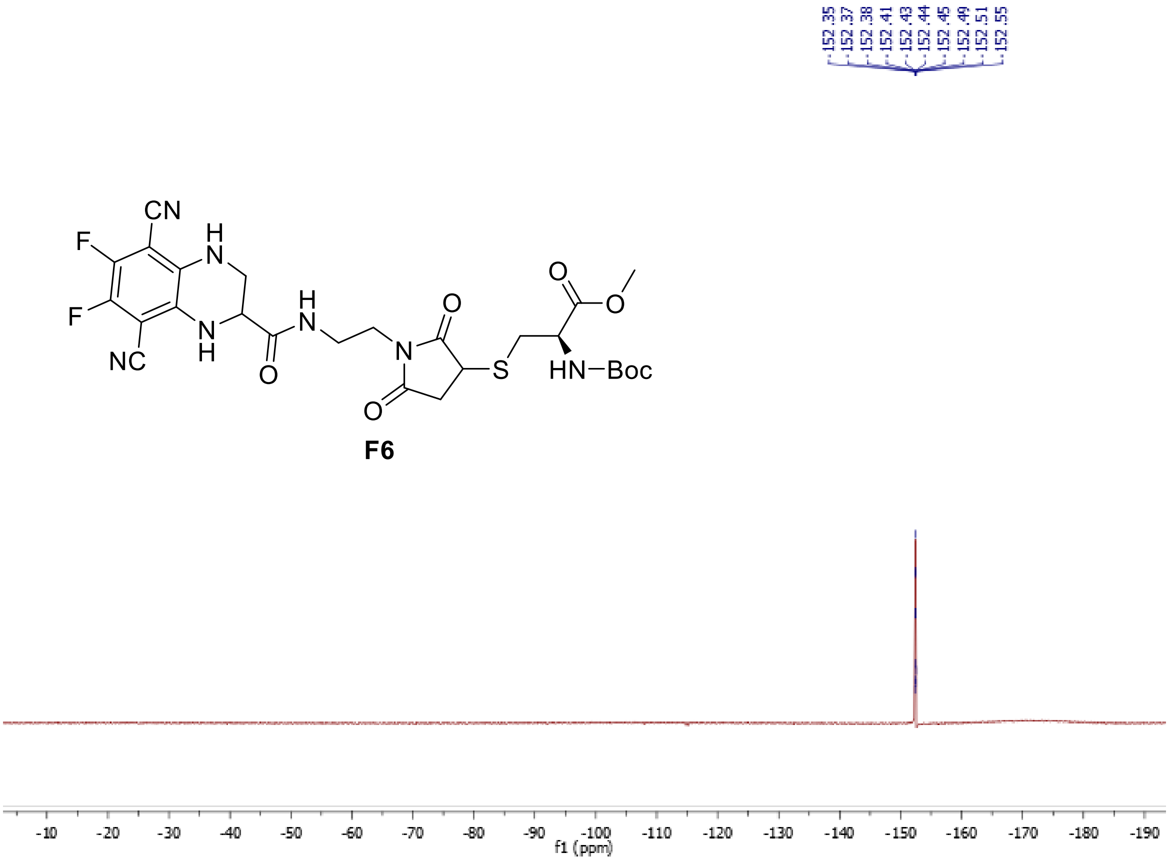

7. References

[18] H. Zhang, R. Liu, J. Liu, L. Li, P. Wang, S. Q. Yao, Z. Xu, H. Sun, *Chem. Sci.* **2016**, *7*, 256.

[29] T. Hatano, L. Sivashanmugam, A. Suchenko, H. Hussain, M. K. Balasubramanian, *J. Cell Sci.* **2020**, *133*, jcs241406.

[30] (a) D. S. Kudryashov, M. Phillips, E. Reisler, *Biophys. J.* **2004**, *87*, 1136; (b) C. K. Chen, S. A. Benchaar, M. Phan, E. E. Grintsevich, R. R. O, Loo, J. A. Loo, E. Reisler, *Biochemistry* **2013**, *52*, 5503.

[34] J. Wang, M. Uttamchandani, J. Li, M. Huand, S. Q. Yao, *Chem. Commun.* **2006**, 3783.

[35] R. Sanichar, J. C. Vederas, *Org. Lett.* **2017**, *19*, 1950.

[36] A. Zaucker, C. A. Mitchell, H. L. E. Coker, K. Sampath, *Front. Cell Dev. Biol.* **2021**, *9*, 712503.

[37] O. V. Dolomanov, L. J. Bourhis, R. L. Gildea, J. A. K. Howard, H. Puschmann, *J. Appl. Cryst.* **2009**, *42*, 339.

[38] G. M. Sheldrick, *Acta Cryst.* **2015**, *A71*, 3.
